# NMR Screen Reveals the Diverse Structural Landscape of a G‐Quadruplex Library

**DOI:** 10.1002/chem.202401437

**Published:** 2024-11-11

**Authors:** Ráchel Sgallová, Martin Volek, Jaroslav Kurfürst, Pavel Srb, Václav Veverka, Edward A. Curtis

**Affiliations:** ^1^ Institute of Organic Chemistry and Biochemistry Prague Czech Republic; ^2^ Department of Low-Temperature Physics Faculty of Mathematics and Physics Charles University in Prague Prague Czech Republic; ^3^ Department of Genetics and Microbiology Faculty of Science Charles University in Prague Prague Czech Republic; ^4^ Department of Informatics and Chemistry University of Chemistry and Technology Prague Czech Republic; ^5^ Department of Cell Biology, Faculty of Science Charles University in Prague Prague Czech Republic

**Keywords:** DNA, G-quadruplex, Multimeric structures, NMR

## Abstract

G‐quadruplexes are noncanonical nucleic acid structures formed by stacked guanosine tetrads. Despite their functional and structural diversity, a single consensus model is typically used to describe sequences with the potential to form G‐quadruplex structures. We are interested in developing more specific sequence models for G‐quadruplexes. In previous work, we functionally characterized each sequence in a 496‐member library of variants of a monomeric reference G‐quadruplex for the ability to bind GTP, promote a model peroxidase reaction, generate intrinsic fluorescence, and to form multimers. Here we used NMR to obtain a broad overview of the structural features of this library. After determining the ^1^H NMR spectrum of each of these 496 sequences, spectra were sorted into multiple classes, most of which could be rationalized based on mutational patterns in the primary sequence. A more detailed screen using representative sequences provided additional information about spectral classes, and confirmed that the classes determined based on analysis of ^1^H NMR spectra are correlated with functional categories identified in previous studies. These results provide new insights into the surprising structural diversity of this library. They also show how NMR can be used to identify classes of sequences with distinct mutational signatures and functions.

## Graphical abstract

G‐quadruplexes are noncanonical nucleic acid structures with a range of biochemical functions. Here we used NMR to obtain a broad overview of the structural features of a 496‐member G‐quadruplex library. Our work highlights the surprising diversity of this library, and shows how NMR can be used to identify classes of sequences with distinct mutational signatures and functions.

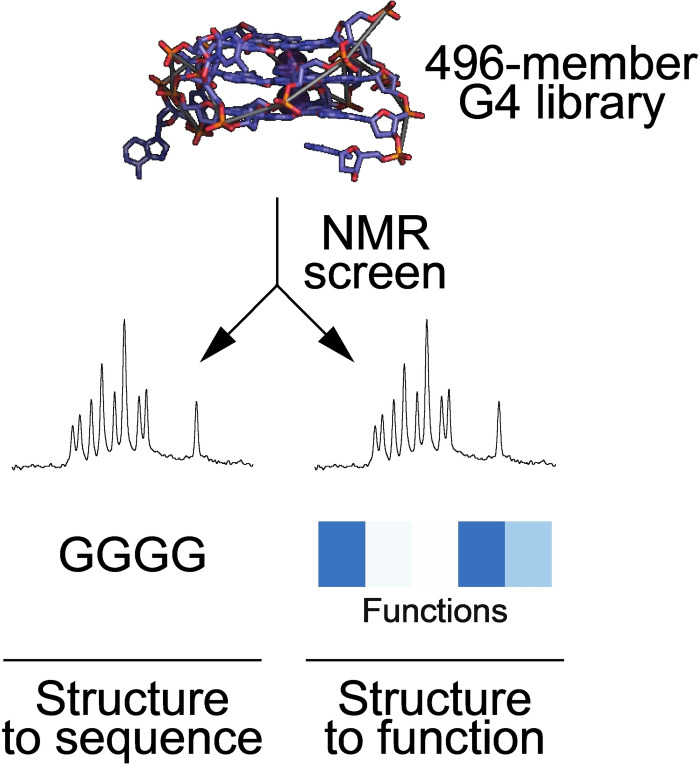

## Introduction

The most well‐known structure formed by DNA is the double helix. However, this is not the only possibility. Another important type of DNA fold is the G‐quadruplex.^[1]^ This is a four‐stranded structure made up of stacked guanosine tetrads connected by loops. A sequence is typically classified as a potential G‐quadruplex forming sequence if it is consistent with the consensus sequence G_3+_N_1–7_G_3+_N_1–7_G_3+_N_1–7_G_3+_, where G_3+_ is a track of three or more guanosines and N_1–7_ is stretch of up to seven nucleotides of any sequence.^[2]^ The G's in this sequence form tetrads while the N's form loops that connect stretches of guanosines in parallel, antiparallel, or mixed topologies. However, this definition does not describe all G‐quadruplexes, and recent reports suggest that bulges,[^3^, ^4^] G‐triads,^[5]^ long loops,^[6]^ or non‐canonical tetrads[^7^, ^8^] can sometimes be incorporated into G‐quadruplex structures. G‐quadruplexes occur frequently in the genomes of higher eukaryotes (including the human genome), and appear to be biologically important. Potential G‐quadruplex forming sequences frequently occur close to replication origins^[9]^ and in telomeres,^[2]^ act as obstacles to replication forks and polymerases [10], and bind to many biologically important small molecules^[11]^ and proteins.^[12]^ G‐quadruplexes have a wide range of biochemical activities such as intrinsic fluorescence.[^13^, ^14^, ^15^] Some can also catalyze peroxidase reactions in the presence of hemin and hydrogen peroxidase,[^16^, ^17^] and are also increasingly used as scaffolds in biotechnology.^[18]^

The discrepancy between the large number of structures and functions of G‐quadruplexes and the single consensus sequence typically used to identify G‐quadruplexes is striking. We suggest that more specific models made up of subsets of G‐quadruplexes with similar properties would more accurately describe the complex relationship among G‐quadruplex sequence, structure, and function. Such models could also facilitate identification and discovery of G‐quadruplexes with specific biological roles by making it possible to distinguish functionally distinct categories of G‐quadruplexes that are grouped together by current models. Another unresolved issue is the extent to which sequences that differ from that of the G‐quadruplex consensus can form G‐quadruplex structures. To investigate these important questions, our group has been characterizing the properties of a 496‐member library of mutational variants of a monomeric reference G‐quadruplex. This library is made up of four smaller libraries. The first is a tetrad library, which contains each of 256 possible variants of the central tetrad of a monomeric reference G‐quadruplex with a known three‐dimensional structure.^[19]^ The second is the 17.3 loop library, which contains each of the 81 possible loop variants (A, C, or T but not G) of the reference G‐quadruplex. The third is the 17.4 loop library, which contains each of the 81 possible loop variants (A, C, or T but not G) of a representative dimer‐forming sequence from the library. The fourth is the 17.10 loop library, which contains each of the 81 possible loop variants (A, C, or T but not G) of a representative tetramer‐forming sequence from the library. We note that not all of these sequences are expected to form G‐quadruplexes, and that a library designed in this way can therefore provide information about both mutations that are compatible with G‐quadruplex structure and function and those that are not. In previous studies we focused on the functional properties of this library, and tested each of the 496 sequences for the ability to bind GTP, promote a model peroxidase reaction, generate intrinsic fluorescence, form dimers, and form tetramers.[^15^, ^19^, ^20^, ^21^, ^22^] These studies showed that the sequence requirements of the G‐quadruplexes in the library are overlapping (sequences in the library often have multiple activities) but distinct (the subset of sequences with one activity never perfectly overlaps with the subset of sequences with a second activity).^[19]^ They also indicated that biochemical functions are correlated with both primary sequence and multimeric state. However, they provided only limited structural information about library members.

To address this limitation, here we characterized the structural features of each of the sequences in the library using ^1^H NMR. A number of experimental techniques could have in principle been used for this screen,^[23]^ including circular dichroism,^[24]^ UV melting,^[25]^ FRET,^[26]^ mass spectrometry,^[27]^ and X‐ray crystallography.^[28]^ A significant advantage of NMR is that it can be used to study many different aspects of G‐ quadruplex structure.^[29]^ For example, NMR can be used to confirm that a sequence forms a G‐quadruplex, to determine its high‐resolution structure, and to study its folding pathway.[^30^, ^31^, ^32^] More detailed experiments can also be performed using samples identified as interesting based on their ^1^H NMR spectra. Although often limited to the study of tens of sequences,^[33]^ with the help of an automated sample changer we were able to characterize a library of 496 variants of the reference G‐quadruplex in this study. Our screen revealed that the library contains multiple classes made up of sequences with distinct ^1^H NMR spectra, including classes that were not previously identified based on low‐resolution techniques such as native PAGE. Sequences in these classes often have distinct mutational signatures and biochemical properties, which provides additional support for the idea that the commonly used consensus sequence for G‐quadruplexes is too general. Nucleotides that form the central tetrad of the monomeric G‐quadruplex used as a starting point for the library are particularly important with respect to both structure and function, and the positions of mutations in this tetrad is the most important parameter determining the class into which a sequence belongs. Several sequences with surprising properties were also identified in this screen, including slow folding G‐quadruplexes and sequences that contained three or four mutated positions in the central tetrad of the reference G‐ quadruplex but nevertheless contained G‐quadruplex‐like signals in the ^1^H NMR spectra. Taken together, these results highlight the remarkable structural and functional diversity of this library and set the stage for future high‐resolution structural characterization of representative sequences with interesting properties.

## Results and Discussion

### Workflow of the Study

**Figure 1:**
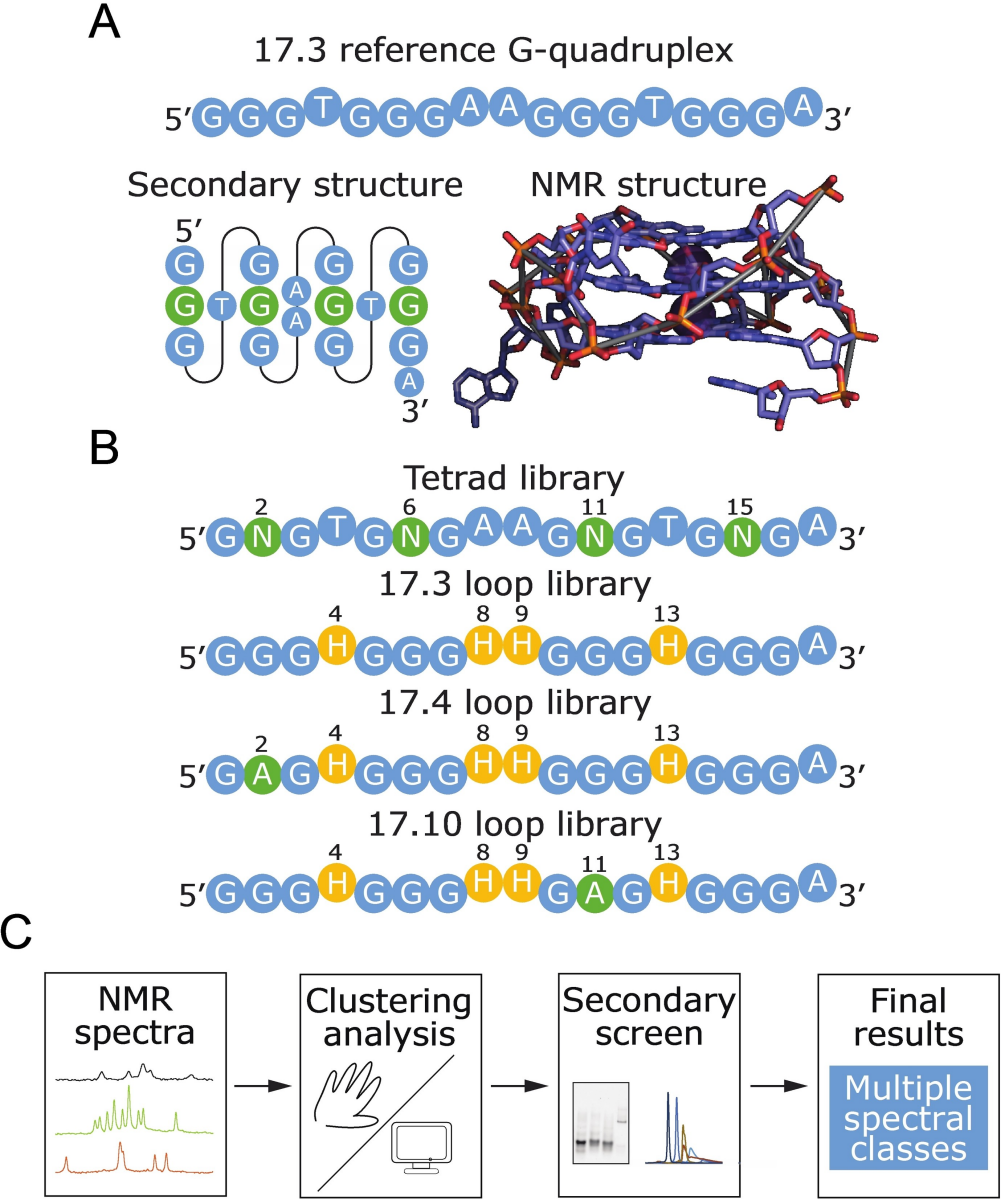
NMR screen of a G‐quadruplex library. (A) Primary sequence, secondary structure, and NMR structure of 17.3 (the reference sequence used as a starting point for the library). This forms a monomeric G‐quadruplex with three tetrads.^[19]^ The middle tetrad, highlighted in green, is formed by nucleotides 2, 6, 11, and 15, and is referred to as “the central tetrad of the reference G‐quadruplex” throughout this manuscript. Note that these positions do not necessarily form a tetrad in other sequences in the library. (B) Library design. Positions that can differ from 17.3 are shown in yellow or green. The tetrad library contains each of the 256 possible variants of the central tetrad in the monomeric reference G‐quadruplex. The 17.3 loop library contains each of the 81 possible loop variants (H=A, C, or T, but not G) in the background of the monomeric reference G‐quadruplex. The 17.4 loop library contains each of the 81 possible loop variants in the background of a representative dimeric G‐quadruplex containing a G to A mutation at position 2 in the central tetrad of the reference G‐quadruplex. The 17.10 loop library contains each of the 81 possible loop variants in the background of a representative tetrameric G‐quadruplex containing a G to A mutation at position 11 in the central tetrad of the reference G‐quadruplex. Note that “central tetrad” and “loop” refer to the structure of the reference sequence, but not necessarily to the structure of any other sequence. (C) Workflow. The first step of the NMR screen was to measure ^1^H NMR spectra of all sequences in the library at two different timepoints. The figure shows examples of signals in the G‐quadruplex region of ^1^H NMR spectra. The second step was to sort spectra into classes by manual inspection and computer clustering. The third step was to select at least one representative sequence from each major class and to perform a secondary screen consisting of native PAGE analysis over a range of conditions, ion exchange chromatography, and both native PAGE and ^1^H NMR time courses of slow folding sequences. The final step was to process the data, which yielded the results presented here.

In this study NMR was used to screen a 496‐member library of variants of a monomeric reference G‐quadruplex with a known three‐dimensional structure (Figure 1a–b). The first step in this workflow was to measure ^1^H NMR spectra of all sequences in the library at two different timepoints. Sequences were then sorted into classes using both visual inspection and computer clustering. Representative sequences from each major class were characterized in more detail in a secondary screen. Finally, results obtained using both methods of sorting were further analyzed. This workflow is summarized in Figure 1c.

### Sequences with no Mutations in the Central Tetrad

**Figure 2:**
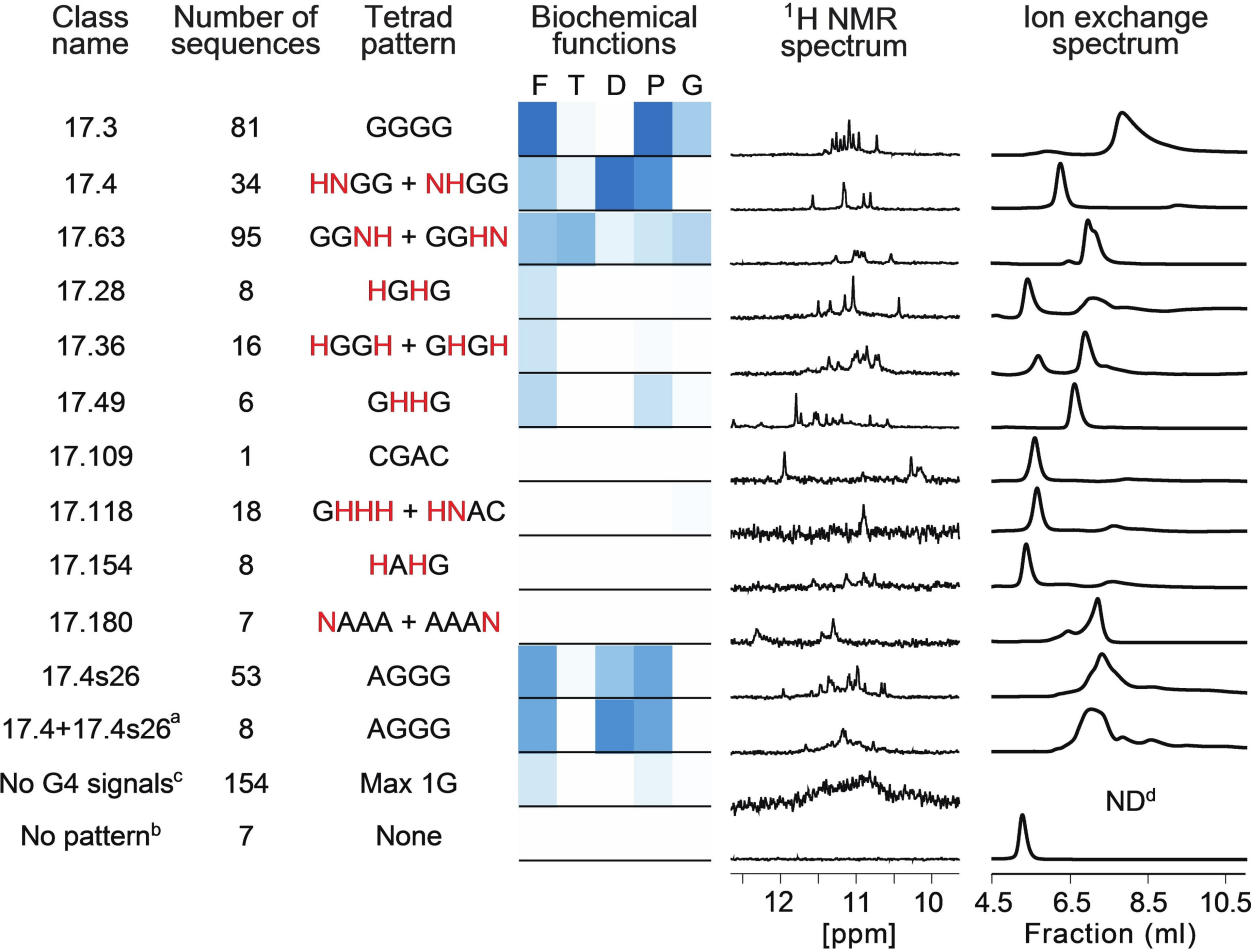
List of all major spectral classes identified in this study. First column=class name. Second column=number of sequences in the class. Third column=main sequence pattern of positions 2, 6, 11, and 15 (which form the central tetrad of the reference G‐quadruplex 17.3) in sequences in the class. Fourth column=graphical representation of five previously measured biochemical activities among sequences in the class. F=intrinsic fluorescence; T=the ability to form tetramers; D=the ability to form dimers; P=the ability to promote a model peroxidase reaction; G=the ability to bind GTP.[^15^, ^19^, ^20^, ^21^, ^22^] Fifth column=^1^H NMR spectrum of a representative sequence in the class. Note that intensities in different spectra are not comparable, as not all spectra were measured with default parameters and are displayed with different scales. Sixth column=ion exchange chromatogram of a representative sequence in the class. Note that intensities in different traces are not comparable. a Class 17.4+17.4 s26 contains sequences with ^1^H NMR spectra that share characteristics with those of both Class 17.4 and Class 17.4 s26. b Class “no pattern” is made up of sequences with ^1^H NMR spectra containing signals with no clear pattern in the G‐quadruplex part of spectrum. c Class “no G4 signals” contains all sequences with no signals in the G‐quadruplex part of the ^1^H NMR spectrum. d Class “no pattern” is not a proper class, so a representative sequence could not be chosen for the secondary screen.

**Figure 3:**
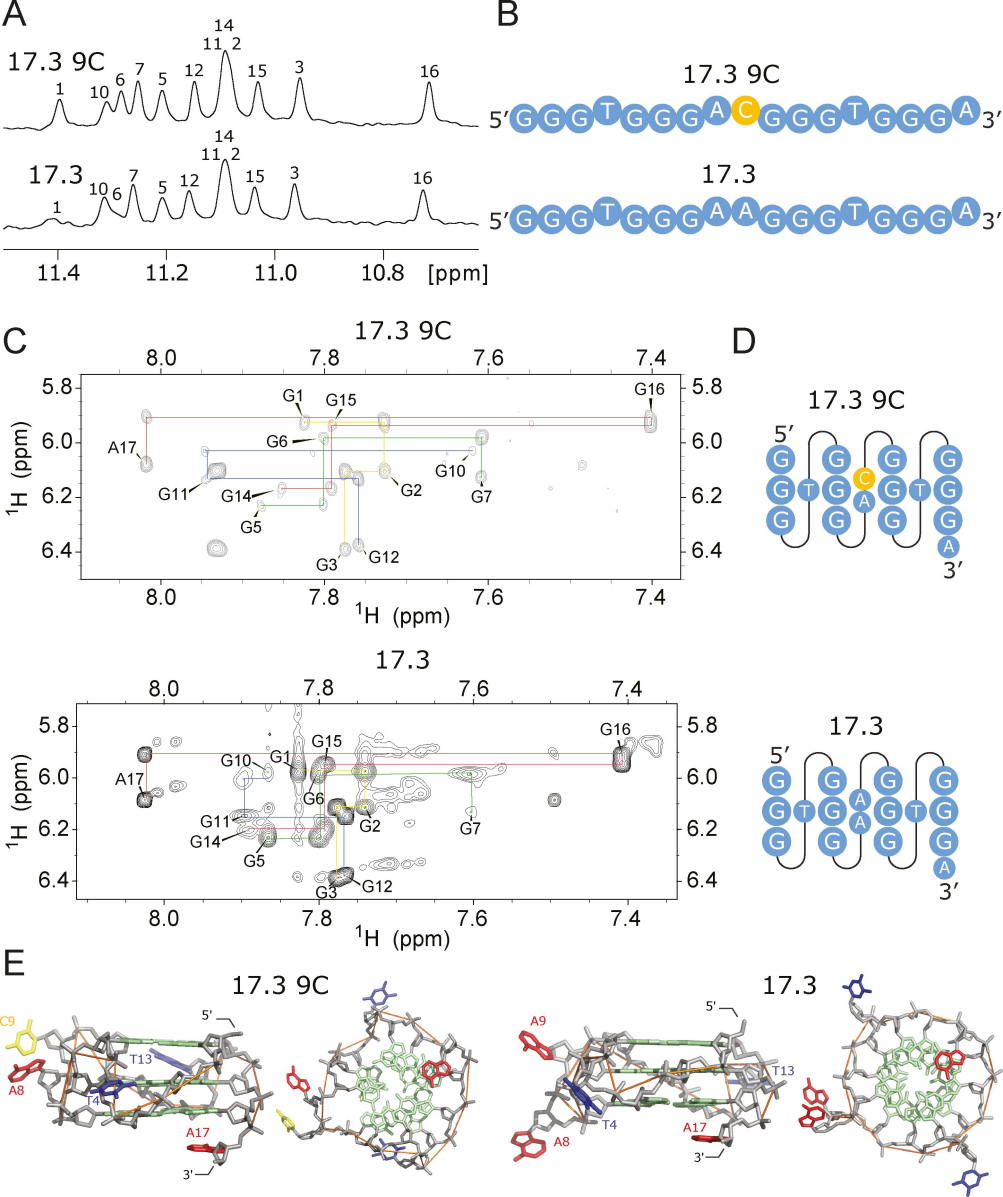
Similar ^1^H NMR spectra imply similar structures. (A) ^1^H NMR spectra of sequences 17.3 9 C and 17.3 with marked assignment of imino protons. The assignment for 17.3 was determined previously in^[19]^ and the assignment for 17.3 9 C was derived from comparison with 17.3. (B) Primary sequences of 17.3 9 C and 17.3, with the point mutation in 17.3 9 C shown in yellow. (C) Sequential walk in NOESY spectra of sequences 17.3 9 C and 17.3.^[19]^ The only hydrogen with a significantly shifted resonance is H8 in G10, which is a nucleotide next to the point mutation. The degree of similarity of the NOESY spectra of sequences 17.3 9 C and 17.3 confirms that they have similar structures. (D) Secondary structures of sequences 17.3 9 C and 17.3. (E) Comparison of high‐resolution structures of sequences 17.3 9 C and 17.3.

In a previous study we determined the high‐resolution structure of a monomeric G‐quadruplex from the library named 17.3.^[19]^ The structure of 17.3 contains three stacked tetrads connected by short propeller‐type loops (Figure 1a). The ^1^H NMR spectra of the 80 other sequences in the library with an unmutated central tetrad are similar to (and in some cases almost indistinguishable from) the spectrum of 17.3 (Figure 2 and Figure S13). We named this group of sequences Class 17.3 and suggest that they form structures similar to that of 17.3 (but also see below). Consistent with this idea, the structure of a representative member of this class was determined, and it was virtually identical to that of 17.3 (Figure 3 and Supplementary Information Section SI structure). Also consistent with this idea, the ^1^H NMR spectrum of sequence PEA1–20 in^[36]^ (which is identical to sequence 17.3 s64 in our library except that it lacks the 3′ A) is almost identical to those of the sequences in Class 17.3, and its high‐resolution structure is almost identical to that of 17.3. Sequences in Class 17.3 have similar functional properties: they exhibit the highest average fluorescence and peroxidase activities of any sequence class in the library, and they also bind GTP efficiently (Figure 2). Interestingly, several of these sequences form tetramers to a limited extent on native gels, indicating that mutations in loops of a monomeric G‐quadruplex can induce multimerization. Of the nine sequences in Class 17.3 that form tetramers, eight contain the sequence AAHH at positions 4, 8, 9, and 13 (the positions that form loops in the reference G‐quadruplex). However, the ^1^H NMR spectra of these tetramer‐forming sequences are similar to those of sequences in Class 17.3 that only form monomers. One possible explanation is that variants containing AAHH loops form a mix of tetramers and 17.3‐like monomers. This interpretation is supported by ion exchange chromatography: for example, the spectra of sequences 17.3 s53 and 17.3 s19 contain additional signals compared to members of this class that do not form tetramers (Figure S13). On the other hand, the G‐quadruplex parts of the spectra of these sequences do not contain extra signals compared to those of sequences which form only monomers. This could be due to spectral overlap in ^1^H NMR spectra and/or lower signal to noise ratios which are characteristic of the NMR spectra of multimers compared to those of monomers. Computer clustering suggests that class 17.3 contains four subclasses of sequences, which are defined based on small differences in ^1^H NMR spectra (see the SI for additional details). Taken together, these results suggest that at least 16 % of the sequences in the library form structures similar to that of the monomeric reference G‐quadruplex 17.3. They also show that classes determined based on analysis of ^1^H NMR spectra can correspond to those determined based on independent analyses of functional properties.

### Sequences with one Mutation In the Central Tetrad

**Figure 4:**
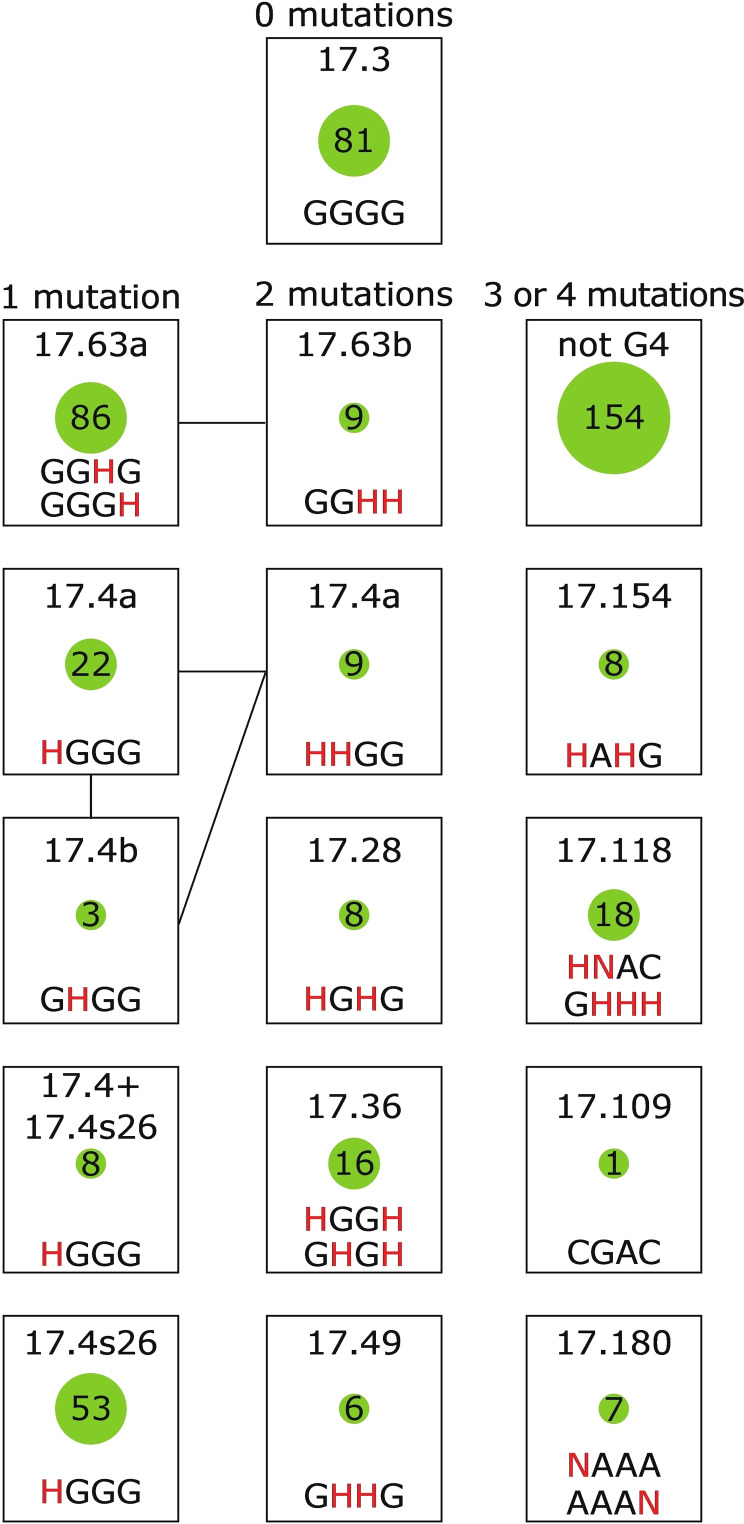
Mind map showing major and some minor classes identified in this study that can be differentiated based on tetrad sequence. Each rectangle indicates a different class. The text above rectangles indicates the number of mutations in the nucleotides that form the central tetrad of the reference G‐quadruplex (corresponding to nucleotides 2, 6, 11, and 15). Sequences inside each rectangle indicate the nucleotides that occur at positions that form the central tetrad in members of the class. Positions at which more than one nucleotide can occur are shown in red. N=A, C, G, or T; H=A, C, or T. Numbers inside green circles indicate the number of sequences in the corresponding class. Lines connecting rectangles connect minor classes in the same major class. Subclasses which cannot be differentiated by tetrad sequence are shown in the mind maps of loop libraries in Figure 5. The class “no clear pattern” was omitted since it is not a proper class of sequences with similar properties.

**Figure 5:**
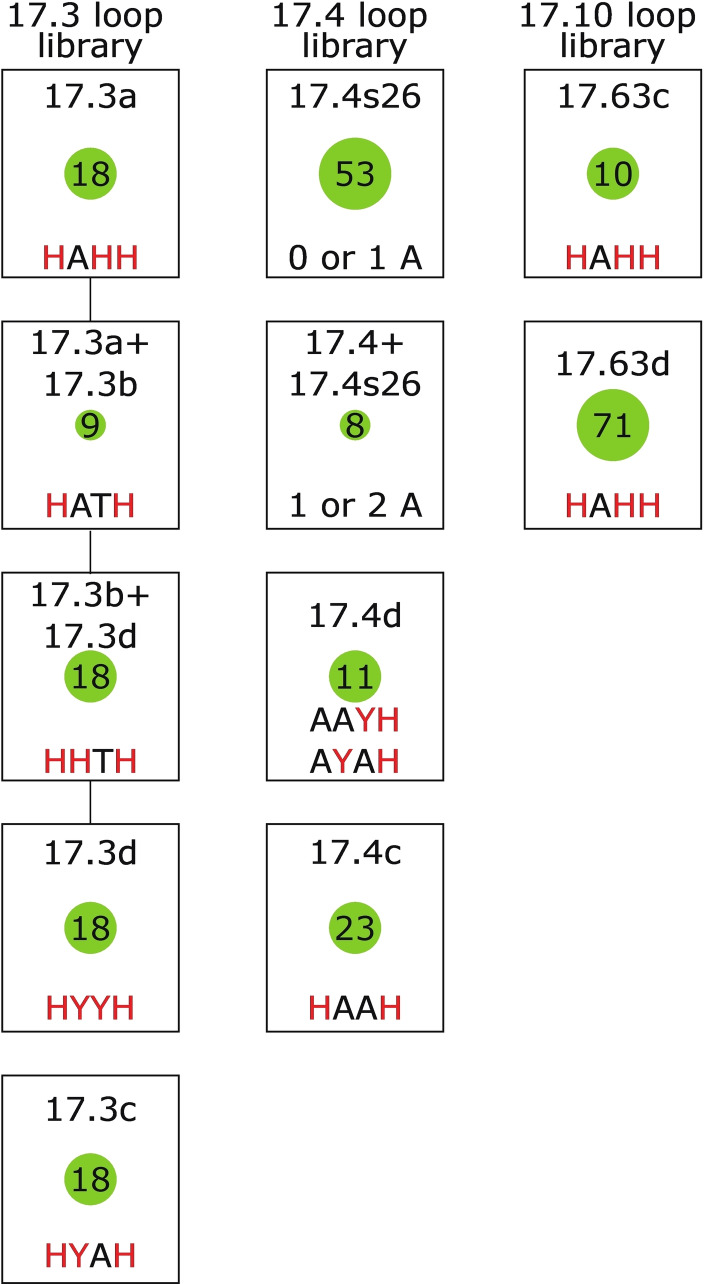
Mind map showing sequence classes in loop libraries. Each rectangle indicates a different class. Sequences inside each rectangle indicate the nucleotides at positions that form loops in the reference G‐quadruplex (corresponding to nucleotides 4, 8, 9, and 13) in members of the class. “0 or 1 A” and “1 or 2 A” indicates the total number of adenosines at positions 4, 8, and 9. Positions at which more than one nucleotide can occur are shown in red. H=A, C, or T; Y=C or T. Numbers inside green circles indicate the number of sequences in each class. Lines connecting rectangles indicate overlap between classes.

The commonly used G‐quadruplex consensus sequence G_3+_N_1–7_ G_3+_N_1–7_G_3+_N_1–7_G_3+_ does not include sequences that contain mutations in tetrads. However, such sequences often form G‐quadruplexes in the context of our library (typically due to multimerization). Previous analysis by native PAGE revealed two structural classes of multimers: dimers and tetramers. However, the results of our NMR screen suggest that the structural landscape of the library is more complex. Spectral analysis suggests that the 172 sequences in the library with a single mutation in the central tetrad of the reference G‐quadruplex can be grouped into four different major classes. One (called Class 17.63) contains sequences with mutations at positions 11 or 15, and largely corresponds to library members shown to form tetramers in previous studies (Figure 4 and Figure 5). In contrast, sequences that contain mutations at positions 2 or 6 in the central tetrad of the reference G‐quadruplex and were previously shown to form dimers by native PAGE form three major classes: Class 17.4, Class 17.4 s26, and Class 17.4+17.4 s26 (Figure 4 and Figure 5). ^1^H NMR spectra of sequences in Class 17.4 contain a characteristic pattern of up to six peaks, and signal to noise ratios are typically higher than those of sequences in Class 17.4 s26 (compare Figures S18 and S35). Spectra of sequences in Class 17.4 s26 contain a significantly higher number of signals than those in Class 17.4, sometimes even more than 11 (the number of guanosines in sequences from this class; Figure S35), which implies that at least some sequences from Class 17.4 s26 can form more than one structure. NMR spectra of sequences from Class 17.4+17.4 s26 contain signals characteristic of both Classes 17.4 and Class 17.4 s26 (Figure S36). Sequences from Class 17.4 s26 usually contain signals in a wider region of the spectrum (1.5 ppm) than those of sequences in Class 17.4 (0.9 ppm). Ion exchange chromatography spectra are also usually more complicated for sequences in Classes 17.4 s26 and 17.4+17.4 s26 than for those in Class 17.4, providing additional evidence that these classes are different. Taken together, these results show how NMR screening can be used to obtain new structural information about a library. They also suggest that sequences with one mutation in the central tetrad of the reference G‐quadruplex can adopt several different types of structures.

### Sequences with Two Mutations in the Central Tetrad

Sequences containing two mutations in the central tetrad of the reference G‐quadruplex are even less consistent with commonly used G‐quadruplex consensus sequences than those containing a single mutation. Despite this, our results indicate that such sequences can also form G‐quadruplexes. As is the case for sequences containing a single mutation in the central tetrad, the classes into which these sequences belong are related to (but not completely determined by) the positions of the mutations in this tetrad. Five different classes were identified based on manual inspection of NMR spectra (Figure 2 and Figure 4). Two of these classes, 17.4 and 17.63, also occur among sequences that contain a single mutation in the central tetrad and are described above. Three additional classes were also observed. These are Class 17.36 (which consists of 16 library members with the sequence GHGH, HGGH, or TGGT at the positions that form the central tetrad of the reference G‐quadruplex), Class 17.28 (which consists of eight library members with the sequence HGGH in the central tetrad), and Class 17.49 (which consists of six library members with the sequence GHHG in the central tetrad). The spectra of sequences in this latter class are strikingly similar to those of sequences in Class 17.3 (Figure S3), suggesting that they form structures with some similarities to that of 17.3 (Figure 1a). Several other sequences could not be easily classified based on their spectra. Some of these classes contain sequences that appear to adopt multiple conformations. For example, the spectra of sequences in Class 17.28 contain up to 11 signals in the G‐ quadruplex part of the spectrum (Figure S28). Since these sequences contain 10 guanosines, it is likely that they adopt multiple conformations. Another interesting feature of these sequences is that some only form G‐quadruplexes after long incubations (described in more detail in the section “Slow folding sequences”). These results provide additional information about previously unidentified classes of sequences in our library. These also suggest that library members that contain two mutations in the central tetrad of the reference G‐quadruplex are even more structurally diverse than those that contain zero or one mutation in this tetrad.

### Sequences with Three or Four Mutations in the Central Tetrad

Previous analysis showed that sequences with three or four mutations in the central tetrad of the reference G‐quadruplex do not have functions associated with G‐quadruplexes. The results of our NMR screen are consistent with these results, and suggest that such sequences do not form G‐quadruplex structures. Indeed, the spectra of many (154 out of 189 such sequences) do not contain any signals in the G‐quadruplex part of the spectrum. However, other sequences contain up to four signals in this region. Furthermore, manual analysis of spectra revealed that, in some cases, such sequences form classes with similar spectra and well‐defined mutational signatures. For example, Class 17.180 contains all seven sequences in the library with an NAAA or AAAN sequence at positions 2, 6, 11, and 15 (the positions that form the central tetrad of the reference G‐quadruplex), while Class 17.154 contains six library members with the sequence HAHG at these positions. In addition to having distinctive NMR spectra, ion exchange chromatography spectra of representative sequences in these classes tend to differ from those that do not contain signals in the G‐quadruplex part of the spectrum (compare Figure S31, Figure S32, Figure S33, and Figure S34 to Figure S38). These results raise the possibility that such sequences form well‐defined structures containing hydrogen bonds similar to the ones in G‐quadruplexes, although it is unlikely that these structures are G‐quadruplexes.

### Correspondence Between Structural and Functional Classes

**Figure 6:**
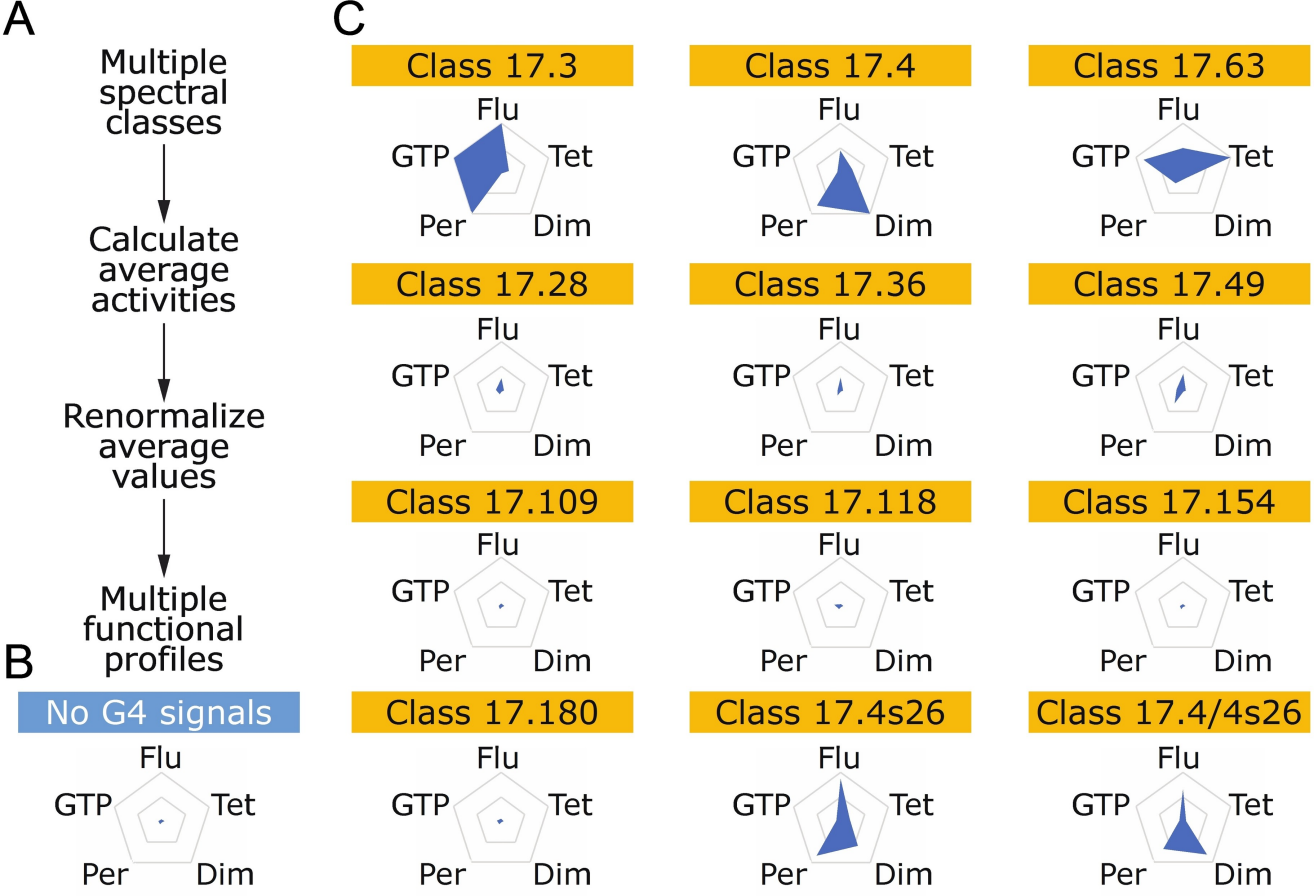
Sequences in different spectral classes can have distinct functional properties. (A) Workflow of analysis. (B) Radar plot showing the activity profile of sequences in the library whose NMR spectra do not contain signals consistent with a G‐quadruplex structure. (C) Radar plots showing activity profiles of the twelve major spectral classes in the library with spectra consistent with G‐quadruplex structures. Flu=intrinsic fluorescence; Tet=the ability to form tetramers; Dim=the ability to form dimers; Per=the ability to promote a model peroxidase reaction; GTP=the ability to bind GTP. Activity profiles were determined using data from previous studies.[^15^, ^19^, ^20^, ^21^, ^22^] and all data were linearly renormalized to a scale from 0 to 100 so that 100 is a maximum average activity among all major classes for each activity.

When taken together, this analysis suggests that our library contains multiple classes of G‐quadruplex structures (including classes not previously identified based on analysis using low resolution methods such as native PAGE). To determine the extent to which these groupings correspond to sequences with unique functional properties, we determined the average activity of sequences in each class with respect to the ability to bind GTP, promote a model peroxidase reaction, form dimers, form tetramers, and to generate intrinsic fluorescence using data from previous studies[^15^, ^19^, ^20^, ^21^, ^22^] (Figure 1). After renormalization, activity profiles were visualized using radar plots (Figure 6). This analysis demonstrated that sequences in different spectral classes often have distinct functional properties. For example, the average GTP‐binding activity of the sequences in Class 17.63 is approximately 12‐fold higher than that of the sequences in Class 17.4, whereas the average peroxidase activity is approximately 3‐fold lower (Figure 6). It also revealed that six classes stand out by having two or more activities with average values significantly above background: 17.3, 17.4, 17.4 s26, 17.4+17.4 s26, 17.63, and to a lesser extent 17.49 (Figure 6). Classes 17.3, 17.63, and 17.49 differ significantly from all other classes, while 17.4, 17.4 s26 and 17.4+17.4 s26 are similar to one another but different from other classes. The relative standard deviation of the average value of a given biochemical activity is usually significantly smaller within a class than it is for a randomly chosen group of sequences of the same size (Figure S11), indicating that the functional properties of sequences in a class are relatively uniform. An exception, however, is the GTP binding activity of sequences in Class 17.63 (and most of its subclasses) and Class 17.118. This highlights the difference between GTP binding and other functional properties of the library. These other functions are influenced mostly by the tetrad sequence pattern (which is closely connected with spectral class). On the other hand, GTP binding is also strongly influenced by loop sequence. One possible explanation is that GTP interacts with specific nucleotides at specific positions, whereas other functional properties are probably a function of overall structure. Taken together, these results indicate that individual classes contain sequences with similar biochemical properties. They also demonstrate that classes created according to NMR spectra are often functionally distinct from one another.

### Slow Folding Sequences

**Figure 7:**
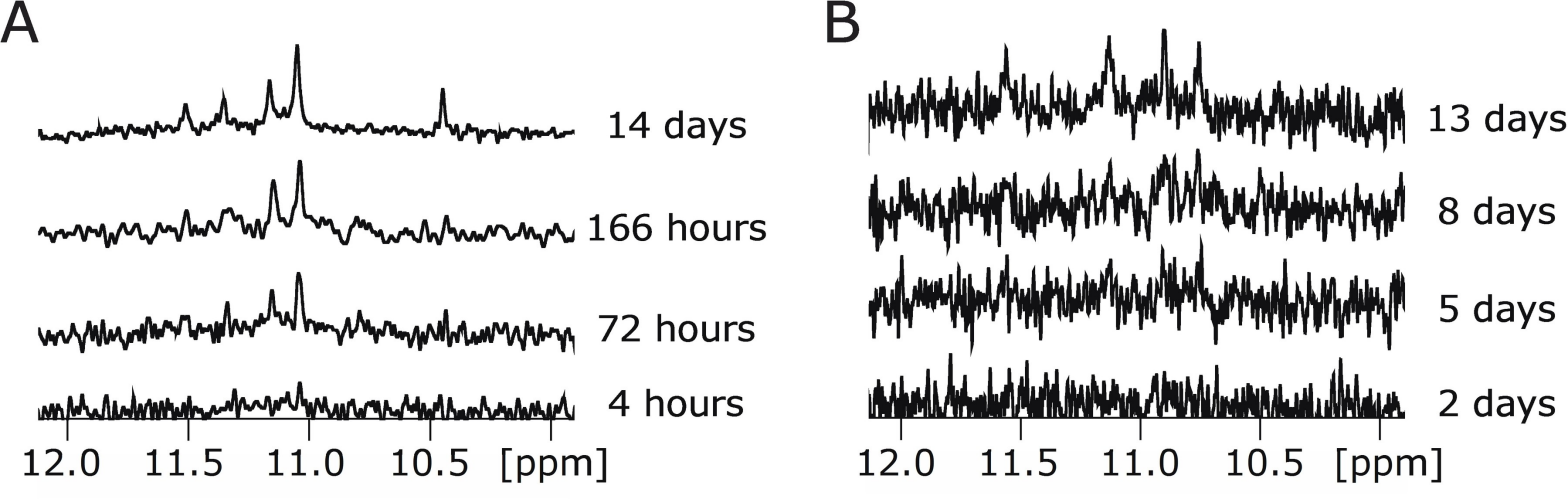
G‐quadruplexes with slow folding rates. (A) ^1^H NMR spectra of sequence 17.28 measured 4 hours, 72 hours, 166 hours, and 14 days after preparation. The spectrum determined after 14 days was measured with 2048 scans and is displayed with scale 0.25, while all other spectra were measured with 512 scans. (B) ^1^H NMR spectra of sequence 17.154 measured 2 days, 5 days, 8 days, and 13 days after preparation. All four spectra were measured with 1024 scans.

In previous functional screens of this library, sequences were characterized after a short (30 minute) incubation. To investigate the possibility that some sequences in this library (particularly those that do not match the G‐quadruplex consensus sequence) might require longer folding times, a second set of NMR spectra was measured after incubating sequences in the library for two months. This screen revealed that some sequences in the library require days or weeks to fold (Figure 7, S4, and S5). Class 17.28 contains two such sequences, called 17.28 and 17.29. NMR experiments indicate that 17.29 is fully folded in two days (Figure S5), while 17.28 requires two weeks to fold (Figure 7 and S4). Independent analysis using native gels indicates that 17.28 dimerizes over the span of 10 weeks (Figure S4). A second class with slow folding sequences is Class 17.154. A sequence in this class (called 17.154) required 13 days to fully fold based on NMR experiments. In comparison, native gels suggest that this sequence folds over the span of six weeks and forms a mix of monomeric, dimeric, tetrameric, and even larger structures (Figure S4). These results suggest that certain sequences which are not consistent with the G‐quadruplex consensus sequence can nevertheless form G‐quadruplex structures if given sufficient time to fold.

## Conclusions

In several previous studies we characterized the functional properties of each of the sequences in a 496‐member library of variants of a monomeric reference G‐quadruplex.[^15^, ^19^, ^20^, ^21^, ^22^] Here we used ^1^H NMR to obtain a broad overview of the structural features of this library. One unique aspect of our approach is that this library is more than an order of magnitude larger than those typically used to study G‐quadruplexes (for example^[33]^). Another is that this dataset made it possible to compare classes identified by clustering of NMR spectra to those generated using independent methods. One important conclusion is that a wide range of sequences that differ from the commonly used G‐quadruplex consensus sequence G_3+_N_1–7_G_3+_N_1–7_G_3+_N_1–7_G_3+_ can nevertheless form G‐quadruplex structures. This emerging idea is also supported by a number of other recent studies. For example, it has been shown that tetrads in G‐quadruplexes can contain mutations and/or bulges,[^4^, ^37^, ^38^] and structures with noncanonical tetrads have also been reported.[^39^, ^40^] Loops can also be significantly longer than the seven nucleotides allowed in the standard consensus sequence, including in some cases up to 30 nucleotides.^[41]^ And studies using a G‐quadruplex‐specific antibody have revealed that the human genome contains hundreds of thousands more G‐quadruplexes than were initially predicted by a consensus model.^[42]^ These examples appear to mostly correspond to monomers with unusual structural elements such as noncanonical tetrads. Our study highlights two additional mechanisms by which sequences that differ from the consensus model can form G‐quadruplexes. One of these is multimerization. By this mechanism, defects in a sequence (such as mutations in tetrads) can sometimes be compensated for by another copy of the molecule. A second mechanism is slow folding. Previous studies have shown that chaperon proteins can promote G‐quadruplex formation (for example[^43^, ^44^, ^45^]). Our results show that at least some sequences that differ from the G‐quadruplex consensus can also form G‐quadruplexes in the absence of chaperons when given sufficient time to fold (Figure 7), although they do not address whether such structures are biologically relevant.

While efforts to expand the definition of G‐quadruplexes have received increased attention in recent years, attempts to make this definition more specific are less explored. A major goal of such efforts is to resolve the discrepancy between the large number of structures and functions of G‐quadruplexes and the single standard G‐quadruplex consensus sequence. Previous studies from our group showed that the sequence requirements of G‐quadruplexes with different functions in the library analyzed here are overlapping (sequences in the library often have multiple activities) but distinct (the subset of sequences with one activity never perfectly overlaps with the subset of sequences with a second activity).[^15^, ^19^, ^20^, ^21^, ^22^] They also suggested that the library contains at least three types of G‐quadruplexes: monomers, dimers, and tetramers. The NMR screen described here revealed evidence for the existence of additional structural classes, including some not detected in previous studies. These observations highlight the remarkable structural diversity of our library, and in a more general sense of G‐quadruplex structures.

**Figure 8:**
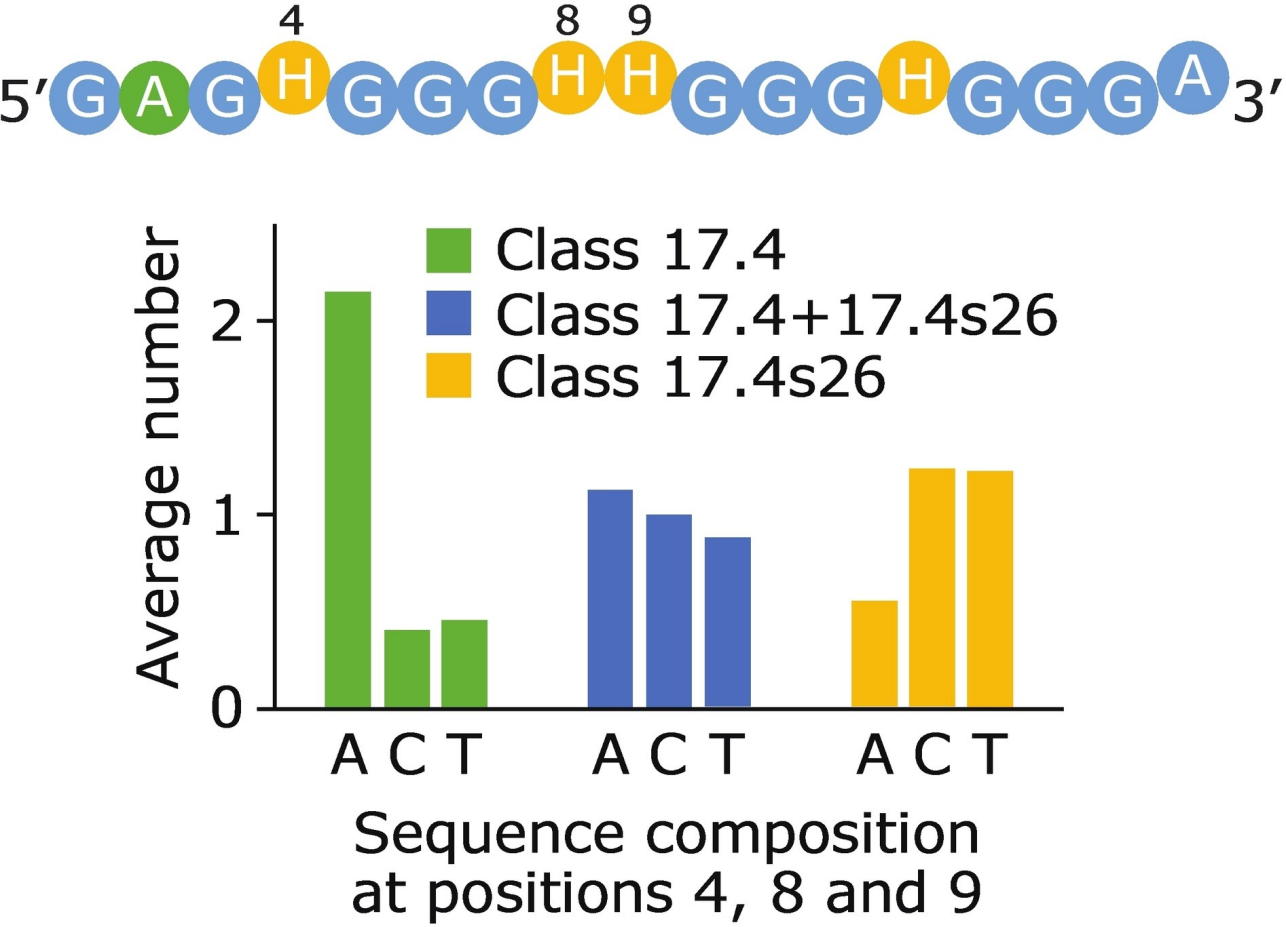
Number of adenosines at positions 4, 8, and 9 are correlated with spectral class among sequences in the 17.4 loop library. Above: sequence of the 17.4 loop library. Positions that can differ from 17.3 are shown in green or yellow, and positions 4, 8, and 9 are numbered. Below: average number of occurrences of given nucleotide at loop positions 4, 8, and 9 among sequences in different major classes in the 17.4 loop library.

Another goal of this study was to better understand the complex relationship between sequence and structure. Somewhat surprisingly, many of the structural classes identified in our library can be described using relatively simple mutational signatures. This suggests that it could be possible to develop multiple consensus motifs for G‐quadruplexes, each corresponding to sequences with distinct structural properties. The main parameter that determines the spectral class into which a sequence belongs is the pattern of mutations in the positions that form the central tetrad in the monomeric reference G‐quadruplex. Sequences in which either the first two positions in the tetrad (positions 2 and 6) or the last two positions in the central tetrad (positions 11 and 15) are not mutated follow three rules (Figure 4). If both the first and second halves of the tetrad are unmutated, the sequence will form a monomeric G‐quadruplex and belong to Class 17.3. If only the first half of the tetrad is unmutated, the sequence will form a tetrameric G‐quadruplex and belong to Class 17.63. And if only the second half of the tetrad is unmutated, the sequence will typically form a dimeric G‐quadruplex and belong to Class 17.4 (although we note that this rule does not hold for all sequences in the library, such as those in Class 17.4 s26). These rules suggest that only part of each sequence (guanosines 1–3 and 5–7 in tetramers and guanosines 10–12 and 14–16 in dimers) is involved in tetrad formation in multimeric G‐quadruplexes. In support of this model, we consistently observe fewer G‐quadruplex signals in the NMR spectra of sequences that form dimeric and tetrameric G‐quadruplexes than in those that form monomeric structures. While less important than mutations in tetrads, mutations in loops can also play roles in determining the class to which a sequence belongs. For example, the pattern of mutations in loop positions determines the distribution of sequences in Class 17.3 into subclasses with similar NMR spectra (Figure 5). It can also determine the distribution of sequences in classes with significantly different NMR spectra, as is the case for sequences in the 17.4 loop library (Figure 5). When we consider the three major classes of sequences in this loop library, we can see that the number of adenosines at the first three loop positions (positions 4, 8, and 9) differs for each class. Specifically, sequences in Class 17.4 s26 contain zero or one adenosines at positions 4, 8, and 9, sequences in Class 17.4 contain two or three adenosines, and sequences in Class 17.4+17.4 s26 (which have characteristics of both of these classes) contain one or two adenosines (Figure 8 and S12). If adenosines at these positions are destabilizing (as has been observed for loop adenosines in at least some G‐quadruplexes),[^41^, ^46^, ^47^] this could rationalize some of the spectral details of these three classes. In this view, sequences in Class 17.4 s26 form an unstable higher order structure that is destabilized by adenosines at positions 4, 8, and 9. This explains why some spectral traces of this structure can be seen in variants that contain one or two adenosines, but not in sequences in Class 17.4, which contain two or three adenosines.

A final point concerns the relationship between structure and function. Sequences in the different spectral classes identified in this study typically exhibit characteristic patterns of biochemical functions (Figure 6). These functions are also distributed less randomly in spectral classes than in the dataset as a whole. These observations suggest that it could be possible to use NMR spectra to predict if a G‐quadruplex forming sequence will exhibit a particular biochemical activity. This is already possible to some extent among the sequences in this library. For example, the spectra of all sequences in the library with at least one biochemical activity contain multiple G‐quadruplex signals. It is possible that this approach could also be used to identify sequence elements important for specific biochemical functions. For example, virtually all of the sequences in the library that bind GTP efficiently are in Class 17.3 or Class 17.63. It is possible that the ability of a G‐quadruplex to bind GTP is connected to a feature shared by sequences in these classes, but not by sequences in other classes. One such feature is the presence of guanosines at positions 2 and 6 (which form part of the central tetrad in the reference G‐quadruplex).

In conclusion, we used ^1^H NMR to investigate the structural properties of a 496‐member G‐quadruplex library. We discovered that the position of mutations in the central tetrad of the monomeric G‐quadruplex used as the reference sequence for this library almost entirely determines the distribution of library members into classes with similar spectra. We also found that the structural classes identified using this approach have different patterns of biochemical activities. Our results provide additional evidence that the commonly used G‐quadruplex consensus sequence is too general, and that it also fails to identify many sequences with the ability to form G‐quadruplex structures. They also provide a systematic and unprecedented view of the structural features of a large G‐quadruplex library.

## Experimental Section

### NMR Screen

#### Purification

Desalted DNA oligonucleotides were purchased from Generi Biotech. Additional purification was necessary, and achieved by repeated concentration and dilution using Amicon Ultra‐0.5 Centrifugal filter devices with a cut off of 3 kDa. This is smaller than the molecular weight of the DNA sequences used in this study (around 5.54 kDa) and considerably larger than the molecular weight of impurities contained in desalted oligonucleotides (mainly mono‐ and dinucleotides). To remove ethanol from membranes, filter devices were spun six times in a centrifuge at 14,000 g for 15 minutes with 500 μl of Milli‐Q water. Desalted DNA oligonucleotides were dissolved in Milli‐Q water at 200 μM, and 80 μl was transferred to an Amicon filter device. This was spun in a centrifuge twice at 14,000 g for 20 minutes. After each spin, the filtrate was discarded, and 450 μl of Milli‐Q water was added. Next, filter units were spun at 14,000 g for 30 minutes. Samples were extracted using a pipet tip and transferred to a PCR strip. The pipet tip was used to measure the volume of each sample, and an appropriate volume of Milli‐Q water was added with a target volume of 80 μl and a target concentration of 200 μM. Purified samples were stored in a freezer at −20 °C.

### Folding and Measurement of ^1^H NMR Spectra

Purified samples were thawed for 30 minutes at room temperature, heated at 65 °C for 5 minutes, and cooled at room temperature for 5 minutes. We next added 40 μl of 4× buffer (800 mM KCl, 4 mM MgCl_2_, 80 mM Tris, pH 7.1, and a trace amount of DSS as a standard for NMR measurements), 24 μl of Milli‐Q water, and 16 μl of D_2_O. Final concentrations were 100 μM DNA, 200 mM KCl, 1 mM MgCl_2_, 20 mM Tris, pH 7.1, and 10 % D_2_O in a volume of 160 μl. Samples were incubated for 30 minutes at room temperature and transferred to a temperature cycler. They were then heated at 97 °C for 30 minutes, 95 °C for one minute, and cooled at 1 °C per minute until a temperature of 25 °C was reached. Samples were next transferred to 3 mm NMR cuvettes and ^1^H NMR spectra were measured on a Bruker Avance III HD 850 MHz spectrometer with sample changer after preparation (within three days), incubated for two months at room temperature and again measured. Spectra were acquired using the pulse program zgesgp at a temperature of 298 K using 256 scans, 16 dummy scans, and a pre‐scan delay 20 μs if not stated otherwise. This corresponded to nine minutes per sample (approximately 100 hours of experimental time for the whole library). Some measurements were repeated with a larger number of scans and/or after various incubations times, the details of which are provided below. During our measurements, some samples slowly decomposed for unknown reasons, which in some cases prevented us from obtaining reliable spectra after weeks on the bench. These samples were prepared again at a lower concentrations and experiments were repeated. Default parameters for samples and ^1^H NMR spectra are first preparation, DNA concentration 100 μM, spectrum measured right after preparation (within three days), and 256 scans. When other parameters were used, this is mentioned in the legend of the figure in which the spectrum is shown.

### ^1^H NMR Spectra of Slow Folding Sequences

Samples of the sequences 17.28 and 17.154 (which were used to study the time dependence of ^1^H NMR spectra) were prepared from desalted DNA oligonucleotides purchased from Sigma. All other details of sample preparations and measurements were identical to those used in the NMR screen described above.

### Ion Exchange Chromatography

Desalted DNA oligonucleotides were purchased from Sigma. DNA was resuspended in Milli‐Q water, heated at 65 °C for 5 minutes, cooled at room temperature for 5 minutes, and folded by adding buffer. Final concentrations were 10 μM DNA, 200 mM KCl, 1 mM MgCl_2_ and 20 mM Tris, pH 7.1 in volume of 800 μl. Samples were then analyzed by MonoQ ‐ ion‐exchange chromatography (1 ml volume, GE Healthcare) using a linear gradient from 0.2 to 1 M KCl. All samples were prepared and analyzed at once except for a sample of sequence 17.8. The results of ion exchange chromatography separations were analyzed by comparing the results to those obtained from five model sequences: 17.3 was used as a model monomer, 17.4 was used as a model dimer, 17.10 and 17.63 were used as model tetramers, and 17.201 was used as a negative control sequence that does not form G‐quadruplex (Figure S2, Table 1).

**Table 1:** List of peak types in ion exchange chromatography spectra.

Peak name	From (ml)	To (ml)
Not G4 Monomeric Dimeric Tetrameric Many mutations	5.1 7.7 6.1 6.8 5.55	5.4 9.0 6.7 7.4 5.7

### Native Gels

#### Secondary Screen

All sequences chosen for the secondary screen were analyzed on native gels using four different conditions: biochemical conditions (the same conditions as used in^[20]^), biochemical conditions with annealing, NMR conditions with low concentration, and NMR conditions. It was not possible to perform the NMR screen with samples prepared exactly as in previous studies because the DNA concentration used previously (10 μM) was too low to perform NMR experiments. We instead used a higher DNA concentration and added an annealing step because it improved NMR spectra (particularly those of tetrameric G‐quadruplexes). Performing these experiments over a range of conditions helped us to better understand the effects of different variables on the results. Desalted DNA oligonucleotides were purchased from Sigma. Samples were purified as described above and were thawed at room temperature. For samples analyzed using biochemical conditions, we mixed 2.4 μl of 100 μM DNA oligonucleotide, 12 μl of 2× G4 buffer (400 mM KCl, 2 mM MgCl_2_, 40 mM HEPES, pH 7.1) and 9.6 μl of Milli‐Q water. Final concentrations were 10 μM DNA, 200 mM KCl, 1 mM MgCl_2_, and 20 mM HEPES, pH 7.1 in a volume of 24 μl. Samples were than heated at 65 °C for 5 minutes, and cooled at room temperature for 5 minutes. For samples analyzed using biochemical conditions with annealing, we mixed 2.4 μl of 100 μM DNA oligonucleotide, 12 μl of 2× G4 buffer (400 mM KCl, 2 mM MgCl_2_, 40 mM HEPES, pH 7.1) and 9.6 μl of Milli‐Q water. Final concentrations were 10 μM DNA, 200 mM KCl, 1 mM MgCl_2_, and 20 mM HEPES, pH 7.1 in a volume of 24 μl. Samples were then heated at 97 °C for 30 minutes, 95 °C for one minute, and cooled at a rate of 1 °C per minute until the sample reached 25 °C (the same annealing protocol was used for samples prepared for NMR measurements). For samples analyzed using NMR conditions with low concentration, we mixed 2.4 μl of 100 μM DNA oligonucleotide, 12 μl of 2× G4 NMR buffer (400 mM KCl, 2 mM MgCl_2_, 40 mM Tris pH 7.1) and 9.6 μl of Milli‐Q water. Final concentrations for samples analyzed using NMR conditions with low concentration were 10 μM DNA, 200 mM KCl, 1 mM MgCl_2_, and 20 mM Tris, pH 7.1 in a volume of 24 μl. Samples were then heated at 97 °C for 30 minutes, 95 °C for one minute, and cooled at a rate of 1 °C per minute until the sample reached 25 °C. For samples analyzed using NMR conditions, we mixed 2.4 μl of 1000 μM DNA oligonucleotide, 12 μl of 2× G4 NMR buffer (400 mM KCl, 2 mM MgCl_2_, 40 mM Tris pH 7.1) and 9.6 μl of Milli‐Q water. Final concentrations for samples analyzed using NMR conditions were: 100 μM DNA, 200 mM KCl, 1 mM MgCl_2_, and 20 mM Tris, pH 7.1 in a volume of 24 μl. Samples were then heated at 97 °C for 30 minutes, 95 °C for one minute, and cooled at a rate of 1 °C per minute until the sample reached 25 °C. All four types of samples were then incubated for 30 minutes at room temperature, and then 6 μl of 5× gel loading buffer (60 % w/v glycerol, 0.15 % w/v xylene cyanol and 0.15 % w/v bromophenol blue) was added to each sample. Material (500 ng) was analyzed by native PAGE using 10 % gels containing 5 mM KCl in both the gel and buffer. Gels were run for 30 min at 300 V. DNA was visualized by staining with GelRed using the protocol recommended by the manufacturer. Gels were scanned using Typhoon laser‐scanner and analyzed using ImageQuant software.

### Slow Folding

Representatives of two classes of slowly folding sequences were analyzed using native polyacrylamide gels. Time points were analyzed to follow the folding process. Desalted DNA oligonucleotides were purchased from Sigma. DNA samples were purified as described above and thawed at room temperature. DNA samples were then mixed with 4× NMR buffer (800 mM KCl, 4 mM MgCl_2_, 80 mM Tris pH 7.1). Final concentrations were 100 μM DNA in 200 mM KCl, 1 mM MgCl_2_, and 20 mM Tris‐HCl, pH 7.1. Samples were then heated at 97 °C for 30 minutes, 95 °C for one minute, and cooled at a rate of 1 °C per minute until the sample reached 25 °C. Samples were prepared by this protocol multiple times at different time points and then stored in a fridge at 4 °C for several weeks to monitor the slow folding process. Before analysis by native PAGE, samples were mixed with 6× native gel loading dye (60 % w/v glycerol, 0.15 % w/v xylene cyanol and 0.15 % w/v bromophenol blue) at once. Material (500 ng) was analyzed by native PAGE using 10 % gels that contained 5 mM KCl in both the gel and buffer. Gels were run for 30 min at 300 V. DNA was visualized by staining with GelRed using the protocol recommended by the manufacturer. Gels were scanned using Typhoon laser‐scanner and analyzed using ImageQuant software.

### Names of Classes

The first part of the name of almost all classes comes from the name of a representative sequence from the class. Two exceptions are Class “not G4” and Class “no clear pattern”, for which a representative sequence cannot be identified. A third exception is Class “17.4+17.4 s26” which contains sequences with characteristics of both Class 17.4 and Class 17.4 s26. Some classes are made up of smaller subclasses. The properties of each subclass are similar to those of the corresponding major class and subclasses are defined based on distinctive features of the NMR spectrum such as the presence of a specific signal or a lower signal to noise ratio. The name of each subclass is a name of its parental class plus a letter. For example, Class 17.4 can be further divided into Class 17.4a and Class 17.4b. The term “major class” refers to classes which are not subclasses.

Even a single mutation in a G‐quadruplex tetrad can induce formation of structures quite different from a monomeric G‐quadruplex (for example^[5]^). Therefore, when the phrase “a sequence with one mutation in the central tetrad” is used, it means that this sequence contains a point mutation at one of the four positions that form the central tetrad in the monomeric reference quadruplex without implying anything about the three‐dimensional structure of the mutant. The same holds for sequences that contain multiple mutations in the central tetrad and/or loops.

### Sorting of Sequences Into Classes

#### Manual Inspection

^1^H NMR spectra were visually inspected and sorted into classes based on the pattern of signals in the 10 to 12 ppm region. Parameters for sorting included the number of signals, the positions of signals, the signal to noise ratio, and the absence or presence of differences between ^1^H NMR spectra measured at two different timepoints.

### Computer Clustering

Class assignments were independently evaluated using computer clustering. Spectra were trimmed to only contain regions with signals corresponding to G‐quadruplex imino protons. They were then scaled on a 0–1 scale. Spectra with no signals were filtered out, and the remaining spectra were clustered. For more details, see the Supplementary Information Section SI_clustering.

### Final Sorting of Sequences into Classes

Manual inspection was better at identifying major classes with significant spectral differences while computer clustering was better at identifying subclasses with minor spectral differences. Therefore, we defined classes based on results from manual inspection and added some subclasses based on results from computer clustering (Table S13).

### Structural Determination

HPLC purified DNA was purchased from Sigma. DNA was resuspended in Milli‐Q water, heated at 65 °C for 5 min, cooled at room temperature for 5 minutes, and folded by adding buffer. Final concentrations were 10 μM DNA, 20 mM Tris, pH 7.5, 200 mM KCl and 1 mM MgCl_2_ in a volume of 70 ml. Samples were further purified using MonoQ ion‐exchange chromatography (1 ml volume, GE Healthcare) using a linear gradient from 0.2 to 1 M KCl. Eluted fractions were pooled, diluted to restore the KCl concentration to 200 mM, and concentrated using Amicon centrifugal filter units (cutoff 3 kDa). The buffer was also changed to d‐Tris during the concentration. The final DNA concentration was 1.7 mM in a volume of 350 μl.

NMR experiments were performed on a Bruker Avance III HD 850 MHz system equipped with an inverse triple resonance cryo‐probe. Sample contained 90 % H_2_O and 10 % D_2_O. A trace amount of DSS was added as a frequency standard. Spectral assignments were made using NOESY and TOCSY spectra at various temperatures and mixing times. Spectral analyses were performed using TOPSPIN (Bruker) and Sparky.[^34^, ^35^] All spectral assignments were made based on similarity with the previously solved structure of sequence 17.3,^[19]^ which differs from the analyzed sequence by a single point mutation in the loop.

NOE distance restraints were obtained from a NOESY spectrum acquired in H_2_O at 200 ms. For non‐exchangeable protons, the peaks were classified as strong, medium, or weak corresponding to distance restraints of 2.7±0.8, 3.8±0.9, or 5.5±1.7 Å, respectively. Distances from exchangeable protons were classified as strong, medium, or weak corresponding to distance restraints of 3.6±0.9, 4.8±1.2 or 5.5±1.7 Å, respectively.

Dihedral angle restraints were imposed to the dihedral angle formed by O4’–C1’–N9–C4 of guanosine residues, which was restricted to an angle of 240±70°. Hoogsteen hydrogen bonds between guanosines were restrained using H21–N7, N2–N7, H1–O6 and N1–O6 distances, which were set to 2.0±0.2, 2.9±0.3, 2.0±0.2 and 2.9±0.3 A°, respectively. Planarity restraints were used for the G1–G5–G10–G14, G2–G6–G11–G15 and G3–G7–G12–G16 tetrads.

## Conflict of Interests

The authors declare no conflict of interest.

1

## Supporting information

As a service to our authors and readers, this journal provides supporting information supplied by the authors. Such materials are peer reviewed and may be re‐organized for online delivery, but are not copy‐edited or typeset. Technical support issues arising from supporting information (other than missing files) should be addressed to the authors.

Supporting Information

## Data Availability

All data used in this study will be made available upon reasonable request.
