# Supplementary material for: NMR Screen Reveals the Diverse Structural Landscape of a G‐Quadruplex Library
Source: Chemistry. 2024 Nov 11;30(67):e202401437. doi: 10.1002/chem.202401437 (PMC11610706; doi:10.1002/chem.202401437)
Supplement: Supplementary file 1 — Supporting Information [file CHEM-30-e202401437-s001.pdf]

Chemistry–A European Journal

Supporting Information

NMR Screen Reveals the Diverse Structural Landscape of a G-Quadruplex Library

Ráchel Sgallová, Martin Volek, Jaroslav Kurfürst, Pavel Srb, Václav Veverka, and Edward A. Curtis*

NMR screen reveals the diverse structural landscape of a G-quadruplex library

Ráchel Sgallová[1,2], Martin Volek[1,3], Jaroslav Kurfürst[1,4], Pavel Srb[1], Václav Veverka[1,5], and Edward A. Curtis[1*]

[1]Institute of Organic Chemistry and Biochemistry of the Czech Academy of Sciences, Prague 166 10, Czech Republic

[2]Department of Low-Temperature Physics, Faculty of Mathematics and Physics, Charles University in Prague, Prague 180 00, Czech Republic

[3]Department of Genetics and Microbiology, Faculty of Science, Charles University in Prague, Prague 128 44, Czech Republic

[4]Department of Informatics and Chemistry, University of Chemistry and Technology, Prague 166 28, Czech Republic

[5]Department of Cell Biology, Faculty of Science, Charles University in Prague, Prague 128 44, Czech Republic

Phone: +420 733 169 654
E-mail: curtis@uochb.cas.cz

*Corresponding author

Table of Contents of Supplementary Information

Section SI_main: Figures S1 to S12, Tables S1 to S3
Section SI_classes: Figures S13 to S38
Section SI_table: Table S4
Section SI_clustering: Figures S39 to S41, Tables S5 to S14
Section SI_structure: Figures S42 to S43
Section SI_spectra: NMR spectra of all sequences, not numbered
Zip file spectra_of_all_sequences: NMR spectra of all sequences in TopSpin format, available at https://zenodo.org/records/13838249

Section SI_main ... 3

Figure S1: Figure of all classes including subclasses ... 4

Figure S2: Ion-exchange chromatography of model samples ... 5

Figure S3: Comparison of ^{1}H NMR spectra of sequences 17.3 and 17.50 ... 5

Figure S4: Time development of 17.28 and 17.154 ... 6

Figure S5: Time development of 17.29 ... 7

Figures S6 – S10: Native gels of all sequences chosen for the secondary screen ... 8

Figure S11: Standard deviations of biochemical activities for individual classes ... 12

Figure S12: Illustration of the A rule ... 13

Table S1: List of all classes ... 13

Table S2: List of all sequences characterized in the secondary screen and peaks in their ion-exchange chromatographs ... 14

Table S3: Results of native gels of all sequences analyzed in the secondary screen ... 16

Section SI_classes ... 18

Figures S13 – S38: ^{1}H NMR spectra and ion exchange chromatographs of representatives of each class ... 18

Section SI_table ... 28

Table S4: List of all sequences ... 28

Section SI_clustering ... 37

Methods ... 37

Computer clustering ... 37

Figures S39 – S41: comparison of ^{1}H NMR spectra of subclasses ... 38

Table S14: Detailed results of computer clustering ... 41

Section SI_structure ... 52

Structure of sequence 17.3 9C ... 52

Section SI_spectra ... 54

References ... 97

Section SI_main

Figure S1: Figure of all classes including subclasses

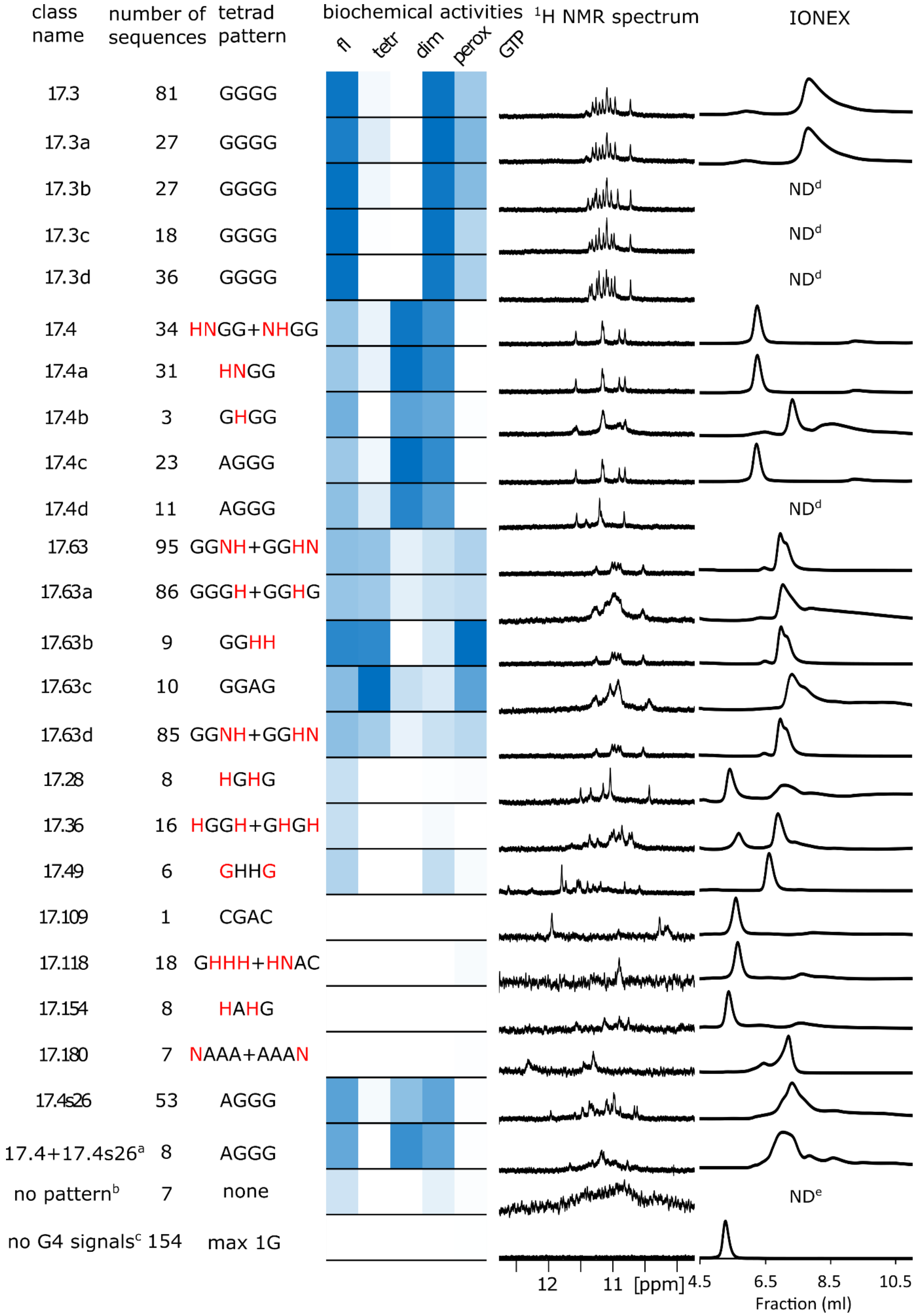

Figure S1: List of all spectral classes identified in this study (including subclasses). First column: class name; second column: number of sequences in the indicated class; third column: main sequence of the central tetrad of the indicated class

(corresponding to positions 2, 6, 11, and 15, which form the central tetrad of the reference G-quadruplex 17.3); fourth column: graphical representation of five previously measured biochemical activities ("fl" is the ability to generate fluorescence, "tetr" is the ability to form tetramers, "dim" is the ability to form dimers, "perox" is the ability to promote a model peroxidase reaction, and "GTP" is the ability to bind GTP) [15, 19, 20, 21, 22]; fifth column: ^{1}H NMR spectrum of a representative sequence in the class. Note that intensities in different spectra are not comparable, as not all spectra were measured with default parameters; sixth column: ion-exchange chromatogram of a representative sequence in the class. Note that intensities in different traces are not comparable. [a]: "17.4+17.4s26" is a class of sequences with ^{1}H NMR spectra with characteristics of both class 17.4 and 17.4s26, [b]: Class "no pattern" contains sequences with ^{1}H NMR spectra containing signal in G-quadruplex part of spectrum with no clear pattern, [c]: Class "no G4signals" contains all sequences with no signals in G-quadruplex part of ^{1}H NMR spectrum, [d]: Subclasses were not considered when representatives for secondary screen were chosen, so not all of subclasses have a representative ion-exchange chromatogram, [e]: class "no pattern" is not a proper class, so it is not possible to choose a representative sequence for the secondary screen.

Figure S2: Ion-exchange chromatography of model samples

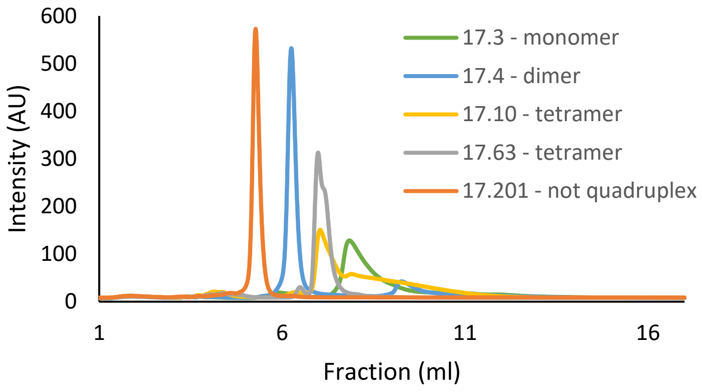

Figure S2: ion-exchange chromatography of model samples

Figure S3: Comparison of ^{1}H NMR spectra of sequences 17.3 and 17.50

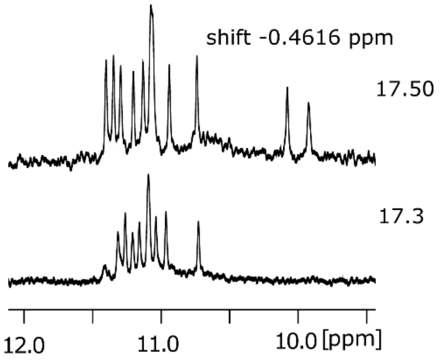

Figure S3: Comparison of the ^{1}H NMR spectra of sequences 17.3 and 17.50 as representatives of Class 17.49. The spectrum of sequence 17.50 was measured with 1024 scans and was shifted by -0.4616 ppm to better show the similarities between the peak patterns of Classes 17.3 and 17.49.

Figure S4: Time development of 17.28 and 17.154

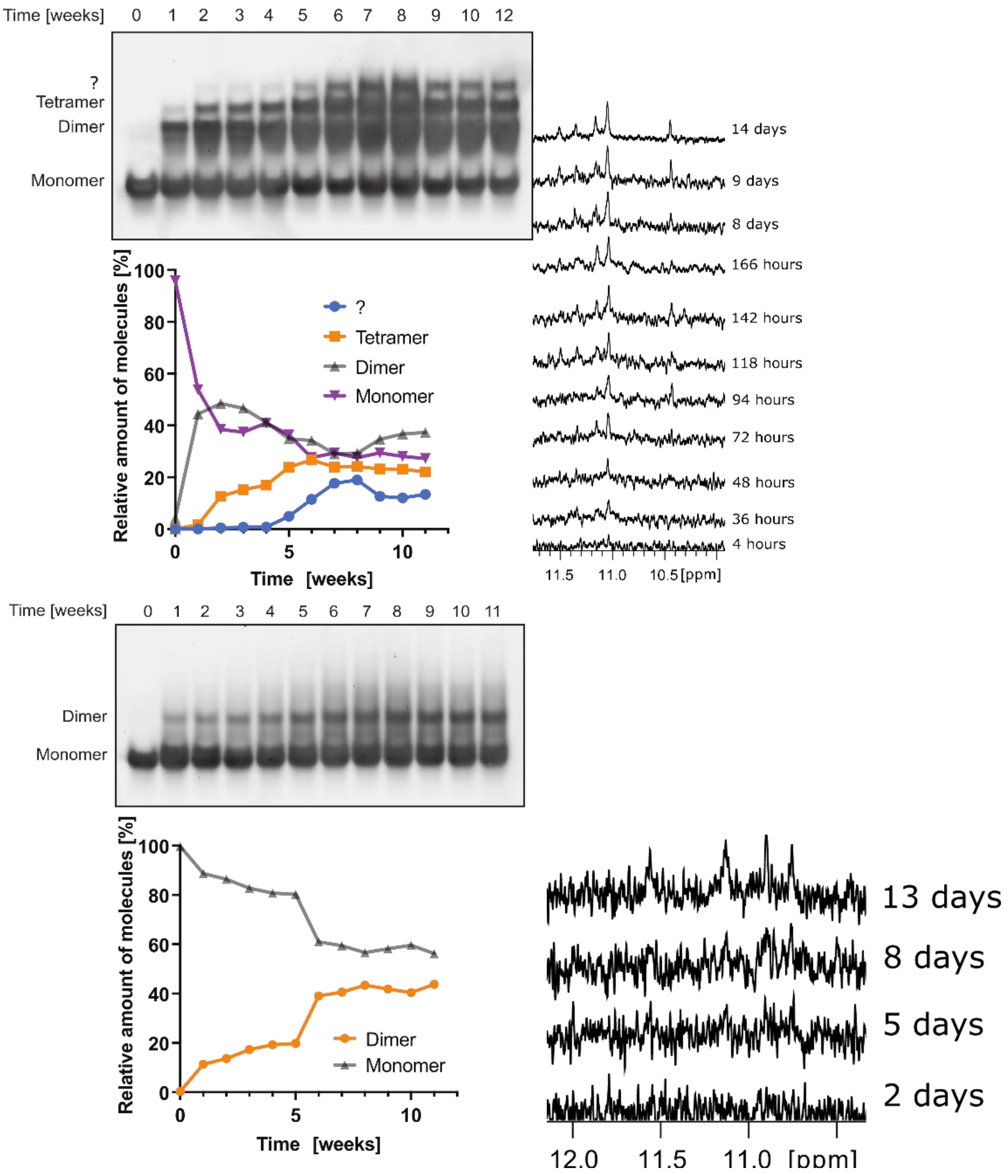

Figure S4: Slow folding sequences. Above: analysis of sequence 17.28. Top left: native gels immediately after preparation and after incubating for 1 to 11 weeks, bottom left: time development of relative amount of monomer (grey) and dimer (orange), right: ^{1}H NMR spectra measured 4 hours to 14 days after preparation. The spectrum measured after 14 days (top) was measured with 2048 scans and is displayed with scale 0.25, while all others were measured with 512 scans. Below: analysis of sequence 17.154. Top left: native gels immediately after preparation and after incubation for 1 to 11 weeks, bottom left: time development of relative amount of monomer (purple), dimer (grey), tetramer (orange), and a larger structure of unknown size (blue), right: ^{1}H NMR spectra measured 2 to 13 days after preparation. All spectra were measured with 1024 scans.

Figure S5: Time development of 17.29

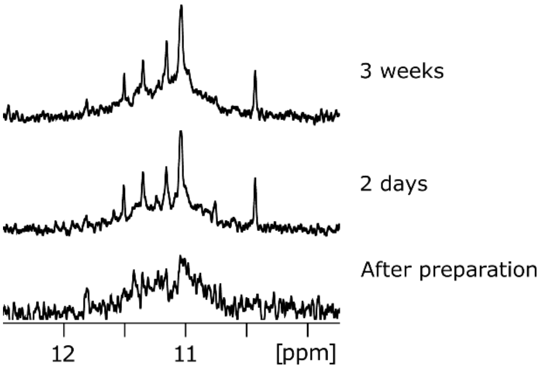

Figure S5: Time development of 17.29 showing delayed folding. Below: ^{1}H NMR spectrum after preparation, measured using 256 scans, scale 4, middle: after two days, measured using 1024 scans, above: after 3 weeks, measured using 1024 scans.

Figures S6 – S10: Native gels of all sequences chosen for the secondary screen

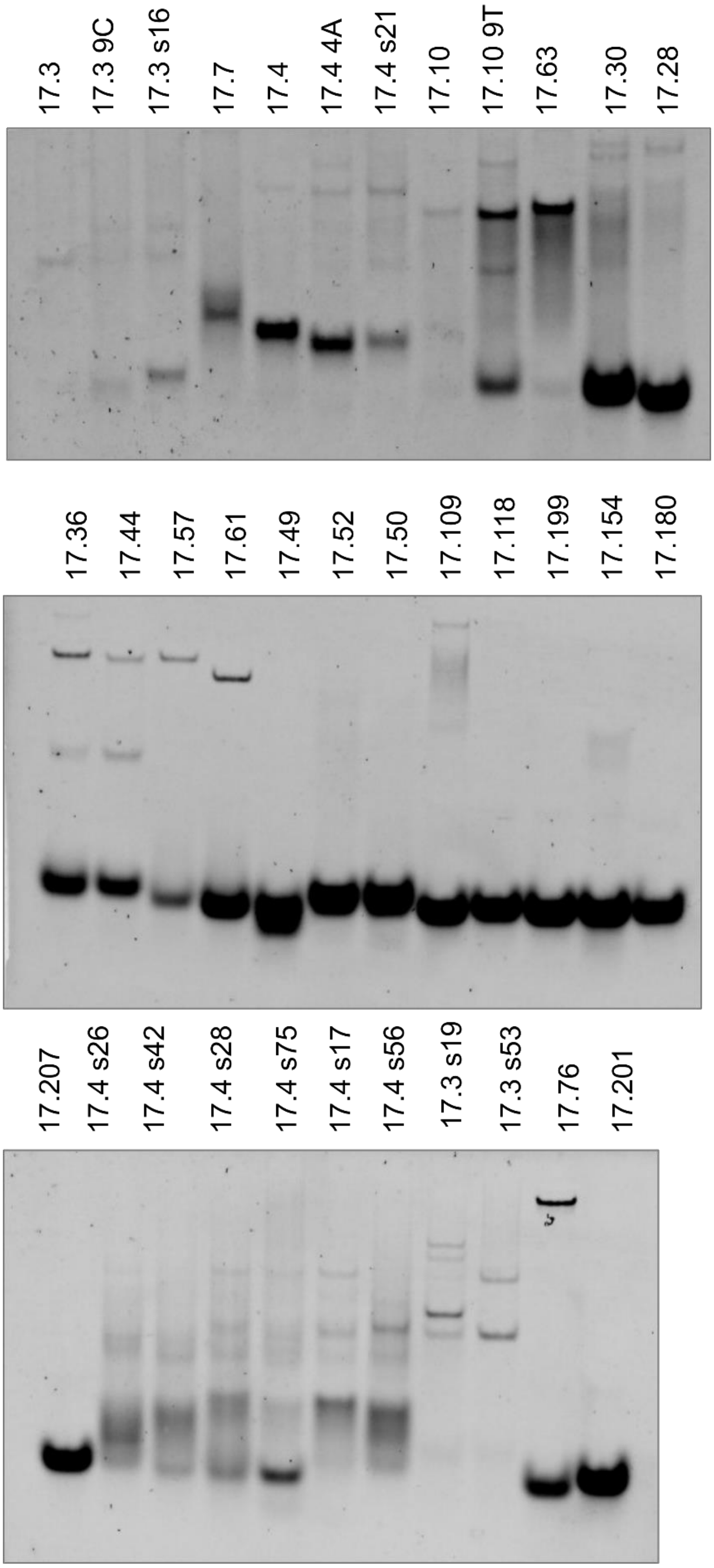

Figure S6: Native gels of all sequences (except 17.8) that were characterized in the secondary screen. Sequences were analyzed using "biochemical conditions" (described in the Methods).

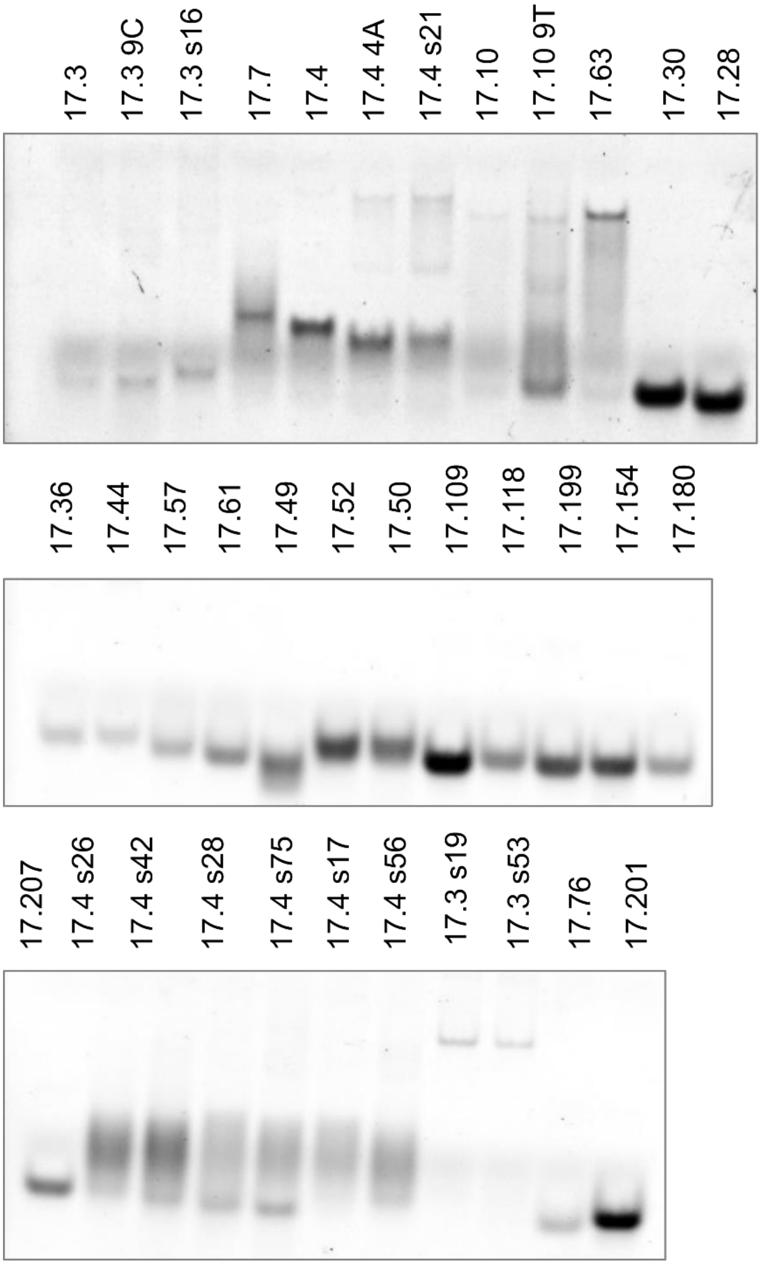

Figure S7: Native gels of all sequences (except 17.8) that were characterized in the secondary screen. Sequences were analyzed using "biochemical conditions with annealing" (described in the Methods).

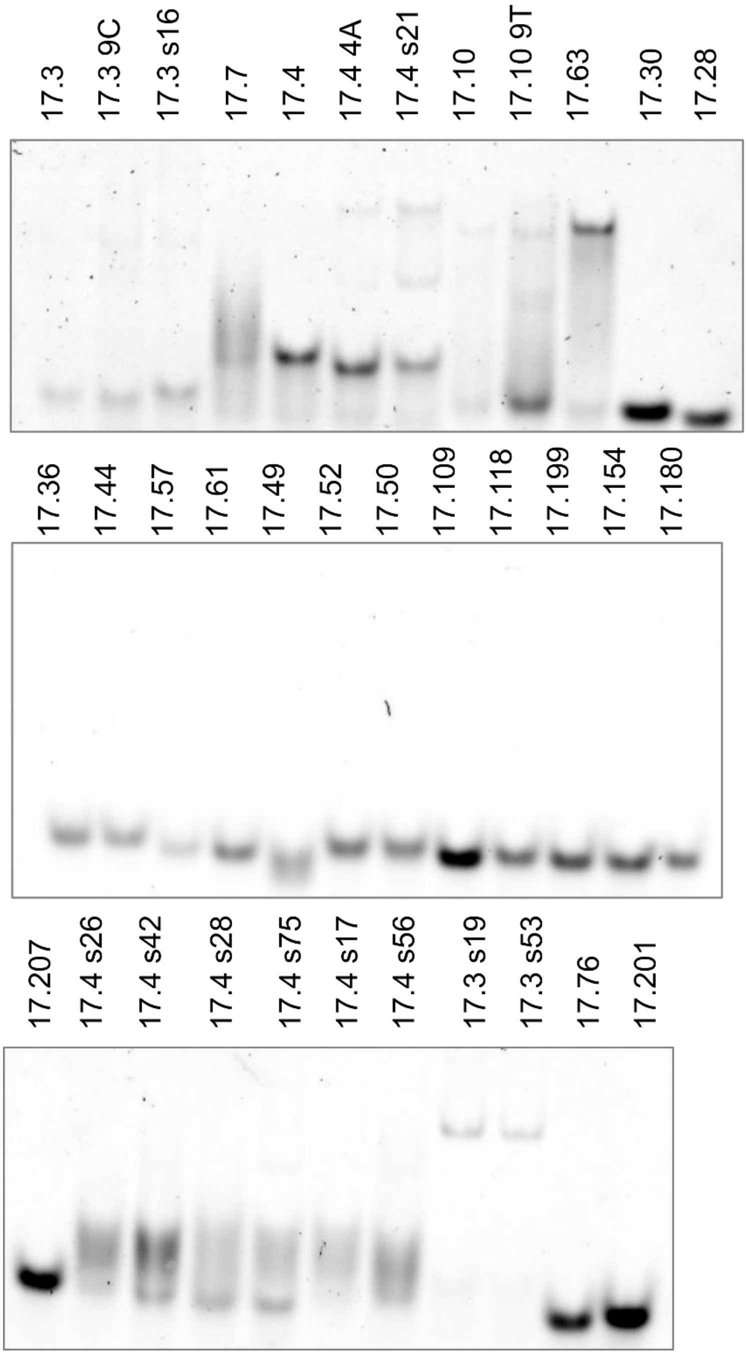

Figure S8: Native gels of all sequences (except 17.8) that were characterized in the secondary screen. Sequences were analyzed using " NMR conditions with low concentration" (described in the Methods).

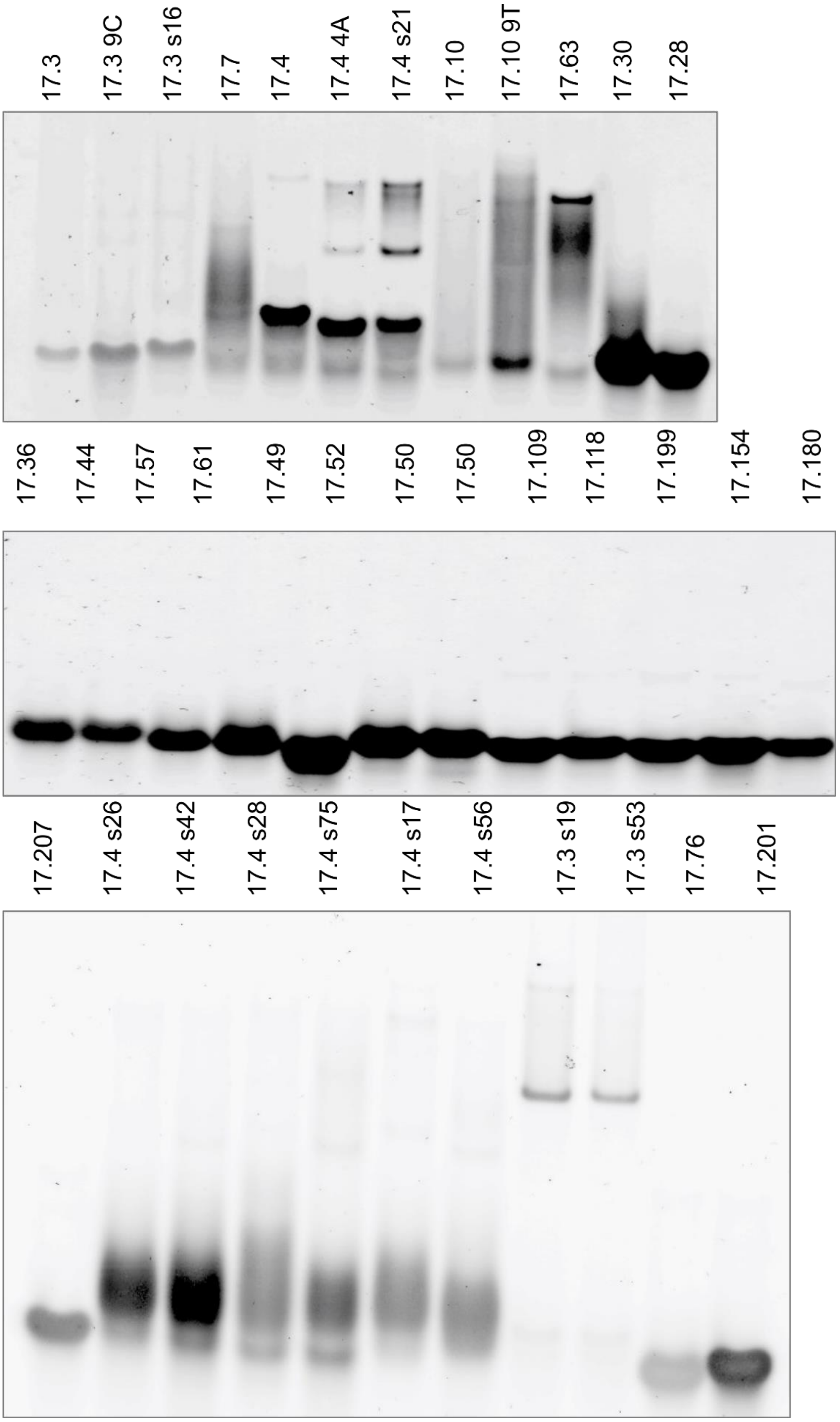

Figure S9: Native gels of all sequences (except 17.8) that were characterized in the secondary screen. Sequences were analyzed using " NMR conditions" (described in the Methods).

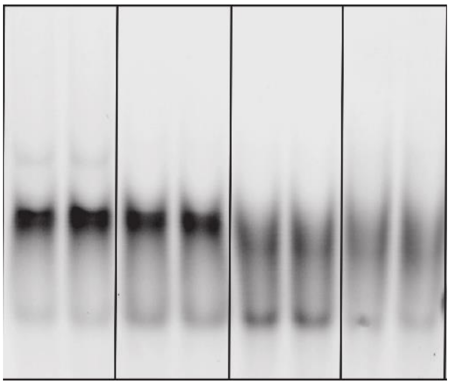

Figure S10: Native gels of sequence 17.8 analyzed using (from left to right) biochemical conditions (two replicates), biochemical conditions with annealing (two replicates), NMR conditions with low concentration (two replicates), and NMR conditions (two replicates). (all conditions are described in the Methods)

Figure S11: Standard deviations of biochemical activities for individual classes

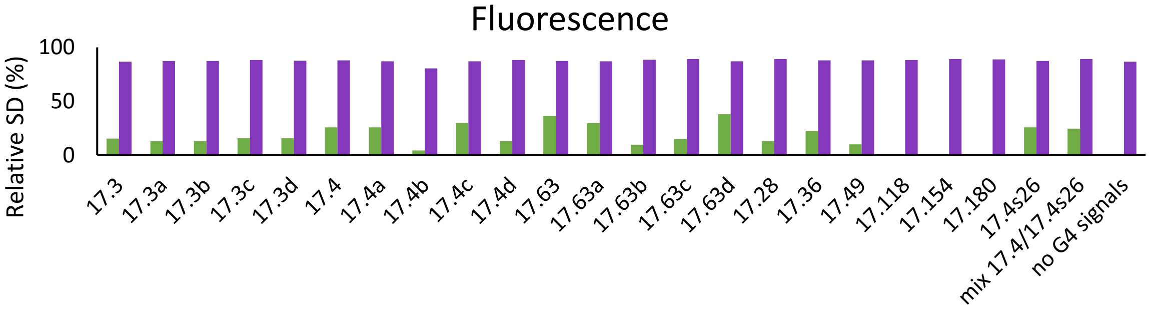

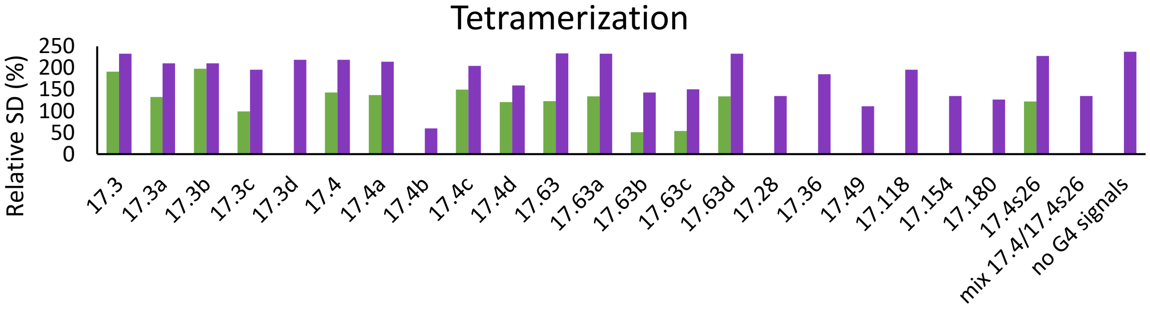

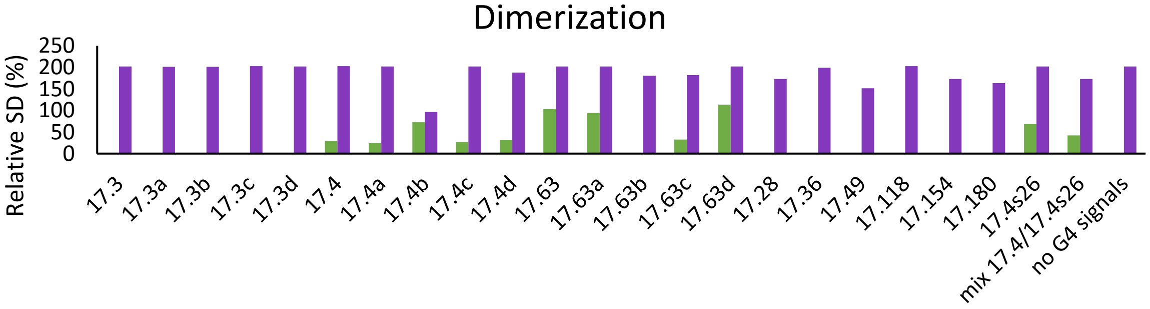

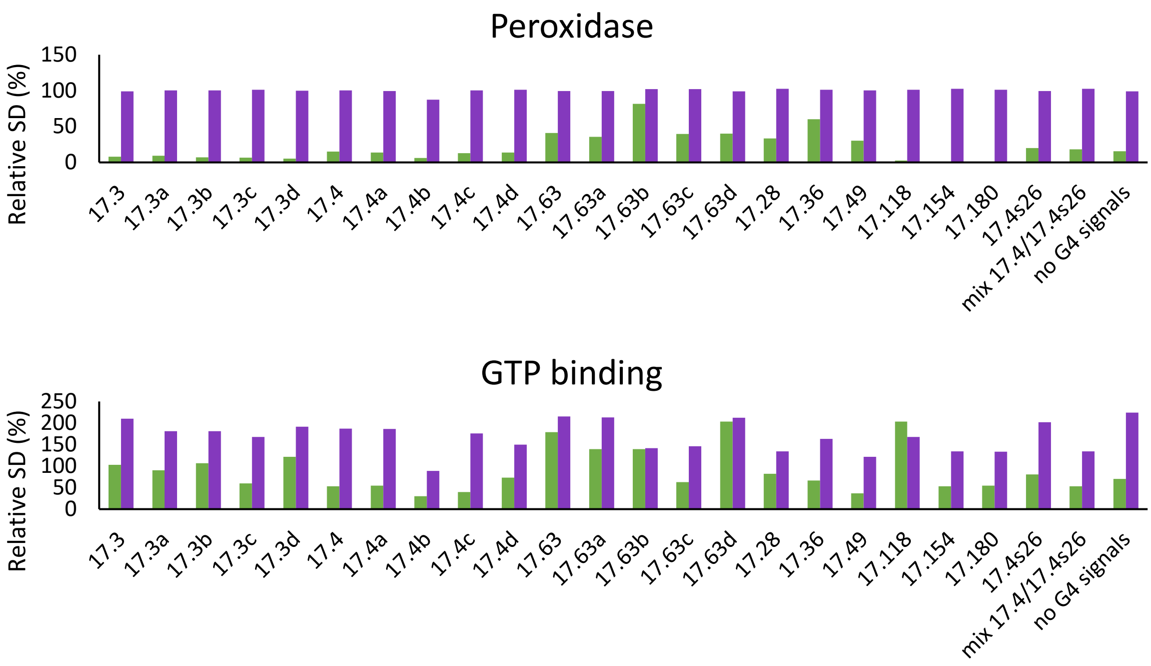

Figure S11: Relative standard deviation for all classes (green) and average relative standard deviations of a randomly chosen group with the same number of sequences (purple).

Figure S12: Illustration of the A rule

17.4

4 TACTTAAAAACCAAAAAAAA
8 AAAAACTAAAAACCTTAAAA
9 AAAAAAATAAAAAAAACCTT
2

17.4+17.4s26

4 TTACACCA
8 AAAACAAC
9 TTCTCTTC

17.4s26

4 TTTCCTTTCTTTTTTTTAAACCCCCCCCCCTTTTTTTTCCCCCCCCAAAAAAA
8 CTACTAAAACCCCTTTTCTTCCCCTTTTAACCCCTTTTTTTTCCCCCCCTTTT
9 AACAACCTCCTAACTAATCTCTAACTAACCCCTTCCTTCCTTCCTTCTTCCTT

Figure S12: Nucleotides at positions 4, 8, and 9 in sequences in different classes in the 17.4 loop library. Adenosines are highlighted to visualize the connection between the number of adenosines at positions 4, 8, and 9 and the spectral class of a sequence. All sequences in Class 17.4 contain 3 or 2 adenosines at those positions, all sequences in Class 17.4+17.4s26 contain 2 or 1 adenosines, and all sequences in Class 17.4s26 contain 1 or 0 adenosines.

Table S1: List of all classes

Table S1: List of all classes. "17.4+17.4s26" is a class of sequences with ^{1}H NMR spectra with characteristics of those in both Class 17.4 and Class 17.4s26, Class "no pattern" contains sequences with ^{1}H NMR spectra containing signals in the G-quadruplex part of spectrum with no clear pattern, and Class "no G4 signals" contains all sequences with no signals in the G-quadruplex part of ^{1}H NMR spectrum. N represents A, C, G, or T, H represents A, C, or T, Y represents C or T, M represents A or C, and W represents A or T.

Class name	Number of sequences	Tetrad sequence pattern	Loop sequence pattern	Number of sequences in secondary screen
17.3	81	GGGG	HHHH	5
17.3a	27	GGGG	HAHH	5
17.3b	27	GGGG	HHTH	0
17.3c	18	GGGG	HYAH	0

17.3d	36	GGGG	HYYH	0
17.4	34	NHGG + HNGG (not all loop mutations)	HHHH	4
17.4a	31	HNGG	HHHH	3
17.4b	3	GHGG	TAAT	2
17.4c	23	AGGG	HAAH	3
17.4d	11	AGGG	AAYH + AYAH	0
17.63	95	GGHN + GGNH	HHHH	3
17.63a	86	GGHG + GGGH	HHHH	2
17.63b	9	GGHH	TAAT	1
17.63c	10	GGAG	HAYW (10 out of 12)	1
17.63d	85	GGHN + GGNH	HHHH	2
17.28	8	HGHG (8 out of 9)		2
17.36	16	GHGH + HGGH (6 out of 9) + TGGT	TAAT	4
17.49	6	GHHG (6 out of 9)	TAAT	3
17.109	1	CGAC	TAAT	1
17.118	18	GHHH + HNAC	TAAT	2
17.154	8	HAHG (6 out of 9)	TAAT	1
17.180	7	NAAA + AAAN	TAAT	2
17.4s26	53	AGGG	HHHH	3
17.4 + 17.4s26	8	AGGG	HMHH	3
No clear pattern	7	No consensus	TAAT	0
No G4 signals	154	At least three mutations in tetrad (154 out of 189)	TAAT	2

Table S2: List of all sequences characterized in the secondary screen and peaks in their ion-exchange chromatographs

Table S2: List of all samples chosen for the secondary screen and information about the properties of their peaks in their ion-exchange chromatographs. If a sequence belongs to a Subclass, it also belongs to the relevant major class. Peak types were assigned according to model sequences (Table 1 *and Figure S2). If peak broadness is described as "??," it means that the peak overlaps with other peaks to such an extent that the broadness cannot be determined. "17.4+17.4s26" is a class of sequences with* ^{1}*H NMR spectra with characteristics of those in both Class 17.4 and 17.4s26, Class "no pattern" contains sequences with* ^{1}*H NMR spectra containing signals in the G-quadruplex part of spectrum with no clear pattern, and Class "no G4 signals" contains all sequences with no signals in the G-quadruplex part of* ^{1}*H NMR spectrum.*

Sequence name	Class name	Peaks				
		Peak type	Position (ml)	Intensity (AU)	Broadness (ml)	note
17.3	17.3a	Monomeric	7.8	130	0.8	
			5.9	19	0.7	
17.3 9C	17.3a	Monomeric	8.0	100	0.9	
		Tetrameric	7.2	20	0.1	
17.3s16	17.3a, 17.3b	Monomeric	9.0	80	1	
		Monomeric	8.4	43	??	
		Monomeric	7.9	30	??	
			12.2	18	??	
17.7	17.4b, 17.4c	Tetrameric	7.3	150	0.25	
		Monomeric	8.5	55	1	
		Dimeric	6.5	22	0.5	
			10.0	22	??	
17.8	17.4b, 17.4c	Monomeric	8.2	136	0.35	
		Monomeric	8.5	85	??	
		Monomeric	9.0	80	??	
		Tetrameric	6.9	25	??	

17.4	17.4a, 17.4c	Dimeric	6.3	520	0.3	
			9.3	42	0.5	
17.4 4A	17.4a, 17.4c	Dimeric	6.4	460	0.2	
			9.7	25	??	
			10.2	22	??	
17.4s21	17.4a, 17.4c	Dimeric	6.6	380	0.2	
			9.6	40	0.6	
			10.9	17	??	
17.10	17.63a, 17.63d	Tetrameric	7.0	150	0.5	
		Monomeric	7.9	57	??	
			4.2	20	0.4	
		Dimeric	6.3	20	??	long tail up to 11.5 ml
17.10 9T	17.63a, 17.63c	Tetrameric	7.3	115	0.7	long tail up to 12 ml
17.63	17.63b, 17.63d	Tetrameric	7.0	310	0.4	
		Dimeric	6.5	28	0.2	
			4.4	18	??	
17.30	17.28	Tetrameric	7.2	130	1	
		Monomeric	7.7	65	??	
			5.5	25	0.4	
			4.3	17	??	long tail up to 13 ml
17.28	17.28	NotG4	5.4	70	0.3	
		Tetrameric	7.1	40	0.8	
		Monomeric	8.0	25	??	long tail up to 13 ml
17.36	17.36	Tetrameric	6.9	165	0.2	
		Many mutations	5.7	80	0.3	
			7.5	40	??	
			11.5	30	0.7	
17.44	17.36	Tetrameric	7.0	100	0.8	
		NotG4	5.4	34	0.4	
			10.2	18	??	
			11.6	25	0.7	
17.57	17.36	Tetrameric	7.0	260	0.4	
		Monomeric	7.5	70	??	
			5.0	17	??	
17.61	17.36	Tetrameric	7.1	122	0.4	
			5.5	50	0.4	
			4.6	20	??	long tail up to 11 ml
17.49	17.49	Dimeric	6.6	400	0.25	
			5	18	??	
			7.6	18	??	
17.52	17.49	Dimeric	6.5	370	0.25	
		Tetrameric	7.2	33	??	
			4.6	17	??	
17.50	17.49	Dimeric	6.5	380	0.2	
			7.5	23	??	
17.109	17.109	Many mutations	5.6	432	0.2	
		Monomeric	8.0	40	??	
			10.3	23	??	
17.118	17.118	Many mutations	5.6	380	0.3	
		Monomeric	7.7	63	0.5	
		Dimeric	6.7	30	??	
			11.0	20	??	
17.199	17.118	Many mutations	5.6	490	0.2	

17.154	17.154	NotG4	5.4	270	0.2	
			7.6	46	0.7	
		Dimeric	6.4	30	??	
			4.6	20	??	
17.180	17.180	Tetrameric	7.2	270	0.3	
		Dimeric	6.5	75	??	
17.207	17.180	Dimeric	6.2	470	0.25	
		NotG4	5.4	17	??	
17.4s26	17.4s26	Tetrameric	7.3	160	0.7	
		Monomeric	8.7	43	??	long tail up to 12.5 ml
17.4s42	17.4s26	Tetrameric	7.7	115	0.7	long tail up to 12.5 ml
17.4s28	17.4s26	Tetrameric	6.9	100	0.5	
		Monomeric	7.5	105	??	
		Monomeric	7.6	110	??	
			9.2	8.5	0.4	long tail up to 14 ml
17.4s75	17.4 + 17.4s26	Dimeric	6.4	250	0.3	
		Dimeric	5.9	50	0.3	
		Monomeric	8.2	57	??	
			8.9	75	??	long tail up to 12.5 ml
17.4s17	17.4 + 17.4s26	Dimeric	6.5	220	0.4	
		Monomeric	7.5	125	0.5	long tail up to 12.5 ml
17.4s56	17.4 + 17.4s26	Tetrameric	7.0	155	0.8	
		Monomeric	7.9	60	??	
		Monomeric	8.6	50	0.6	long tail up to 12.5 ml
17.3s19	17.3a, 17.3b	Monomeric	8.5	95	0.6	
		Tetrameric	7.3	50	0.3	
		Dimeric	6.3	21	0.3	long tail up to 11.5 ml
17.3s53	17.3b, 17.3d	Monomeric	8.7	80	0.7	
		Tetrameric	7.4	40	0.3	
		Dimeric	6.3	20	0.3	
17.76	No G4 signals	NotG4	5.2	125	0.3	
		Tetrameric	7.0	120	0.5	
17.201	No G4 signals	NotG4	5.3	560	0.2	

Table S3: Results of native gels of all sequences analyzed in the secondary screen

Table S3: Results of native gels for all sequences analyzed in the secondary screen in all four sets of conditions. "17.4+17.4s26" is a class of sequences with ^{1}H NMR spectra with characteristics of those in both Class 17.4 and 17.4s26, Class "no pattern" contains sequences with ^{1}H NMR spectra containing signals in the G-quadruplex part of the spectrum with no clear pattern, and Class "no G4 signals" contains all sequences with no signals in the G-quadruplex part of the ^{1}H NMR spectrum. Results are in percentages. If multimeric state is not specified elsewhere, it is indicated in the "Result" column. Conditions are in detail described in the Methods.

Sequence name	Class name	Biochemical conditions	Bio. con. + annealing	NMR conditions + low conc.	NMR conditions	Result
17.3	17.3	100±0	100±0	100±0	100±0	Monomer
17.3 9C	17.3	100±0	100±0	100±0	100±0	Monomer
17.3s16	17.3	100±0	100±0	100±0	100±0	Monomer
17.7	17.4b	98,0±0,6	96±2	80±20	92±9	Dimer
17.8	17.4b	82±12	79±11	76±10	74±9	Dimer
17.4	17.4a	99±1	99,1±0,3	98±1	99,6±0,2	Dimer
17.4 4A	17.4a	Dimer 95,9±0,4 and tetramer 3,58±0,07	Dimer 94±3, trimer 4±1, and tetramer 7±5	Dimer 90±4, trimer 2±2, and tetramer 4±2	Dimer 93±2, trimer 2±2, and tetramer 5±2	Dimer, tetramer, and something between

17.4s21	17.4a	Dimer 88±5 and tetramer 12±4	Dimer 90±4, trimer 4±1, and tetramer 7±5	Dimer 71±6, trimer 13±4, and tetramer 12±1	Dimer 89±4, trimer 5±2, and tetramer 13±4	Dimer, tetramer, and something between
17.10	17.63a	40±20	31±2	28±2	11±2	Tetramer
17.10 9T	17.63a	Dimer 4±1 and tetramer 39±9	7±1	6±1	8±03	Tetramer
17.63	17.63b	96±3	84±2	82±2	70±20	Tetramer
17.30	17.28	100±0	100±0	100±0	100±0	Monomer
17.28	17.28	100±0	100±0	100±0	100±0	Monomer
17.36	17.36	Dimer 2.8±0.4 and tetramer 8±2	100±0	100±0	100±0	Monomer
17.44	17.36	Dimer 4.4±0.4 and tetramer 5±2	100±0	100±0	100±0	Monomer
17.57	17.36	Tetramer 11±3	100±0	100±0	100±0	Monomer
17.61	17.36	Tetramer 6.5±0.3	100±0	100±0	100±0	Monomer
17.49	17.49	100±0	100±0	100±0	100±0	Monomer
17.52	17.49	100±0	100±0	100±0	100±0	Monomer
17.50	17.49	100±0	100±0	100±0	100±0	Monomer
17.109	17.109	Tetramer 0.4±0.2	100±0	100±0	100±0	Monomer
17.118	17.118	100±0	100±0	100±0	100±0	Monomer
17.199	17.118	100±0	100±0	100±0	100±0	Monomer
17.154	17.154	100±0	100±0	100±0	100±0	Monomer
17.180	17.180	100±0	100±0	100±0	100±0	Monomer
17.207	17.180	100±0	100±0	100±0	100±0	Monomer
17.4s26	17.4s26	Dimer 72.8±0.9 and tetramer 8±3	92±6	90±8	80±5	Dimer
17.4s42	17.4s26	Dimer 80±10 and tetramer 7±4	90,3±0,4	82±1	80±20	Dimer
17.4s28	17.4s26	Dimer 50±10 and tetramer 7±3	72±3	50±20	80±20	Dimer
17.4s75	17.4 + 17.4s26	Dimer 30±10 and tetramer 13±2	75±1	67±2	80±7	Dimer
17.4s17	17.4 + 17.4s26	Dimer 80±20 and tetramer 11±8	98±2	90±10	89±7	Dimer
17.4s56	17.4 + 17.4s26	Dimer 70±20 and tetramer 11±8	86±7	70±10	78±2	Dimer
17.3s19	17.3	72±4	97±2	85±6	80±10	Tetramer
17.3s53	17.3	66±6	95±1	80±20	80±20	Tetramer
17.76	No G4 signals	16±8 really high band	100±0	100±0	100±0	Monomer
17.201	No G4 signals	100±0	100±0	100±0	100±0	Monomer

Section SI_classes

Figures S13 – S38: ^{1}H NMR spectra and ion exchange chromatographs of representatives of each class

NOTE: AU in ion exchange chromatographs is the same for all samples, so intensities are comparable in different graphs.

Default conditions for ^{1}H NMR spectra are first preparation, DNA concentration 100 µM, spectrum measured within three days of preparation, and 256 scans. If other parameters were used, this is specifically mentioned in the corresponding legend.

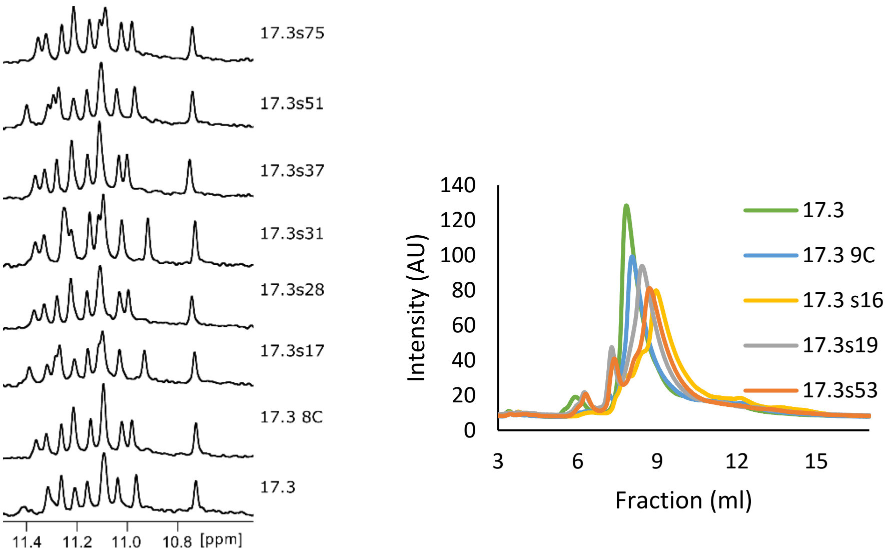

Figure S13: left: ^{1}H NMR spectra of randomly chosen representatives of Class 17.3. Right: ion exchange chromatographs of five representative sequences chosen for characterization in the secondary screen.

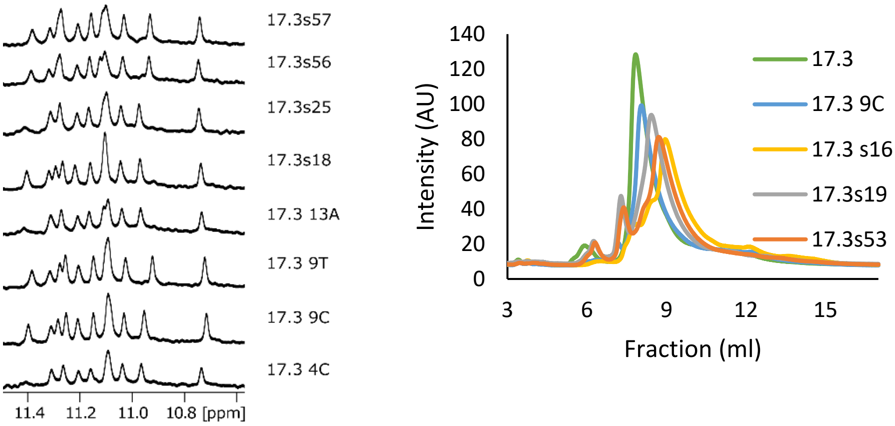

Figure S14: left: ^{1}H NMR spectra of randomly chosen representatives of Class 17.3a, right: ion exchange chromatographs of five representative sequences chosen for characterization in the secondary screen.

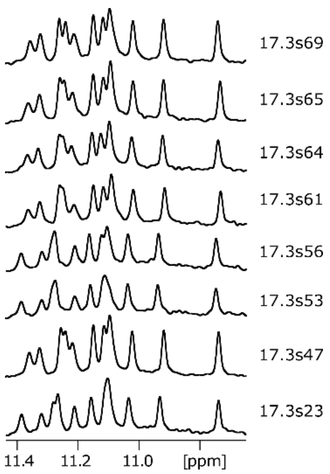

Figure S15: ^{1}H NMR spectra of randomly chosen representatives of Class 17.3b.

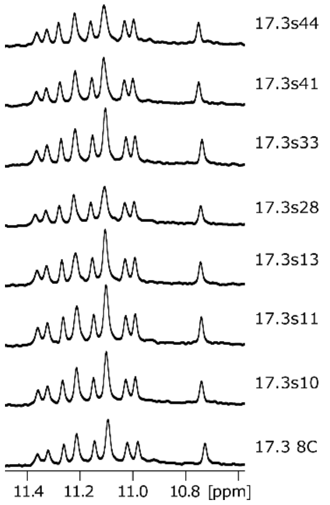

Figure S16: ^{1}H NMR spectra of randomly chosen representatives of Class 17.3c.

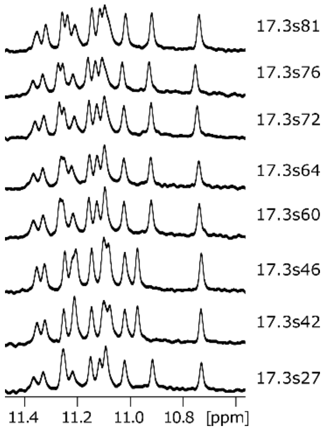

Figure S17: ^{1}H NMR spectra of randomly chosen representatives of Class 17.3d.

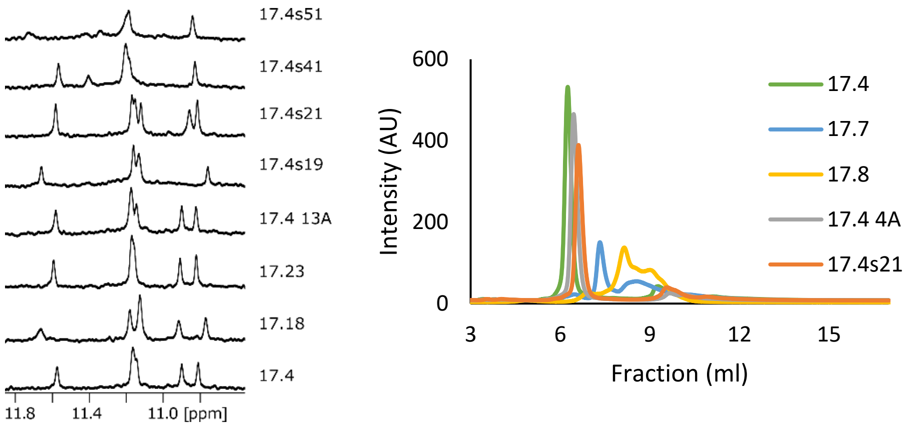

Figure S18: left: ^{1}H NMR spectra of randomly chosen representatives of Class 17.4. Right: ion exchange chromatographs of five representative sequences chosen for characterization in the secondary screen.

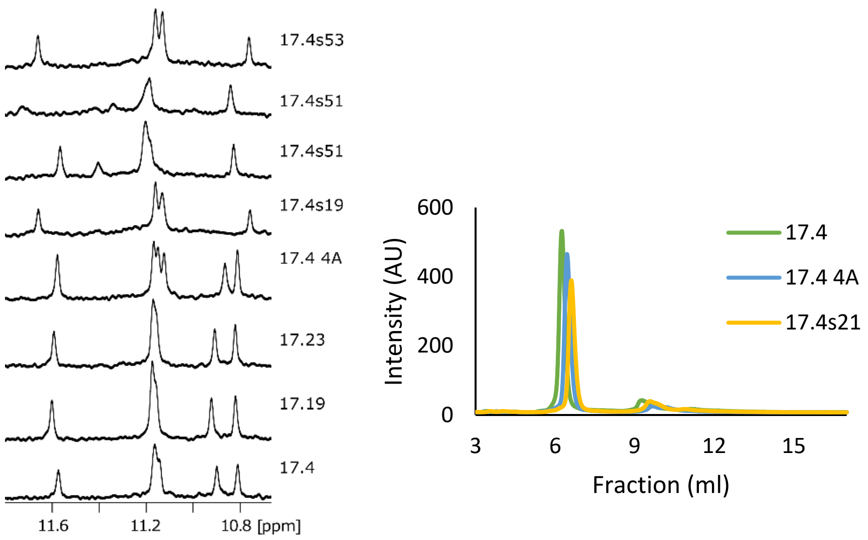

Figure S19: left: ^{1}H NMR spectra of randomly chosen representatives of Class 17.4a, right: ion exchange chromatographs of three representative sequences chosen for characterization in the secondary screen.

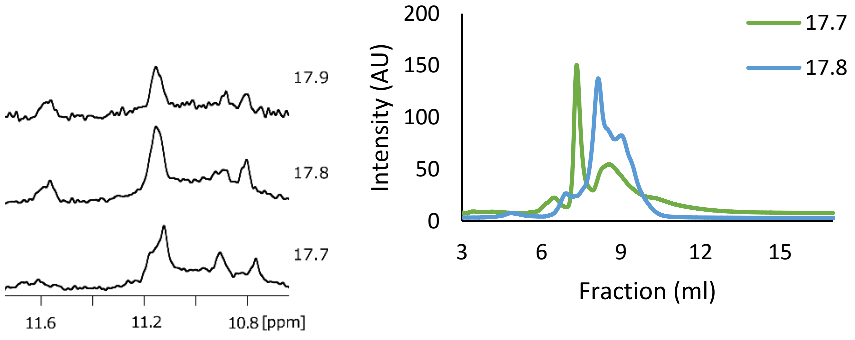

Figure S20: left: ^{1}H NMR spectra of all sequences in Class 17.4b. All spectra were measured with 1024 scans, and the spectrum of sequence 17.9 is displayed with scale 2. Right: ion exchange chromatographs of two representative sequences chosen for characterization in the secondary screen.

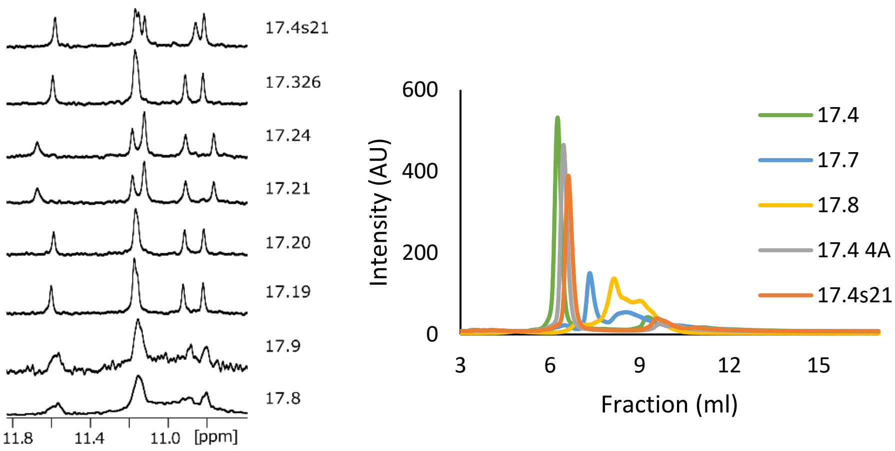

Figure S21: left: ^{1}H NMR spectra of randomly chosen representatives of Class 17.4c. The spectrum of sequence 17.8 was measured with 1024 scans and is displayed with scale 0.5, while the spectrum of sequence 17.9 was measured with 1024 scans and is displayed with scale 2. Right: ion exchange chromatographs of five representative sequences chosen for characterization in the secondary screen.

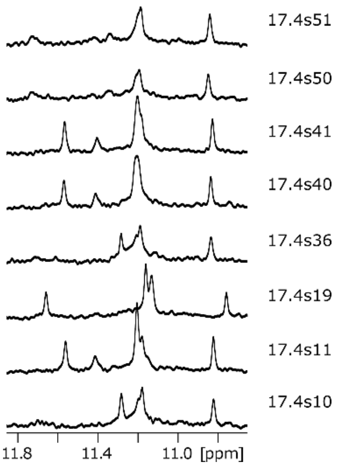

Figure S22: ^{1}H NMR spectra of randomly chosen representatives of Class 17.4d.

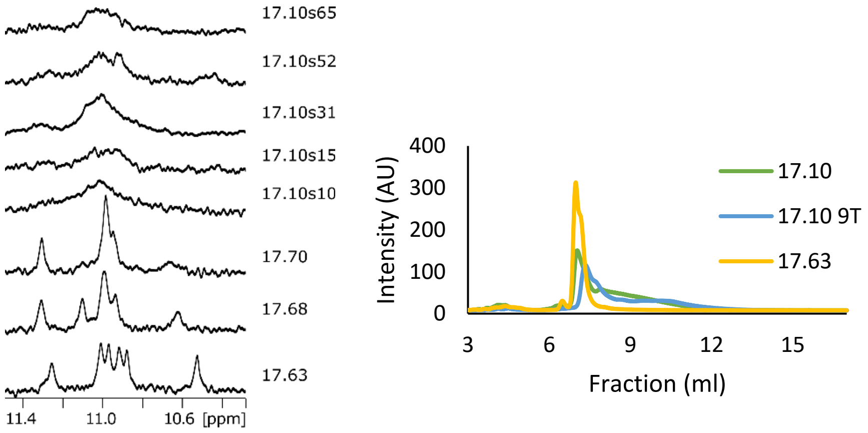

Figure S23: left: ^{1}H NMR spectra of randomly chosen representatives of Class 17.63. The spectrum of sequence 17.10s31 was measured with 1024 scans and is displayed with scale 0.25. Right: ion exchange chromatographs of three representative sequences chosen for characterization in the secondary screen.

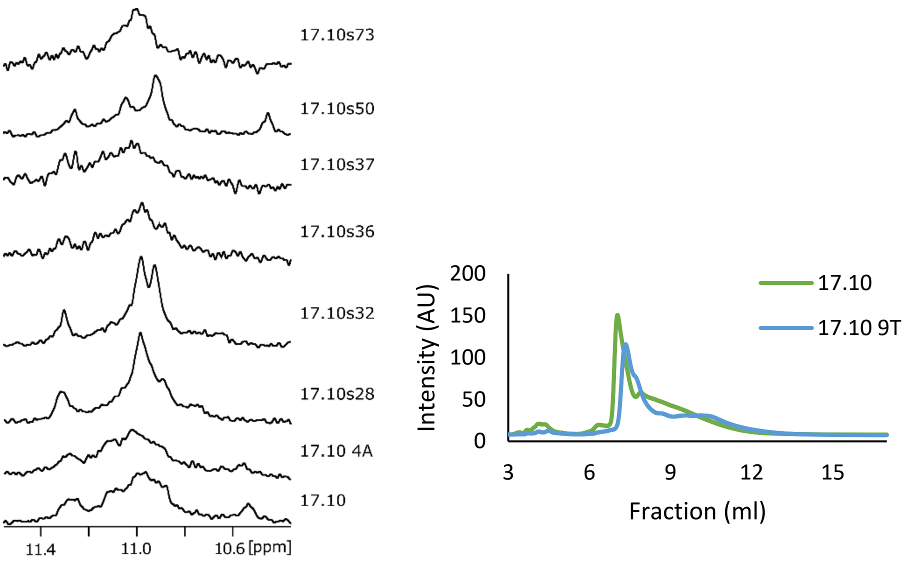

Figure S24: left: ^{1}H NMR spectra of randomly chosen representatives of Class 17.63a. The spectra of sequences 17.10s36, 17.10s37, and 17.10s73 are displayed with scale 4, while other spectra were measured with 1024 scans. Right: ion exchange chromatographs of two representative sequences chosen for characterization in the secondary screen.

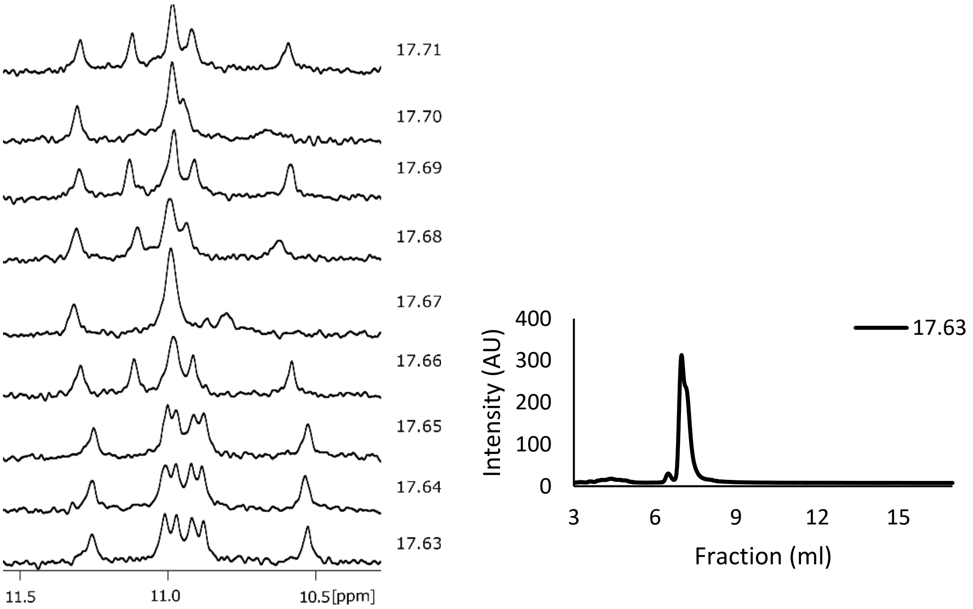

Figure S25: left: ^{1}H NMR spectra of all sequences in Class 17.63b. Right: Ion exchange chromatograph of a representative sequence chosen for characterization in the secondary screen.

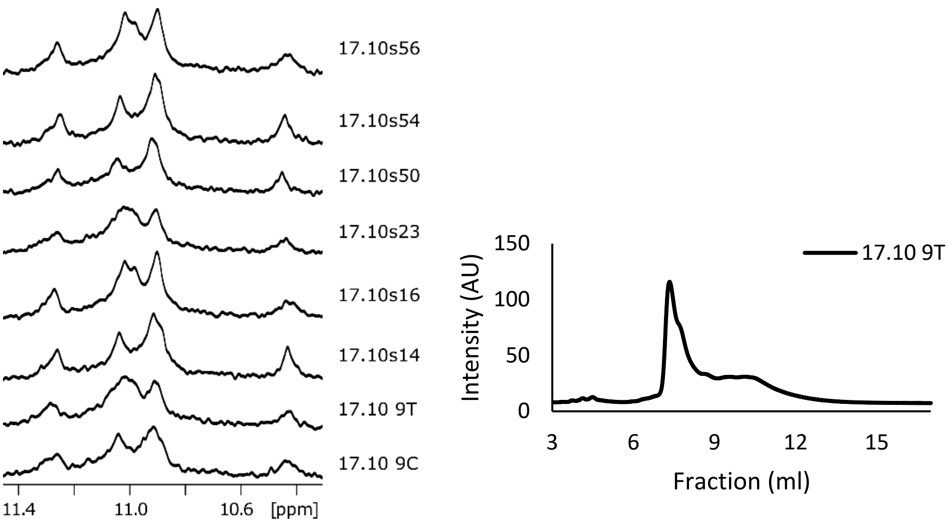

Figure S26: left: ^{1}H NMR spectra of randomly chosen representatives of Class 17.63c. All spectra were measured with 1024 scans. Right: ion exchange chromatograph of a representative sequence chosen for characterization in the secondary screen.

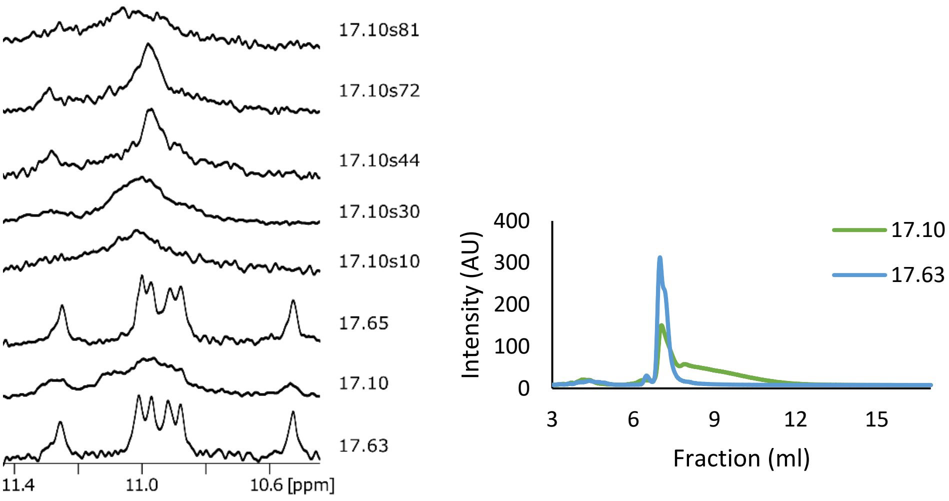

Figure S27: left: ^{1}H NMR spectra of randomly chosen representatives of Class 17.63d. The spectra of sequences 17.10 and 17.10s30 were measured with 1024 scans and are displayed with scale 0.25. Right: ion exchange chromatographs of two representative sequences chosen for characterization in the secondary screen.

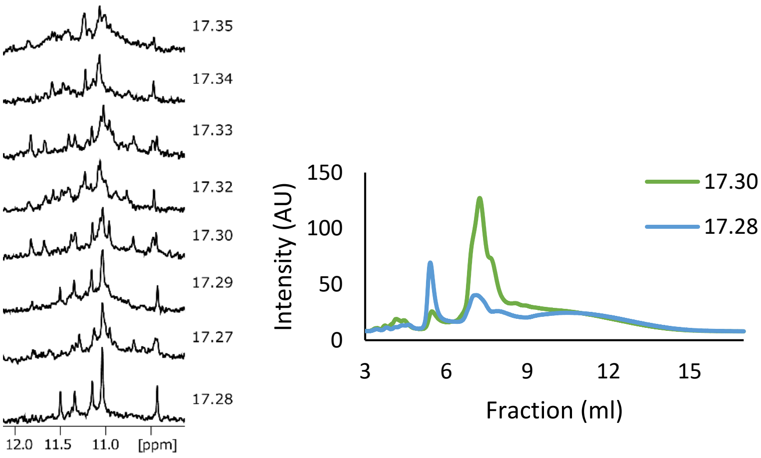

Figure S28: left: ^{1}H NMR spectra of all sequences in Class 17.28. All spectra were measured after weeks on the bench with 1024 scans. Right: ion exchange chromatographs of two representative sequences chosen for characterization in the secondary screen.

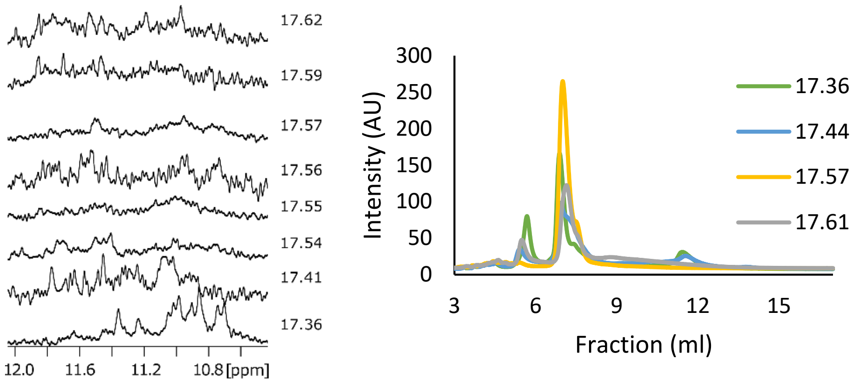

Figure S29: left: ^{1}H NMR spectra of randomly chosen representatives of Class 17.36. All spectra except for that of sequence 17.59 were measured after weeks on the bench. The spectra of sequences 17.36, 17.54, 17.55, and 17.57 were measured with 1024 scans, the spectrum of sequence 17.41 was measured with 512 scans with a sample from the second preparation with a DNA concentration of 50 µM and is displayed with scale 2, the spectrum of sequence 17.56 was measured with 512 scans with a sample from the second preparation with a DNA concentration of 50 µM and is displayed with scale 4, the spectrum of sequence 17.59 is displayed with scale 4, and the spectrum of sequence 17.62 was measured with 512 scans with a sample from the second preparation with a DNA concentration of 40 µM and is displayed with scale 2. Right: ion exchange chromatographs of four representative sequences chosen for characterization in the secondary screen.

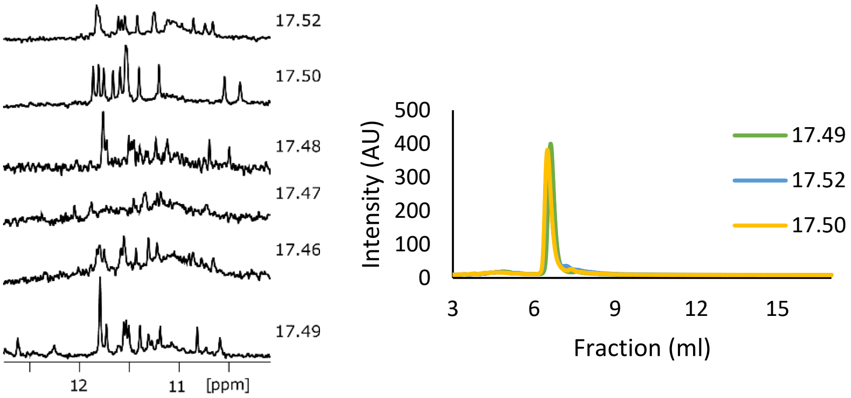

Figure S30: left: ^{1}H NMR spectra of all sequences in Class 17.49. The spectra of sequences 17.46, 17.47, and 17.52 were measured with 1024 scans after weeks on the bench, the spectrum of sequence 17.48 was measured using a sample from the second preparation with a DNA concentration of 50 µM after weeks on the bench, and the spectra of sequences 17.49 and 17.50 were measured with 1024 scans. Right: ion exchange chromatographs of three representative sequences chosen for characterization in the secondary screen.

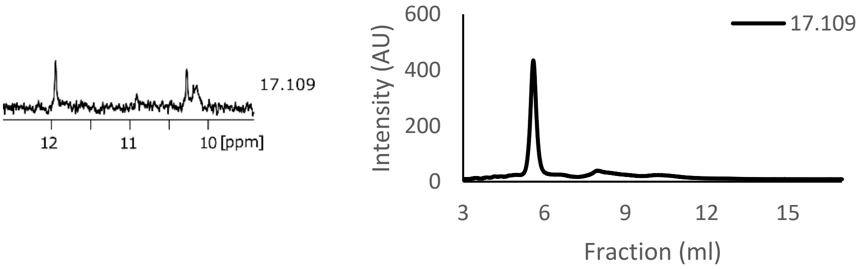

Figure S31: left: ^{1}H NMR spectrum of sequence 17.109 measured with 1024 scans. This is the only member of Class 17.109. Right: ion exchange chromatograph of sequence 17.109.

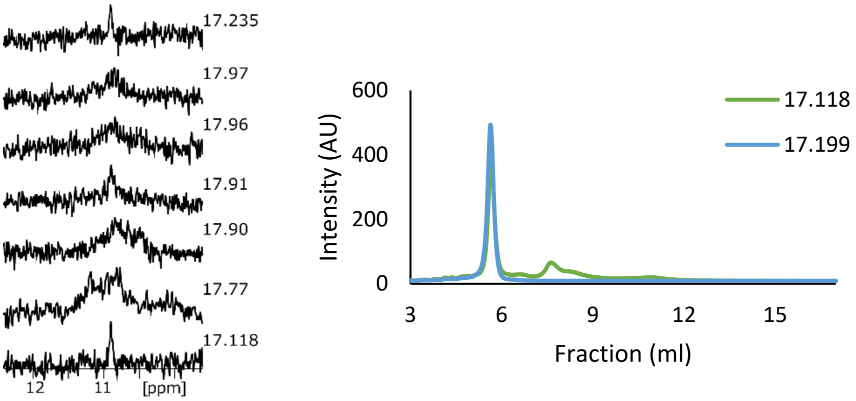

Figure S32: left: ^{1}H NMR spectra of randomly chosen representatives of Class 17.118. All spectra except that of sequence 17.118 were measured after weeks on the bench. All spectra except the spectrum of sequence 17.77 were measured with 1024 scans, while the spectrum of sequence 17.77 was measured with 2056 scans. Right: ion exchange chromatographs of two representative sequences chosen for characterization in the secondary screen.

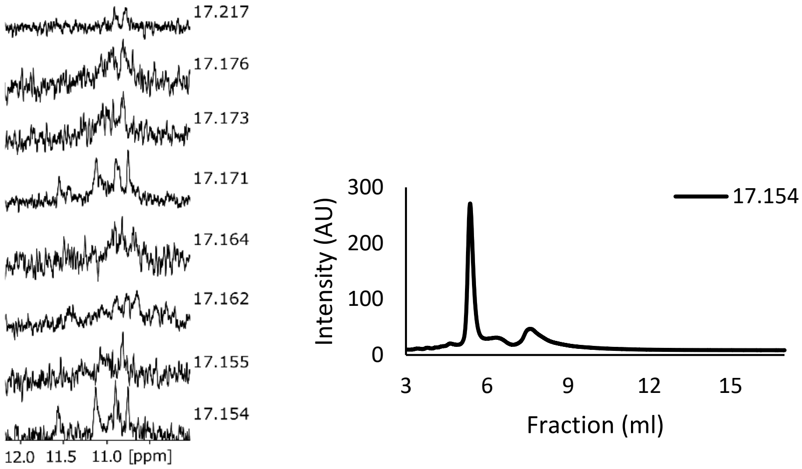

Figure S33: left: ^{1}H NMR spectra of all sequences in Class 17.154. All spectra were measured after weeks on the bench. Spectra of sequences 17.217, 17.171, and 17.162 were measured with 1024 scans and are displayed with scale 0.25, while the spectrum of sequence 17.176 was measured using a sample from the second preparation with a DNA concentration of 60 µM with 1024 scans and is displayed with scale 0.5. Right: ion exchange chromatographs of a representative sequence chosen for characterization in the secondary screen.

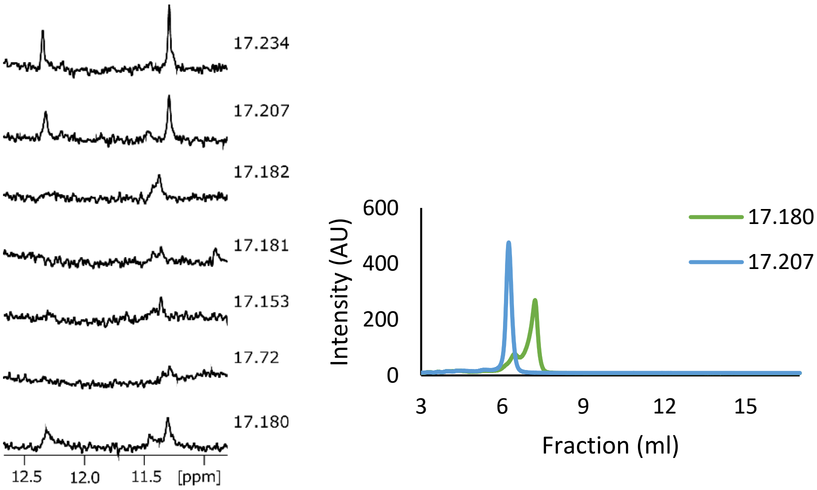

Figure S34: left: ^{1}H NMR spectra of all sequences from Class 17.180. All spectra were measured with 1024 scans. The spectrum of sequence 17.72 was measured after weeks on the bench. Right: ion exchange chromatographs of two representative sequences chosen for characterization in the secondary screen.

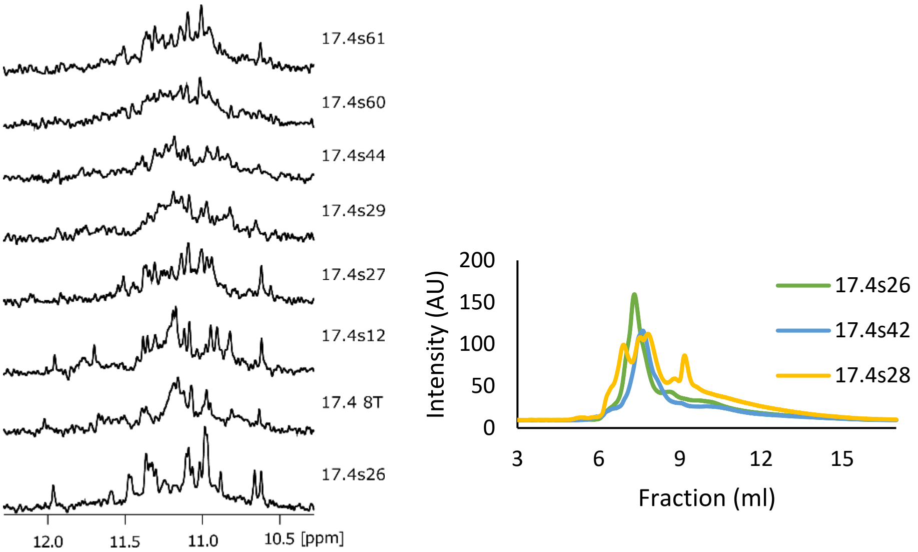

Figure S35: left: ^{1}H NMR spectra of randomly chosen representatives of Class 17.4s26. Right: ion exchange chromatographs of three representative sequences chosen for characterization in the secondary screen.

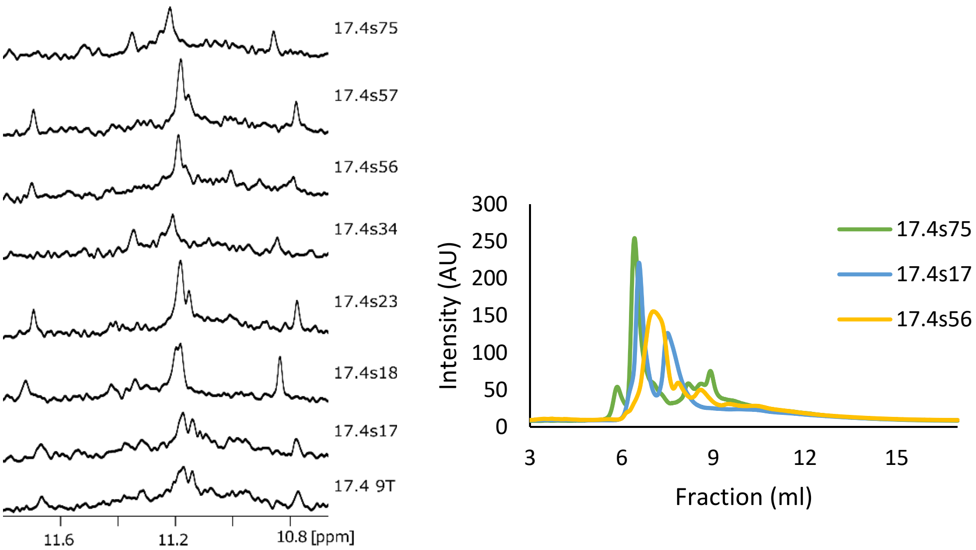

Figure S36: left:^{1}H NMR spectra of all sequences from Class 17.4+17.4s26. Right: ion exchange chromatographs of three representative sequences chosen for characterization in the secondary screen.

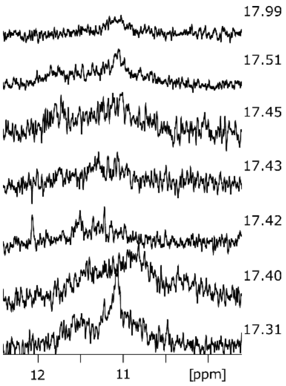

Figure S37: ^{1}H NMR spectra of all sequences from Class "no clear pattern." Spectra of sequences 17.31, 17.40, and 17.43 were measured with 1024 scans after weeks on the bench, the spectrum of sequence 17.42 was measured using a sample from

the second preparation with a DNA concentration of 50 µM with 512 scans, the spectrum of sequence 17.45 was measured using a sample from the second preparation with a DNA concertation of 50 µM with 1024 scans, and the spectrum of sequence 17.99 was measured after weeks on the bench.

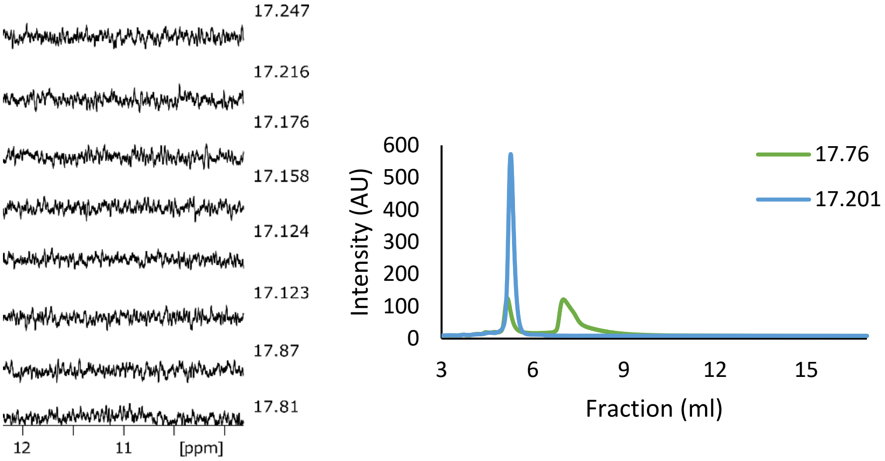

Figure S38: left: ^{1}H NMR spectra of randomly chosen representatives of Class "no G-quadruplex signals." Right: ion exchange chromatographs of two representative sequences chosen for characterization in the secondary screen.

Section SI_table

Table S4: List of all sequences

Table S4: List of all sequences, their names used in this study, their names used in a previous study [19], the classes they belong to, and parameters of ^{1}H NMR spectra chosen as representative for a given sequence (all spectra are shown in the Section SI_spectra). The column labeled "Class" contains all classes to which given sequence belongs (always one major class and up to two subclasses). Classes names are the same as in Table S1. The last four columns contain parameters of representative NMR spectra. The column "NS" indicates the number of scans, the column "Prep." contains "1" if spectra were measured using samples from the first preparation and "2" if spectra were measured using samples from the second preparation, the column "Time" contains "P" for spectra measured within 3 days after preparation and "W" for spectra measured after weeks on the bench, and the column "c (μM)" indicates the DNA concentration of the sample used for the measurement.

Name	Name in [19]	Sequence	Class
17.3	A1	GGGTGGGAAGGGTGGGA	17. 3, 17.3a
17.4	A2	GAGTGGGAAGGGTGGGA	17.4, 17.4a, 17.4c
17.5	A3	GCGTGGGAAGGGTGGGA	17.4, 17.4a, 17.4c
17.6	A4	GTGTGGGAAGGGTGGGA	17.4, 17.4a, 17.4c
17.7	A5	GGGTGAGAAGGGTGGGA	17.4, 17.4b, 17.4c
17.8	A6	GGGTGCGAAGGGTGGGA	17.4, 17.4b, 17.4c
17.9	A7	GGGTGTGAAGGGTGGGA	17.4, 17.4b, 17.4c
17.10	A8	GGGTGGGAAGAGTGGGA	17. 63, 17.63a, 17.63d
17.11	A9	GGGTGGGAAGCGTGGGA	17. 63, 17.63a, 17.63d
17.12	A10	GGGTGGGAAGTGTGGGA	17. 63, 17.63a, 17.63d
17.13	A11	GGGTGGGAAGGGTGAGA	17. 63, 17.63a, 17.63d
17.14	A12	GGGTGGGAAGGGTGCGA	17. 63, 17.63a, 17.63d
17.15	A13	GGGTGGGAAGGGTGTGA	17. 63, 17.63a, 17.63d
17.18	A14	GAGTGAGAAGGGTGGGA	17.4, 17.4a, 17.4c
17.19	A15	GAGTGCGAAGGGTGGGA	17.4, 17.4a, 17.4c
17.20	A16	GAGTGTGAAGGGTGGGA	17.4, 17.4a, 17.4c
17.21	A17	GCGTGAGAAGGGTGGGA	17.4, 17.4a, 17.4c
17.22	A18	GCGTGCGAAGGGTGGGA	17.4, 17.4a, 17.4c
17.23	A19	GCGTGTGAAGGGTGGGA	17.4, 17.4a, 17.4c
17.24	A20	GTGTGAGAAGGGTGGGA	17.4, 17.4a, 17.4c
17.25	A21	GTGTGCGAAGGGTGGGA	17.4, 17.4a, 17.4c
17.26	A22	GTGTGTGAAGGGTGGGA	17.4, 17.4a, 17.4c
17.27	A23	GAGTGGGAAGAGTGGGA	17.28
17.28	A24	GAGTGGGAAGCGTGGGA	17.28
17.29	A25	GAGTGGGAAGTGTGGGA	17.28
17.30	A26	GCGTGGGAAGAGTGGGA	17.28
17.31	A27	GCGTGGGAAGCGTGGGA	No clear pattern
17.32	A28	GCGTGGGAAGTGTGGGA	17.28
17.33	A29	GTGTGGGAAGAGTGGGA	17.28
17.34	A30	GTGTGGGAAGCGTGGGA	17.28
17.35	A31	GTGTGGGAAGTGTGGGA	17.28
17.36	B1	GAGTGGGAAGGGTGAGA	17.36
17.37	B2	GAGTGGGAAGGGTGCGA	17.36
17.38	B3	GAGTGGGAAGGGTGTGA	17.36
17.39	B4	GCGTGGGAAGGGTGAGA	17.36
17.40	B5	GCGTGGGAAGGGTGCGA	No clear pattern
17.41	B6	GCGTGGGAAGGGTGTGA	17.36
17.42	B7	GTGTGGGAAGGGTGAGA	No clear pattern
17.43	B8	GTGTGGGAAGGGTGCGA	No clear pattern
17.44	B9	GTGTGGGAAGGGTGTGA	17.36
17.45	B10	GGGTGAGAAGAGTGGGA	No clear pattern
17.46	B11	GGGTGAGAAGCGTGGGA	17.49
17.47	B12	GGGTGAGAAGTGTGGGA	17.49

17.48	B13	GGGTGCGAAGAGTGGGA	17.49
17.49	B14	GGGTGCGAAGCGTGGGA	17.49
17.50	B15	GGGTGCGAAGTGTGGGA	17.49
17.51	B16	GGGTGTGAAGAGTGGGA	No clear pattern
17.52	B17	GGGTGTGAAGCGTGGGA	17.49
17.53	B18	GGGTGTGAAGTGTGGGA	17.36
17.54	B19	GGGTGAGAAGGGTGAGA	17.36
17.55	B20	GGGTGAGAAGGGTGCGA	17.36
17.56	B21	GGGTGAGAAGGGTGTGA	17.36
17.57	B22	GGGTGCGAAGGGTGAGA	17.36
17.58	B23	GGGTGCGAAGGGTGCGA	17.36
17.59	B24	GGGTGCGAAGGGTGTGA	17.36
17.60	B25	GGGTGTGAAGGGTGAGA	17.36
17.61	B26	GGGTGTGAAGGGTGCGA	17.36
17.62	B27	GGGTGTGAAGGGTGTGA	17.36
17.63	B28	GGGTGGGAAGAGTGAGA	17. 63, 17.63b, 17.63d
17.64	B29	GGGTGGGAAGAGTGCGA	17. 63, 17.63b, 17.63d
17.65	B30	GGGTGGGAAGAGTGTGA	17. 63, 17.63b, 17.63d
17.66	B31	GGGTGGGAAGCGTGAGA	17. 63, 17.63b, 17.63d
17.67	C1	GGGTGGGAAGCGTGCGA	17. 63, 17.63b, 17.63d
17.68	C2	GGGTGGGAAGCGTGTGA	17. 63, 17.63b, 17.63d
17.69	C3	GGGTGGGAAGTGTGAGA	17. 63, 17.63b, 17.63d
17.70	C4	GGGTGGGAAGTGTGCGA	17. 63, 17.63b, 17.63d
17.71	C5	GGGTGGGAAGTGTGTGA	17. 63, 17.63b, 17.63d
17.72	C6	GGGTGAGAAGAGTGAGA	17.180
17.73	C7	GGGTGAGAAGAGTGCGA	17.118
17.74	C8	GGGTGAGAAGAGTGTGA	No G4 signals
17.75	C9	GGGTGAGAAGTGTGAGA	No G4 signals
17.76	C10	GGGTGAGAAGTGTGCGA	No G4 signals
17.77	C11	GGGTGAGAAGTGTGTGA	17.118
17.78	C12	GGGTGAGAAGCGTGAGA	No G4 signals
17.79	C13	GGGTGAGAAGCGTGCGA	No G4 signals
17.80	C14	GGGTGAGAAGCGTGTGA	No G4 signals
17.81	C15	GGGTGCGAAGAGTGAGA	No G4 signals
17.82	C16	GGGTGCGAAGAGTGCGA	17.118
17.83	C17	GGGTGCGAAGAGTGTGA	No G4 signals
17.84	C18	GGGTGCGAAGTGTGAGA	No G4 signals
17.85	C19	GGGTGCGAAGTGTGCGA	No G4 signals
17.86	C20	GGGTGCGAAGTGTGTGA	No G4 signals
17.87	C21	GGGTGCGAAGCGTGAGA	No G4 signals
17.88	C22	GGGTGCGAAGCGTGCGA	No G4 signals
17.89	C23	GGGTGCGAAGCGTGTGA	No G4 signals
17.90	C24	GGGTGTGAAGAGTGAGA	17.118
17.91	C25	GGGTGTGAAGAGTGCGA	17.118
17.92	C26	GGGTGTGAAGAGTGTGA	17.118
17.93	C27	GGGTGTGAAGTGTGAGA	17.118
17.94	C28	GGGTGTGAAGTGTGCGA	17.118
17.95	C29	GGGTGTGAAGTGTGTGA	No G4 signals
17.96	C30	GGGTGTGAAGCGTGAGA	17.118
17.97	C31	GGGTGTGAAGCGTGCGA	17.118
17.98	D1	GGGTGTGAAGCGTGTGA	No G4 signals
17.99	D2	GAGTGGGAAGAGTGAGA	No G4 signals
17.100	D3	GAGTGGGAAGAGTGCGA	17.118
17.101	D4	GAGTGGGAAGAGTGTGA	No G4 signals
17.102	D5	GAGTGGGAAGTGTGAGA	No G4 signals
17.103	D6	GAGTGGGAAGTGTGCGA	No G4 signals
17.104	D7	GAGTGGGAAGTGTGTGA	No G4 signals
17.105	D8	GAGTGGGAAGCGTGAGA	No G4 signals

17.106	D9	GAGTGGGAAGCGTGCGA	No G4 signals
17.107	D10	GAGTGGGAAGCGTGTGA	No G4 signals
17.108	D11	GCGTGGGAAGAGTGAGA	No G4 signals
17.109	D12	GCGTGGGAAGAGTGCGA	17.109
17.110	D13	GCGTGGGAAGAGTGTGA	No clear pattern
17.111	D14	GCGTGGGAAGTGTGAGA	No G4 signals
17.112	D15	GCGTGGGAAGTGTGCGA	No G4 signals
17.113	D16	GCGTGGGAAGTGTGTGA	No G4 signals
17.114	D17	GCGTGGGAAGCGTGAGA	No G4 signals
17.115	D18	GCGTGGGAAGCGTGCGA	No G4 signals
17.116	D19	GCGTGGGAAGCGTGTGA	No G4 signals
17.117	D20	GTGTGGGAAGAGTGAGA	No G4 signals
17.118	D21	GTGTGGGAAGAGTGCGA	17.118
17.119	D22	GTGTGGGAAGAGTGTGA	No G4 signals
17.120	D23	GTGTGGGAAGTGTGAGA	No G4 signals
17.121	D24	GTGTGGGAAGTGTGCGA	No G4 signals
17.122	D25	GTGTGGGAAGTGTGTGA	No G4 signals
17.123	D26	GTGTGGGAAGCGTGAGA	No G4 signals
17.124	D27	GTGTGGGAAGCGTGCGA	No G4 signals
17.125	D28	GTGTGGGAAGCGTGTGA	No G4 signals
17.126	D29	GAGTGAGAAGGGTGAGA	No G4 signals
17.127	D30	GAGTGAGAAGGGTGCGA	No G4 signals
17.128	D31	GAGTGAGAAGGGTGTGA	No G4 signals
17.129	E1	GAGTGTGAAGGGTGAGA	No G4 signals
17.130	E2	GAGTGTGAAGGGTGCGA	No G4 signals
17.131	E3	GAGTGTGAAGGGTGTGA	No G4 signals
17.132	E4	GAGTGCGAAGGGTGAGA	No G4 signals
17.133	E5	GAGTGCGAAGGGTGCGA	No G4 signals
17.134	E6	GAGTGCGAAGGGTGTGA	No G4 signals
17.135	E7	GCGTGAGAAGGGTGAGA	No G4 signals
17.136	E8	GCGTGAGAAGGGTGCGA	No G4 signals
17.137	E9	GCGTGAGAAGGGTGTGA	No G4 signals
17.138	E10	GCGTGTGAAGGGTGAGA	No G4 signals
17.139	E11	GCGTGTGAAGGGTGCGA	No G4 signals
17.140	E12	GCGTGTGAAGGGTGTGA	No G4 signals
17.141	E13	GCGTGCGAAGGGTGAGA	No G4 signals
17.142	E14	GCGTGCGAAGGGTGCGA	No G4 signals
17.143	E15	GCGTGCGAAGGGTGTGA	No G4 signals
17.144	E16	GTGTGAGAAGGGTGAGA	No G4 signals
17.145	E17	GTGTGAGAAGGGTGCGA	No G4 signals
17.146	E18	GTGTGAGAAGGGTGTGA	No G4 signals
17.147	E19	GTGTGTGAAGGGTGAGA	No G4 signals
17.148	E20	GTGTGTGAAGGGTGCGA	No G4 signals
17.149	E21	GTGTGTGAAGGGTGTGA	No G4 signals
17.150	E22	GTGTGCGAAGGGTGAGA	No G4 signals
17.151	E23	GTGTGCGAAGGGTGCGA	No G4 signals
17.152	E24	GTGTGCGAAGGGTGTGA	No G4 signals
17.153	E25	GAGTGAGAAGAGTGGGA	17.180
17.154	E26	GAGTGAGAAGCGTGGGA	17.154
17.155	E27	GAGTGAGAAGTGTGGGA	17.154
17.156	E28	GAGTGTGAAGAGTGGGA	No G4 signals
17.157	E29	GAGTGTGAAGCGTGGGA	No G4 signals
17.158	E30	GAGTGTGAAGTGTGGGA	No G4 signals
17.159	E31	GAGTGCGAAGAGTGGGA	No G4 signals
17.160	F1	GAGTGCGAAGCGTGGGA	No G4 signals
17.161	F2	GAGTGCGAAGTGTGGGA	No G4 signals
17.162	F3	GCGTGAGAAGAGTGGGA	17.154
17.163	F4	GCGTGAGAAGCGTGGGA	No G4 signals

17.164	F5	GCGTGAGAAGTGTGGGA	17.154
17.165	F6	GCGTGTGAAGAGTGGGA	No G4 signals
17.166	F7	GCGTGTGAAGCGTGGGA	No G4 signals
17.167	F8	GCGTGTGAAGTGTGGGA	No G4 signals
17.168	F9	GCGTGCGAAGAGTGGGA	No G4 signals
17.169	F10	GCGTGCGAAGCGTGGGA	No G4 signals
17.170	F11	GCGTGCGAAGTGTGGGA	No G4 signals
17.171	F12	GTGTGAGAAGAGTGGGA	17.154
17.172	F13	GTGTGAGAAGCGTGGGA	No G4 signals
17.173	F14	GTGTGAGAAGTGTGGGA	17.154
17.174	F15	GTGTGTGAAGAGTGGGA	No G4 signals
17.175	F16	GTGTGTGAAGCGTGGGA	No G4 signals
17.176	F17	GTGTGTGAAGTGTGGGA	17.154
17.177	F18	GTGTGCGAAGAGTGGGA	No G4 signals
17.178	F19	GTGTGCGAAGCGTGGGA	No G4 signals
17.179	F20	GTGTGCGAAGTGTGGGA	No G4 signals
17.180	F21	GAGTGAGAAGAGTGAGA	17.180
17.181	F22	GAGTGAGAAGAGTGCGA	17.180
17.182	F23	GAGTGAGAAGAGTGTGA	17.180
17.183	F24	GAGTGAGAAGCGTGAGA	No G4 signals
17.184	F25	GAGTGAGAAGCGTGCGA	No G4 signals
17.185	F26	GAGTGAGAAGCGTGTGA	No G4 signals
17.186	F27	GAGTGAGAAGTGTGAGA	No G4 signals
17.187	F28	GAGTGAGAAGTGTGCGA	No G4 signals
17.188	F29	GAGTGAGAAGTGTGTGA	No G4 signals
17.189	F30	GAGTGCGAAGAGTGAGA	No G4 signals
17.190	F31	GAGTGCGAAGAGTGCGA	17.118
17.191	G1	GAGTGCGAAGAGTGTGA	No G4 signals
17.192	G2	GAGTGCGAAGCGTGAGA	No G4 signals
17.193	G3	GAGTGCGAAGCGTGCGA	No G4 signals
17.194	G4	GAGTGCGAAGCGTGTGA	No G4 signals
17.195	G5	GAGTGCGAAGTGTGAGA	No G4 signals
17.196	G6	GAGTGCGAAGTGTGCGA	No G4 signals
17.197	G7	GAGTGCGAAGTGTGTGA	No G4 signals
17.198	G8	GAGTGTGAAGAGTGAGA	No G4 signals
17.199	G9	GAGTGTGAAGAGTGCGA	17.118
17.200	G10	GAGTGTGAAGAGTGTGA	No G4 signals
17.201	G11	GAGTGTGAAGCGTGAGA	No G4 signals
17.202	G12	GAGTGTGAAGCGTGCGA	No G4 signals
17.203	G13	GAGTGTGAAGCGTGTGA	No G4 signals
17.204	G14	GAGTGTGAAGTGTGAGA	No G4 signals
17.205	G15	GAGTGTGAAGTGTGCGA	No G4 signals
17.206	G16	GAGTGTGAAGTGTGTGA	No G4 signals
17.207	G17	GCGTGAGAAGAGTGAGA	17.180
17.208	G18	GCGTGAGAAGAGTGCGA	17.118
17.209	G19	GCGTGAGAAGAGTGTGA	No G4 signals
17.210	G20	GCGTGAGAAGCGTGAGA	No G4 signals
17.211	G21	GCGTGAGAAGCGTGCGA	No G4 signals
17.212	G22	GCGTGAGAAGCGTGTGA	No G4 signals
17.213	G23	GCGTGAGAAGTGTGAGA	No G4 signals
17.214	G24	GCGTGAGAAGTGTGCGA	No G4 signals
17.215	G25	GCGTGAGAAGTGTGTGA	No G4 signals
17.216	G26	GCGTGCGAAGAGTGAGA	No G4 signals
17.217	G27	GCGTGCGAAGAGTGCGA	17.154
17.218	G28	GCGTGCGAAGAGTGTGA	No G4 signals
17.219	G29	GCGTGCGAAGCGTGAGA	No G4 signals
17.220	G30	GCGTGCGAAGCGTGCGA	No G4 signals
17.221	G31	GCGTGCGAAGCGTGTGA	No G4 signals

17.222	H1	GCGTGCGAAGTGTGAGA	No G4 signals
17.223	H2	GCGTGCGAAGTGTGCGA	No G4 signals
17.224	H3	GCGTGCGAAGTGTGTGA	No G4 signals
17.225	H4	GCGTGTGAAGAGTGAGA	No G4 signals
17.226	H5	GCGTGTGAAGAGTGCGA	17.118
17.227	H6	GCGTGTGAAGAGTGTGA	No G4 signals
17.228	H7	GCGTGTGAAGCGTGAGA	No G4 signals
17.229	H8	GCGTGTGAAGCGTGCGA	No G4 signals
17.230	H9	GCGTGTGAAGCGTGTGA	No G4 signals
17.231	H10	GCGTGTGAAGTGTGAGA	No G4 signals
17.232	H11	GCGTGTGAAGTGTGCGA	No G4 signals
17.233	H12	GCGTGTGAAGTGTGTGA	No G4 signals
17.234	H13	GTGTGAGAAGAGTGAGA	17.180
17.235	H14	GTGTGAGAAGAGTGCGA	17.118
17.236	H15	GTGTGAGAAGAGTGTGA	No G4 signals
17.237	H16	GTGTGAGAAGCGTGAGA	No G4 signals
17.238	H17	GTGTGAGAAGCGTGCGA	No G4 signals
17.239	H18	GTGTGAGAAGCGTGTGA	No G4 signals
17.240	H19	GTGTGAGAAGTGTGAGA	No G4 signals
17.241	H20	GTGTGAGAAGTGTGCGA	No G4 signals
17.242	H21	GTGTGAGAAGTGTGTGA	No G4 signals
17.243	H22	GTGTGCGAAGAGTGAGA	No G4 signals
17.244	H23	GTGTGCGAAGAGTGCGA	No G4 signals
17.245	H24	GTGTGCGAAGAGTGTGA	No G4 signals
17.246	H25	GTGTGCGAAGCGTGAGA	No G4 signals
17.247	H26	GTGTGCGAAGCGTGCGA	No G4 signals
17.248	H27	GTGTGCGAAGCGTGTGA	No G4 signals
17.249	H28	GTGTGCGAAGTGTGAGA	No G4 signals
17.250	H29	GTGTGCGAAGTGTGCGA	No G4 signals
17.251	H30	GTGTGCGAAGTGTGTGA	No G4 signals
17.252	H31	GTGTGTGAAGAGTGAGA	No G4 signals
17.253	I1	GTGTGTGAAGAGTGCGA	17.118
17.254	I2	GTGTGTGAAGAGTGTGA	No G4 signals
17.255	I3	GTGTGTGAAGCGTGAGA	No G4 signals
17.256	I4	GTGTGTGAAGCGTGCGA	No G4 signals
17.257	I5	GTGTGTGAAGCGTGTGA	No G4 signals
17.258	I6	GTGTGTGAAGTGTGAGA	No G4 signals
17.259	I7	GTGTGTGAAGTGTGCGA	No G4 signals
17.260	I8	GTGTGTGAAGTGTGTGA	No G4 signals
17.3 4A	I9	GGGAGGGAAGGGTGGGA	17.3, 17.3a
17.3 4C	I10	GGGCGGGAAGGGTGGGA	17.3, 17.3a
17.3 8C	I11	GGGTGGGCAGGGTGGGA	17.3, 17.3c
17.3 8T	I12	GGGTGGGTAGGGTGGGA	17.3, 17.3c
17.3 9C	I13	GGGTGGGACGGGTGGGA	17.3, 17.3a
17.3 9T	I14	GGGTGGGATGGGTGGGA	17.3, 17.3a, 17.3b
17.3 13A	I15	GGGTGGGAAGGGAGGGA	17.3, 17.3a
17.3 13C	I16	GGGTGGGAAGGGCGGGA	17.3, 17.3a
17.3s10	I17	GGGAGGGCAGGGTGGGA	17.3, 17.3c
17.3s11	I18	GGGAGGGTAGGGTGGGA	17.3, 17.3c
17.3s12	I19	GGGCGGGCAGGGTGGGA	17.3, 17.3c
17.3s13	I20	GGGCGGGTAGGGTGGGA	17.3, 17.3c
17.3s14	I21	GGGTGGGACGGGAGGGA	17.3, 17.3a
17.3s15	I22	GGGTGGGACGGGCGGGA	17.3, 17.3a
17.3s16	I23	GGGTGGGATGGGAGGGA	17.3, 17.3a, 17.3b
17.3s17	I24	GGGTGGGATGGGCGGGA	17.3, 17.3a, 17.3b
17.3s18	I25	GGGAGGGACGGGTGGGA	17.3, 17.3a
17.3s19	I26	GGGAGGGATGGGTGGGA	17.3, 17.3a, 17.3b
17.3s20	I27	GGGAGGGAAGGGAGGGA	17.3, 17.3a

17.3s21	I28	GGGAGGGAAGGGCGGGA	17.3, 17.3a
17.3s22	I29	GGGCGGGACGGGTGGGA	17.3, 17.3a
17.3s23	I30	GGGCGGGATGGGTGGGA	17.3, 17.3a, 17.3b
17.3s24	I31	GGGCGGGAAGGGAGGGA	17.3, 17.3a
17.3s25	J1	GGGCGGGAAGGGCGGGA	17.3, 17.3a
17.3s26	J2	GGGTGGGCCGGGTGGGA	17.3, 17.3d
17.3s27	J3	GGGTGGGCTGGGTGGGA	17.3, 17.3b, 17.3d
17.3s28	J4	GGGTGGGCAGGGAGGGA	17.3, 17.3c
17.3s29	J5	GGGTGGGCAGGGCGGGA	17.3, 17.3c
17.3s30	J6	GGGTGGGTCGGGTGGGA	17.3, 17.3d
17.3s31	J7	GGGTGGGTTGGGTGGGA	17.3, 17.3b, 17.3d
17.3s32	J8	GGGTGGGTAGGGAGGGA	17.3, 17.3c
17.3s33	J9	GGGTGGGTAGGGCGGGA	17.3, 17.3c
17.3s34	J10	GGGAGGGCCGGGTGGGA	17.3, 17.3d
17.3s35	J11	GGGAGGGCTGGGTGGGA	17.3, 17.3b, 17.3d
17.3s36	J12	GGGAGGGCAGGGAGGGA	17.3, 17.3c
17.3s37	J13	GGGAGGGCAGGGCGGGA	17.3, 17.3c
17.3s38	J14	GGGAGGGTCGGGTGGGA	17.3, 17.3d
17.3s39	J15	GGGAGGGTTGGGTGGGA	17.3, 17.3b, 17.3d
17.3s40	J16	GGGAGGGTAGGGAGGGA	17.3, 17.3c
17.3s41	J17	GGGAGGGTAGGGCGGGA	17.3, 17.3c
17.3s42	J18	GGGCGGGCCGGGTGGGA	17.3, 17.3d
17.3s43	J19	GGGCGGGCTGGGTGGGA	17.3, 17.3b, 17.3d
17.3s44	J20	GGGCGGGCAGGGAGGGA	17.3, 17.3c
17.3s45	J21	GGGCGGGCAGGGCGGGA	17.3, 17.3c
17.3s46	J22	GGGCGGGTCGGGTGGGA	17.3, 17.3d
17.3s47	J23	GGGCGGGTTGGGTGGGA	17.3, 17.3b, 17.3d
17.3s48	J24	GGGCGGGTAGGGAGGGA	17.3, 17.3c
17.3s49	J25	GGGCGGGTAGGGCGGGA	17.3, 17.3c
17.3s50	J26	GGGAGGGACGGGAGGGA	17.3, 17.3a
17.3s51	J27	GGGAGGGACGGGCGGGA	17.3, 17.3a
17.3s52	J28	GGGAGGGATGGGAGGGA	17.3, 17.3a, 17.3b
17.3s53	J29	GGGAGGGATGGGCGGGA	17.3, 17.3a, 17.3b
17.3s54	J30	GGGCGGGACGGGAGGGA	17.3, 17.3a
17.3s55	J31	GGGCGGGACGGGCGGGA	17.3, 17.3a
17.3s56	K1	GGGCGGGATGGGAGGGA	17.3, 17.3a, 17.3b
17.3s57	K2	GGGCGGGATGGGCGGGA	17.3, 17.3a, 17.3b
17.3s58	K3	GGGTGGGCCGGGAGGGA	17.3, 17.3d
17.3s59	K4	GGGTGGGCCGGGCGGGA	17.3, 17.3d
17.3s60	K5	GGGTGGGCTGGGAGGGA	17.3, 17.3b, 17.3d
17.3s61	K6	GGGTGGGCTGGGCGGGA	17.3, 17.3b, 17.3d
17.3s62	K7	GGGTGGGTCGGGAGGGA	17.3, 17.3d
17.3s63	K8	GGGTGGGTCGGGCGGGA	17.3, 17.3d
17.3s64	K9	GGGTGGGTTGGGAGGGA	17.3, 17.3b, 17.3d
17.3s65	K10	GGGTGGGTTGGGCGGGA	17.3, 17.3b, 17.3d
17.3s66	K11	GGGCGGGTCGGGAGGGA	17.3, 17.3d
17.3s67	K12	GGGCGGGTCGGGCGGGA	17.3, 17.3d
17.3s68	K13	GGGCGGGTTGGGAGGGA	17.3, 17.3b, 17.3d
17.3s69	K14	GGGCGGGTTGGGCGGGA	17.3, 17.3b, 17.3d
17.3s70	K15	GGGCGGGCCGGGAGGGA	17.3, 17.3d
17.3s71	K16	GGGCGGGCCGGGCGGGA	17.3, 17.3d
17.3s72	K17	GGGCGGGCTGGGAGGGA	17.3, 17.3b, 17.3d
17.3s73	K18	GGGCGGGCTGGGCGGGA	17.3, 17.3b, 17.3d
17.3s74	K19	GGGAGGGCCGGGAGGGA	17.3, 17.3d
17.3s75	K20	GGGAGGGCCGGGCGGGA	17.3, 17.3d
17.3s76	K21	GGGAGGGCTGGGAGGGA	17.3, 17.3b, 17.3d
17.3s77	K22	GGGAGGGCTGGGCGGGA	17.3, 17.3b, 17.3d
17.3s78	K23	GGGAGGGTCGGGAGGGA	17.3, 17.3d

17.3s79	K24	GGGAGGGTCGGGCGGGA	17.3, 17.3d
17.3s80	K25	GGGAGGGTTGGGAGGGA	17.3, 17.3b, 17.3d
17.3s81	K26	GGGAGGGTTGGGCGGGA	17.3, 17.3b, 17.3d
17.4 4A	K27	GAGAGGGAAGGGTGGGA	17.4, 17.4a, 17.4c
17.4 4C	K28	GAGCGGGAAGGGTGGGA	17.4, 17.4a, 17.4c
17.4 8C	K29	GAGTGGGCAGGGTGGGA	17.4s26
17.4 8T	K30	GAGTGGGTAGGGTGGGA	17.4s26
17.4 9C	K31	GAGTGGGACGGGTGGGA	17.4s26
17.4 9T	L1	GAGTGGGATGGGTGGGA	17.4 + 17.4s26
17.4 13A	L2	GAGTGGGAAGGGAGGGA	17.4, 17.4a, 17.4c
17.4 13C	L3	GAGTGGGAAGGGCGGGA	17.4, 17.4a, 17.4c
17.4s10	L4	GAGAGGGCAGGGTGGGA	17.4, 17.4a, 17.4d
17.4s11	L5	GAGAGGGTAGGGTGGGA	17.4, 17.4a, 17.4d
17.4s12	L6	GAGCGGGCAGGGTGGGA	17.4s26
17.4s13	L7	GAGCGGGTAGGGTGGGA	17.4s26
17.4s14	L8	GAGTGGGACGGGAGGGA	17.4s26
17.4s15	L9	GAGTGGGACGGGCGGGA	17.4s26
17.4s16	L10	GAGTGGGATGGGAGGGA	17.4s26
17.4s17	L11	GAGTGGGATGGGCGGGA	17.4 + 17.4s26
17.4s18	L12	GAGAGGGACGGGTGGGA	17.4 + 17.4s26
17.4s19	L13	GAGAGGGATGGGTGGGA	17.4, 17.4a, 17.4d
17.4s20	L14	GAGAGGGAAGGGAGGGA	17.4, 17.4a, 17.4c
17.4s21	L15	GAGAGGGAAGGGCGGGA	17.4, 17.4a, 17.4c
17.4s22	L16	GAGCGGGACGGGTGGGA	17.4s26
17.4s23	L17	GAGCGGGATGGGTGGGA	17.4 + 17.4s26
17.4s24	L18	GAGCGGGAAGGGAGGGA	17.4, 17.4a, 17.4c
17.4s25	L19	GAGCGGGAAGGGCGGGA	17.4, 17.4a, 17.4c
17.4s26	L20	GAGTGGGCCGGGTGGGA	17.4s26
17.4s27	L21	GAGTGGGCTGGGTGGGA	17.4s26
17.4s28	L22	GAGTGGGCAGGGAGGGA	17.4s26
17.4s29	L23	GAGTGGGCAGGGCGGGA	17.4s26
17.4s30	L24	GAGTGGGTCGGGTGGGA	17.4s26
17.4s31	L25	GAGTGGGTTGGGTGGGA	17.4s26
17.4s32	L26	GAGTGGGTAGGGAGGGA	17.4s26
17.4s33	L27	GAGTGGGTAGGGCGGGA	17.4s26
17.4s34	L28	GAGAGGGCCGGGTGGGA	17.4 + 17.4s26
17.4s35	L29	GAGAGGGCTGGGTGGGA	17.4s26
17.4s36	L30	GAGAGGGCAGGGAGGGA	17.4, 17.4a, 17.4d
17.4s37	L31	GAGAGGGCAGGGCGGGA	17.4, 17.4a, 17.4d
17.4s38	M1	GAGAGGGTCGGGTGGGA	17.4s26
17.4s39	M2	GAGAGGGTTGGGTGGGA	17.4s26
17.4s40	M3	GAGAGGGTAGGGAGGGA	17.4, 17.4a, 17.4d
17.4s41	M4	GAGAGGGTAGGGCGGGA	17.4, 17.4a, 17.4d
17.4s42	M5	GAGCGGGCCGGGTGGGA	17.4s26
17.4s43	M6	GAGCGGGCTGGGTGGGA	17.4s26
17.4s44	M7	GAGCGGGCAGGGAGGGA	17.4s26
17.4s45	M8	GAGCGGGCAGGGCGGGA	17.4s26
17.4s46	M9	GAGCGGGTCGGGTGGGA	17.4s26
17.4s47	M10	GAGCGGGTTGGGTGGGA	17.4s26
17.4s48	M11	GAGCGGGTAGGGAGGGA	17.4s26
17.4s49	M12	GAGCGGGTAGGGCGGGA	17.4s26
17.4s50	M13	GAGAGGGACGGGAGGGA	17.4, 17.4a, 17.4d
17.4s51	M14	GAGAGGGACGGGCGGGA	17.4, 17.4a, 17.4d
17.4s52	M15	GAGAGGGATGGGAGGGA	17.4, 17.4a, 17.4d
17.4s53	M16	GAGAGGGATGGGCGGGA	17.4, 17.4a, 17.4d
17.4s54	M17	GAGCGGGACGGGAGGGA	17.4s26
17.4s55	M18	GAGCGGGACGGGCGGGA	17.4s26
17.4s56	M19	GAGCGGGATGGGAGGGA	17.4 + 17.4s26

17.4s57	M20	GAGCGGGATGGCGGGA	17.4 + 17.4s26
17.4s58	M21	GAGTGGGCCGGGAGGGA	17.4s26
17.4s59	M22	GAGTGGGCCGGGCGGGA	17.4s26
17.4s60	M23	GAGTGGGCTGGGAGGGA	17.4s26
17.4s61	M24	GAGTGGGCTGGGCGGGA	17.4s26
17.4s62	M25	GAGTGGGTCGGGAGGGA	17.4s26
17.4s63	M26	GAGTGGGTCGGGCGGGA	17.4s26
17.4s64	M27	GAGTGGGTTGGGAGGGA	17.4s26
17.4s65	M28	GAGTGGGTTGGGCGGGA	17.4s26
17.4s66	M29	GAGCGGGTCGGGAGGGA	17.4s26
17.4s67	M30	GAGCGGGTCGGGCGGGA	17.4s26
17.4s68	M31	GAGCGGGTTGGGAGGGA	17.4s26
17.4s69	N1	GAGCGGGTTGGGCGGGA	17.4s26
17.4s70	N2	GAGCGGGCCGGGAGGGA	17.4s26
17.4s71	N3	GAGCGGGCCGGGCGGGA	17.4s26
17.4s72	N4	GAGCGGGCTGGGAGGGA	17.4s26
17.4s73	N5	GAGCGGGCTGGGCGGGA	17.4s26
17.4s74	N6	GAGAGGGCCGGGAGGGA	17.4s26
17.4s75	N7	GAGAGGGCCGGGCGGGA	17.4 + 17.4s26
17.4s76	N8	GAGAGGGCTGGGAGGGA	17.4s26
17.4s77	N9	GAGAGGGCTGGGCGGGA	17.4s26
17.4s78	N10	GAGAGGGTCGGGAGGGA	17.4s26
17.4s79	N11	GAGAGGGTCGGGCGGGA	17.4s26
17.4s80	N12	GAGAGGGTTGGGAGGGA	17.4s26
17.4s81	N13	GAGAGGGTTGGGCGGGA	17.4s26
17.10 4A	N14	GGGAGGGAAGAGTGGGA	17.63, 17.63a, 17.63d
17.10 4C	N15	GGGCGGGAAGAGTGGGA	17.63, 17.63a, 17.63d
17.10 8C	N16	GGGTGGGCAGAGTGGGA	17.63, 17.63a, 17.63d
17.10 8T	N17	GGGTGGGTAGAGTGGGA	17.63, 17.63a, 17.63d
17.10 9C	N18	GGGTGGGACGAGTGGGA	17.63, 17.63a, 17.63c
17.10 9T	N19	GGGTGGGATGAGTGGGA	17.63, 17.63a, 17.63c
17.10 13A	N20	GGGTGGGAAGAGAGGGA	17.63, 17.63a, 17.63d
17.10 13C	N21	GGGTGGGAAGAGCGGGA	17.63, 17.63a, 17.63d
17.10s10	N22	GGGAGGGCAGAGTGGGA	17.63, 17.63a, 17.63d
17.10s11	N23	GGGAGGGTAGAGTGGGA	17.63, 17.63a, 17.63d
17.10s12	N24	GGGCGGGCAGAGTGGGA	17.63, 17.63a, 17.63d
17.10s13	N25	GGGCGGGTAGAGTGGGA	17.63, 17.63a, 17.63d
17.10s14	N26	GGGTGGGACGAGAGGGA	17.63, 17.63a, 17.63c
17.10s15	N27	GGGTGGGACGAGCGGGA	17.63, 17.63a, 17.63d
17.10s16	N28	GGGTGGGATGAGAGGGA	17.63, 17.63a, 17.63c
17.10s17	N29	GGGTGGGATGAGCGGGA	17.63, 17.63a, 17.63d
17.10s18	N30	GGGAGGGACGAGTGGGA	17.63, 17.63a, 17.63d
17.10s19	N31	GGGAGGGATGAGTGGGA	17.63, 17.63a, 17.63d
17.10s20	O1	GGGAGGGAAGAGAGGGA	17.63, 17.63a, 17.63d
17.10s21	O2	GGGAGGGAAGAGCGGGA	17.63, 17.63a, 17.63d
17.10s22	O3	GGGCGGGACGAGTGGGA	17.63, 17.63a, 17.63c
17.10s23	O4	GGGCGGGATGAGTGGGA	17.63, 17.63a, 17.63c
17.10s24	O5	GGGCGGGAAGAGAGGGA	17.63, 17.63a, 17.63d
17.10s25	O6	GGGCGGGAAGAGCGGGA	17.63, 17.63a, 17.63d
17.10s26	O7	GGGTGGGCCGAGTGGGA	17.63, 17.63a, 17.63d
17.10s27	O8	GGGTGGGCTGAGTGGGA	17.63, 17.63a, 17.63d
17.10s28	O9	GGGTGGGCAGAGAGGGA	17.63, 17.63a, 17.63d
17.10s29	O10	GGGTGGGCAGAGCGGGA	17.63, 17.63a, 17.63d
17.10s30	O11	GGGTGGGTCGAGTGGGA	17.63, 17.63a, 17.63d
17.10s31	O12	GGGTGGGTTGAGTGGGA	17.63, 17.63a, 17.63d
17.10s32	O13	GGGTGGGTAGAGAGGGA	17.63, 17.63a, 17.63d
17.10s33	O14	GGGTGGGTAGAGCGGGA	17.63, 17.63a, 17.63d
17.10s34	O15	GGGAGGGCCGAGTGGGA	17.63, 17.63a, 17.63d

17.10s35	O16	GGGAGGGCTGAGTGGGA	17.63, 17.63a, 17.63d
17.10s36	O17	GGGAGGGCAGAGAGGGA	17.63, 17.63a, 17.63d
17.10s37	O18	GGGAGGGCAGAGCGGGA	17.63, 17.63a, 17.63d
17.10s38	O19	GGGAGGGTCGAGTGGGA	17.63, 17.63a, 17.63d
17.10s39	O20	GGGAGGGTTGAGTGGGA	17.63, 17.63a, 17.63d
17.10s40	O21	GGGAGGGTAGAGAGGGA	17.63, 17.63a, 17.63d
17.10s41	O22	GGGAGGGTAGAGCGGGA	17.63, 17.63a, 17.63d
17.10s42	O23	GGGCGGGCCGAGTGGGA	17.63, 17.63a, 17.63d
17.10s43	O24	GGGCGGGCTGAGTGGGA	17.63, 17.63a, 17.63d
17.10s44	O25	GGGCGGGCAGAGAGGGA	17.63, 17.63a, 17.63d
17.10s45	O26	GGGCGGGCAGAGCGGGA	17.63, 17.63a, 17.63d
17.10s46	O27	GGGCGGGTCGAGTGGGA	17.63, 17.63a, 17.63d
17.10s47	O28	GGGCGGGTTGAGTGGGA	17.63, 17.63a, 17.63d
17.10s48	O29	GGGCGGGTAGAGAGGGA	17.63, 17.63a, 17.63d
17.10s49	O30	GGGCGGGTAGAGCGGGA	17.63, 17.63a, 17.63d
17.10s50	O31	GGGAGGGACGAGAGGGA	17.63, 17.63a, 17.63c
17.10s51	P1	GGGAGGGACGAGCGGGA	17.63, 17.63a, 17.63d
17.10s52	P2	GGGAGGGATGAGAGGGA	17.63, 17.63a, 17.63c
17.10s53	P3	GGGAGGGATGAGCGGGA	17.63, 17.63a, 17.63d
17.10s54	P4	GGGCGGGACGAGAGGGA	17.63, 17.63a, 17.63c
17.10s55	P5	GGGCGGGACGAGCGGGA	17.63, 17.63a, 17.63d
17.10s56	P6	GGGCGGGATGAGAGGGA	17.63, 17.63a, 17.63c
17.10s57	P7	GGGCGGGATGAGCGGGA	17.63, 17.63a, 17.63d
17.10s58	P8	GGGTGGGCCGAGAGGGA	17.63, 17.63a, 17.63d
17.10s59	P9	GGGTGGGCCGAGCGGGA	17.63, 17.63a, 17.63d
17.10s60	P10	GGGTGGGCTGAGAGGGA	17.63, 17.63a, 17.63d
17.10s61	P11	GGGTGGGCTGAGCGGGA	17.63, 17.63a, 17.63d
17.10s62	P12	GGGTGGGTCGAGAGGGA	17.63, 17.63a, 17.63d
17.10s63	P13	GGGTGGGTCGAGCGGGA	17.63, 17.63a, 17.63d
17.10s64	P14	GGGTGGGTTGAGAGGGA	17.63, 17.63a, 17.63d
17.10s65	P15	GGGTGGGTTGAGCGGGA	17.63, 17.63a, 17.63d
17.10s66	P16	GGGCGGGTCGAGAGGGA	17.63, 17.63a, 17.63d
17.10s67	P17	GGGCGGGTCGAGCGGGA	17.63, 17.63a, 17.63d
17.10s68	P18	GGGCGGGTTGAGAGGGA	17.63, 17.63a, 17.63d
17.10s69	P19	GGGCGGGTTGAGCGGGA	17.63, 17.63a, 17.63d
17.10s70	P20	GGGCGGGCCGAGAGGGA	17.63, 17.63a, 17.63d
17.10s71	P21	GGGCGGGCCGAGCGGGA	17.63, 17.63a, 17.63d
17.10s72	P22	GGGCGGGCTGAGAGGGA	17.63, 17.63a, 17.63d
17.10s73	P23	GGGCGGGCTGAGCGGGA	17.63, 17.63a, 17.63d
17.10s74	P24	GGGAGGGCCGAGAGGGA	17.63, 17.63a, 17.63d
17.10s75	P25	GGGAGGGCCGAGCGGGA	17.63, 17.63a, 17.63d
17.10s76	P26	GGGAGGGCTGAGAGGGA	17.63, 17.63a, 17.63d
17.10s77	P27	GGGAGGGCTGAGCGGGA	17.63, 17.63a, 17.63d
17.10s78	P28	GGGAGGGTCGAGAGGGA	17.63, 17.63a, 17.63d
17.10s79	P29	GGGAGGGTCGAGCGGGA	17.63, 17.63a, 17.63d
17.10s80	P30	GGGAGGGTTGAGAGGGA	17.63, 17.63a, 17.63d
17.10s81	P31	GGGAGGGTTGAGCGGGA	17.63, 17.63a, 17.63d

Section SI_clustering

Methods

For each sequence, a representative ^{1}H NMR spectrum was chosen (see Table S14 for a list and Section SI_spectra for all spectra). Spectra were then merged and trimmed to only contain the 10.3 – 12.0 ppm region, which is the part of the spectrum that contains the signals of G-quadruplex imino protons. All spectra were then scaled to a 0-1 range with a min_max scaler function (from the python software package sklearn, version 1.4.2) to avoid artifacts in results caused by different experimental settings for spectra of different sequences such as different number of scans or different DNA concentration (see Table S14 for all parameters). To reduce the number of dimensions for clustering, spectra were binned with respect to chemical shift. This was done with a qcut function (from the python software package pandas, version 2.2.2). By varying bin counts to find the right balance between detail and dimensionality reduction, we discovered that the optimal bin count in the context of our library is 200. However, we also discovered that bin count is an extremely sensitive parameter and that it needs to be determined for each version of the dataset and software. Our G-quadruplex library contained more than one hundred sequences with no signal in the trimmed region. To filter such spectra, we first established a baseline region which was defined as the region adjacent to the trimmed part of the spectra that did not appear to contain peaks (between shifts of 9.7 and 10.2 ppm). We then calculated a mean signal and standard deviation of the mean in the baseline region and then filtered out all spectra with no values greater than 3.5 standard deviations above the mean. Filtering removed 122 of the 154 spectra from the Class "No G4 signals" identified during manual sorting and no spectra from other classes. Spectra preprocessed this way were then clustered with the commonly used clustering algorithm hdbscan (from the python software package hdbscan, version 0.8.37) using default parameters. All data manipulation was done using in-house python scripts with the help of a software package pandas, version 2.2.2. These scripts are available at github: https://github.com/Jardic/nmr_clustering/tree/main.

It is important to note that the distribution of sequences into subclasses was not taken literally from the results of computer clustering. We noticed that sometimes spectra with different patterns ended up in the same cluster and some sequences with similar patterns end up in different clusters. Therefore, we did not create any classes purely based on results of computer clustering. Instead, we tried to rationalize results of clustering based on NMR spectra and created classes based on results of this analysis.

Computer clustering

To compliment and verify the results of manual clustering, we also explored computer clustering (Table S14). We analyzed the whole dataset (Tables S5 and S8) and then separately analyzed the tetrad library (Tables S6 and S9), the 17.3 loop library (Table S10), the 17.4 loop library (Tables S7 and S11), and the 17.10 loop library (Table S12). We found that computer clustering confirmed the major results of manual clustering and also showed how sequences from the three classes with the highest signal to noise ratios could be further sorted into subclasses (Table S13).

Analysis of the whole dataset revealed subclasses of Classes 17.3 and 17.4 (described below) and it also detected Class 17.118, which contains only sequences with three or four mutations in the positions that form the central tetrad in the reference G-quadruplex 17.3. Nine out of 18 sequences from this class form cluster 0 (Tables S5 and S8). Clustering of the tetrad library detected Classes 17.4 and 17.63b (the two classes in the tetrad library with highest signal to noise ratios). One of the other clusters contains a significant part of Class 17.118 and another contains a significant part of the sequences with two mutations in the central tetrad that do not belong to either Classes 17.4 or Class 17.63b.

Clustering of loop libraries revealed several subclasses which can be defined by a sequence pattern as well as minor features of ^{1}H NMR spectra (Table S13). Class 17.3 is distributed into four subclasses (Table S10) whose NMR spectra are different, but the overall pattern is still the same (Figure S39). Note that Subclass 17.3b overlaps with Classes 17.3b and 17.3d (Figure 5). All subclasses of the major Class 17.3 can be differentiated on the level of sequence. All of their properties are similar to those of the major Class 17.3 (Figures S1 and S13 – S17), and the only small difference between them is that Classes 17.3a and 17.3b bind GTP and form tetramers more efficiently than Classes 17.3c and 17.3d.

Class 17.4 can be sorted into Subclasses 17.4c and 17.4d. Note that these two subclasses are independent of Subclasses 17.4a and 17.4b and can be differentiated based on signal to noise ratios. Note also that all sequences in Class 17.4 belong to two subclasses – either 17.4a or 17.4b and either 17.4c and 17.4d (Tables S7, S11, and S13

and Figures 5 and S40). Class 17.4c contains all sequences in Class 17.4 with the loop sequence HAAH, so it contains some sequences from the 17.4 loop library and all members of Class 17.4 from the tetrad library. All properties of Subclasses 17.4c and 17.4d are similar to those of Class 17.4 (Figures S1, S18, S21, and S22)

Class 17.63 can be sorted into Subclasses 17.63a and 17.63b based on signal to noise ratios identified during manual sorting and into Subclasses 17.63c and 17.63d (Table S12 and S13, and Figure S41). Class 17.63c contains 10 out of 12 sequences from Class 17.63 with the loop sequence HAYW. The properties of sequences in Classes 17.63c and 17.63d are similar to those of sequences in Class 17.63 (Figures S1, S23, S26, and S27), while sequences in Class 17.63c bind GTP and form multimers more efficiently than those in Class 17.63d.

Figures S39 – S41: comparison of ^{1}H NMR spectra of subclasses

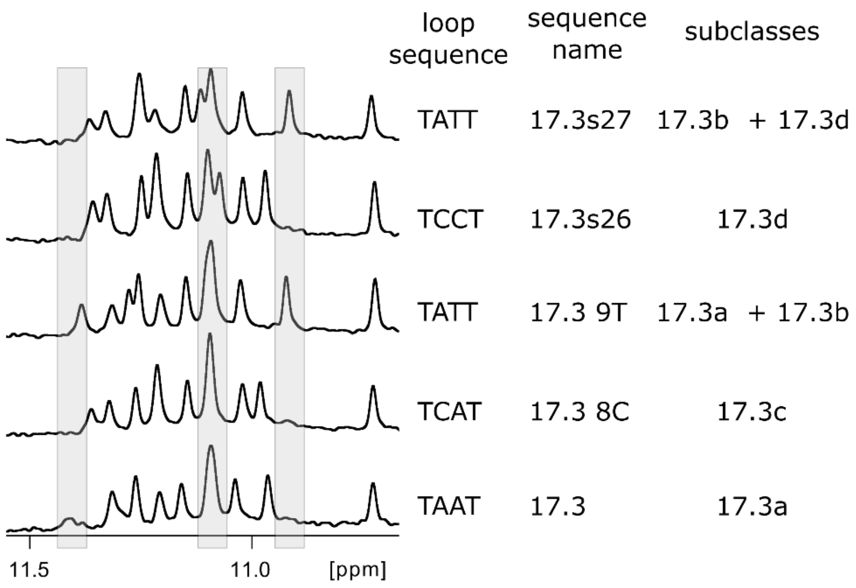

Figure S39: Subclasses of Class 17.3. Subclass 17.3a, represented by sequences 17.3 9T and 17.3, contains all sequences with visible signals in the area indicated by the left grey rectangle. Subclass 17.b, represented by sequences 17.3s27 and 17.3 9T, contains all sequences with spectra containing signals in the area indicated by the right grey rectangle. Subclass 17.3c, represented by sequence 17.3 8C, contains all sequences with spectra that contain one signal in the area indicated by the grey rectangle and no signals in the areas indicated by the right and left grey rectangles. Subclass 17.3d, represented by sequences 17.3s27 and 17.3s26, contains all sequences with spectra with two signals in the middle grey area.

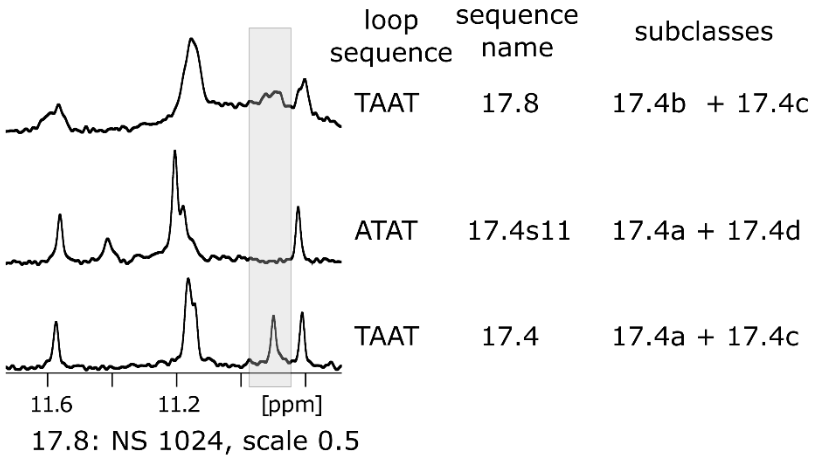

Figure S40: Subclasses of Class 17.4. Spectra of three sequences in Class 17.4 that illustrate the subclasses of Class 17.4. Each sequence belongs to either Class 17.4a or Class 17.4b and to either Class 17.4c or 17.4d. Subclass 17.4b, represented by sequence 17.8, contains sequences with significantly lower signal to noise ratios then Subclass 17.4a, represented by sequences 17.4s11 and 17.4. Subclass 17.4d, represented by sequence 17.4s11, contains all sequences with no signal in the area of the with spectrum around 11.9 ppm (indicated by a grey rectangle), while spectra of sequences in Subclass 17.4c,

represented by sequences 17.8 and 17.4, have one signal in this area. The spectrum of sequence 17.8 was measured with 1024 scans and is displayed with scale 0.25.

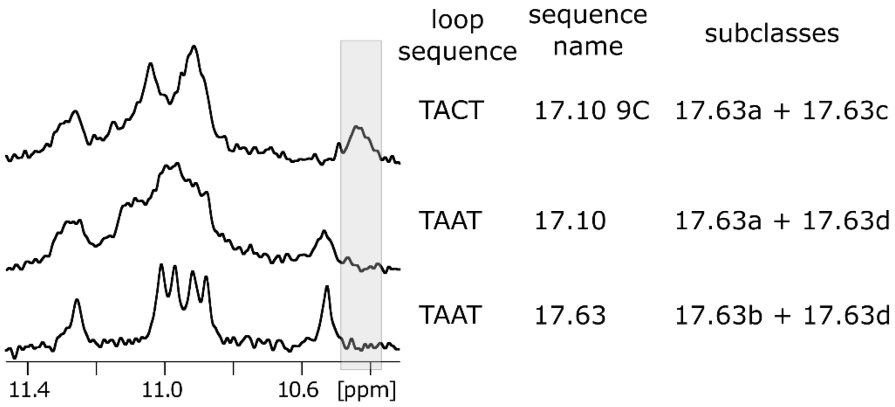

Figure S41: Subclasses in Class 17.63. Spectra of three sequences in Class 17.63 that illustrate subclasses in Class 17.63. Each sequence belongs to either Class 17.63a or Class 17.63b and to either 17.63c or 17.63d. Subclass 17.63a, represented by sequences 17.10 and 17.10 9C, contains sequences with significantly lower signal to noise ratios than Subclass 17.63b, represented by sequence 17.63. Subclass 17.63c, represented by sequence 17.10 9C, contains all sequences with a signal in the area of the spectrum around 10.4 ppm (indicated by a grey rectangle), while the spectra of sequences from Subclass 17.63d, represented by sequences 17.10 and 17.63, do not have a signal in this area. Spectra of sequences 17.10 and 17.10 9C were measured with 1024 scans and are displayed with scale 0.5.

Table S5: Comparison of results of computer clustering of the whole dataset and manual sorting. Each column corresponds to one cluster (with numbers from -1 up to 9). Each row corresponds to one class identified during manual sorting. "17.4+17.4s26" is a class of sequences with ^{1}H NMR spectra with characteristics of both Class 17.4 and 17.4s26. Class "no pattern" contains sequences with ^{1}H NMR spectra containing signals with no clear pattern in the G-quadruplex part of spectrum. Class "no G4 signals" contains all sequences with no signals in the G-quadruplex part of the ^{1}H NMR spectrum.

Class	-1	0	1	2	3	4	5	6	7	8	9
17.3	3	0	18	18	18	18	6	0	0	0	0
17.4a	7	0	0	0	0	0	0	7	17	0	0
17.4b	3	0	0	0	0	0	0	0	0	0	0
17.63a	1	0	0	0	0	0	0	0	0	85	0
17.63b	6	0	0	0	0	0	0	0	0	3	0
17.28	8	0	0	0	0	0	0	0	0	0	0
17.36	16	0	0	0	0	0	0	0	0	0	0
17.49	6	0	0	0	0	0	0	0	0	0	0
17.109	1	0	0	0	0	0	0	0	0	0	0
17.118	9	9	0	0	0	0	0	0	0	0	0
17.154	8	0	0	0	0	0	0	0	0	0	0
17.180	7	0	0	0	0	0	0	0	0	0	0
17.4s26	36	0	0	0	0	0	0	0	0	10	7
17.4 + 17.4s26	4	0	0	0	0	0	0	4	0	0	0
no clear pattern	7	0	0	0	0	0	0	0	0	0	0
no G4 signals	32	0	0	0	0	0	0	0	0	0	0

Table S6: Comparison of results of computer clustering of the tetrad library and manual sorting. Each column corresponds to one cluster (with numbers from -1 up to 9). Each row corresponds to one class identified during manual sorting. Class "no pattern" contains sequences with ^{1}H NMR spectra containing signals with no clear pattern in the G-quadruplex part of the spectrum. Class "no G4 signals" contains all sequences with no signals in G-quadruplex part of ^{1}H NMR spectrum.

Class	-1	0	1	2	3
17.3	1	0	0	0	0

17.4a	0	0	0	0	12
17.4b	0	0	0	0	3
17.63a	0	0	0	6	0
17.63b	2	0	6	1	0
17.28	1	0	0	7	0
17.36	13	0	0	3	0
17.49	6	0	0	0	0
17.109	1	0	0	0	0
17.118	9	9	0	0	0
17.154	7	1	0	0	0
17.180	6	1	0	0	0
no clear pattern	6	0	0	1	0
no G4 signals	32	0	0	0	0

Table S7: Comparison of results of computer clustering and manual sorting for sequences in the 17.4 loop library. Each column corresponds to one cluster (with numbers from -1 up to 9). Each row corresponds to one class identified during manual sorting and distribution of its sequences into individual clusters.

Class	-1	0	1	2	3
17.4a	7	0	5	8	7
17.4s26	30	23	0	0	30
17.4 + 17.4s26	3	1	0	4	3

Table S8: Interpretation of clusters created during clustering of the whole dataset with respect to classes identified during manual sorting.

Cluster	Sequences	Interpretation	Considered a subclass
-1	154	No clear interpretation	
0	9	part of 17.118	
1	18	part of 17.3 – HYTH – 18 out of 18	Intersection of 17.3b and 17.3d
2	18	part of 17.3 - HYCH – 18 out of 18	Part of 17.3d which is not in 17.3b
3	18	part of 17.3 - HYAH – 18 out of 18	17.3c
4	18	part of 17.3 – HAMH – 17 out of 18	Part of 17.3a which is not in 17.3b
5	6	part of 17.3 – HATH – 6 out of 9	Intersection of 17.3b and 17.3a
6	11	part of 17.4 + part of mix of 17.4 and 17.4s26	
7	17	Majority of 17.4 – HAAH 17 out of 23	17.4c
8	98	17.10 + part of 17.4s26	
9	7	part of 17.4s26	

Table S9: Interpretation of clusters created during clustering of the tetrad library with respect to classes identified during manual sorting.

Cluster	Sequences	Interpretation
-1	84	No clear interpretation
0	11	Part of class 17.118 + two other sequences
1	6	Majority of 17.63b
2	18	Part of sequences with middle signal to noise ratio
3	15	17.4

Table S10: Interpretation of clusters created during clustering of the 17.3 loop library.

Cluster	Sequences	Interpretation	Considered a subclass
-1	3	No clear interpretation	
0	18	HYTH 18 out of 18	Intersection of 17.3b and 17.3d
1	18	HYCH 18 out of 18	Part of 17.3d which is not in 17.3b
2	18	HYAH 18 out of 18	17.3c
3	17	HAMH 17 out of 18	Part of 17.3a which is not in 17.3b
4	7	HATH 7 out of 9	Intersection of 17.3b and 17.3a

Table S11: Interpretation of clusters created during clustering of the 17.4 loop library with respect to classes identified during manual sorting.

Cluster	Sequences	Interpretation	Considered a subclass
-1	40	17.4s26 + part of 17.4	
0	24	17.4s26	
1	5	part of 17.4 - HAAH 5 out of 9	17.4c
2	12	part of 17.4 + part of mix	

Table S12: Interpretation of clusters created during clustering of the 17.10 loop library with respect to classes identified during manual sorting

Cluster	Sequences	Interpretation	Considered a subclass
-1	31	No clear interpretation	
0	5	HAYW 5 out of 12	17.63c
1	9	AHHH 9 out of 27	
2	36	YYHH 33 out of 36 + 3xAYHH	

Table S13: List of all subclasses identified using computer clustering.

Major class	Name of subclass	Number of sequences	Loop sequence	Definition in NMR spectrum
17.3	17.3a	27	HAHH	Visible signal of G1 in region 11.450 ppm to 11.375 ppm
	17.3b	27	HHTH	Signal in region 10.95 ppm to 10.87 ppm
	17.3c	18	HYAH	Not visible G1 in region 11.450 ppm to 11.375 ppm, no signal in region 10.95 ppm to 10.87 ppm, and one signal in region 11.13 ppm to 11.06 ppm
	17.3d	36	HYYH	Two signals in region 11.13 ppm to 11.06 ppm
17.4	17.4c	23	HAAH	One signal in region 10.97 ppm to 10.85 ppm
	17.4d	11	Not HAAH	No signal in region 10.97 ppm to 10.85 ppm
17.63	17.63c	10	HAYW (10 out of 12)	One signal in region 10.49 ppm to 10.37 ppm
	17.63d	85	HHHH (85 out of 95)	No signal in region 10.49 ppm to 10.37 ppm

Table S14: Detailed results of computer clustering

Table S14: Table with results of computer clustering. The column "Name" contains the name of the sequence, the columns "Spectrum parameters" contains parameters of the spectrum chosen as representative, the column "NS" contains the number of scans, the column "Prep." contains "1" for spectra which were measured on samples from the first preparation and "2" for samples from the second preparation, the column "Time" contains "P" for spectra measured within 3 days of preparation and "W" for spectra measured after weeks on the bench, the column "c(µM)" indicates the DNA concentration of the sample used for the measurement, the column "Filtered out" contains "yes" for spectra which were filtered out due to a low signal during preprocessing of spectra for computer clustering (for details, see Methods), the section "Cluster" indicates the number of clusters to which given sequence belongs, the column "Whole dataset" contains the results of clustering of the whole dataset, the column "Tetrad" contains results of clustering of the tetrad library, the column "17.3 loop" contains results of clustering

of the 17.3 loop library, the column "17.4 loop" contains results of clustering of the 17.4 loop library, and the column "17.10 loop" contains results of clustering of the 17.10 loop library. If the cell is crossed out, it means that spectrum of given sequence was not part of a clustered dataset (either because it did not belong to the analyzed library or because it was filtered out).

Name	Spectrum parameters				Filtered out	Cluster				
	NS	Prep.	Time	*c* (μM)		Whole dataset	Tetrad	17.3 loop	17.4 loop	17.10 loop
17.3	256	1	P	100	no	4	-1	3		
17.4	256	1	P	100	no	7	3		-1	
17.5	256	1	P	100	no	7	3			
17.6	256	1	P	100	no	7	3			
17.7	1024	1	P	100	no	-1	3			
17.8	1024	1	P	100	no	-1	3			
17.9	1024	1	P	100	no	-1	3			
17.10	1024	1	P	100	no	8	2			-1
17.11	256	1	P	100	no	8	2			
17.12	256	1	P	100	no	8	2			
17.13	256	1	P	100	no	8	2			
17.14	256	1	P	100	no	8	2			
17.15	256	1	P	100	no	8	2			
17.18	256	1	P	100	no	-1	3			
17.19	256	1	P	100	no	7	3			
17.20	256	1	P	100	no	7	3			
17.21	256	1	P	100	no	-1	3			
17.22	256	1	P	100	no	7	3			
17.23	256	1	P	100	no	7	3			
17.24	256	1	P	100	no	-1	3			
17.25	256	1	P	100	no	7	3			
17.26	256	1	P	100	no	7	3			
17.27	1024	1	W	100	no	-1	2			
17.28	1024	1	W	100	no	-1	-1			
17.29	1024	1	W	100	no	-1	2			
17.30	1024	1	W	100	no	-1	2			
17.31	1024	1	W	100	no	-1	2			
17.32	1024	1	W	100	no	-1	2			
17.33	1024	1	W	100	no	-1	2			
17.34	1024	1	W	100	no	-1	2			
17.35	1024	1	W	100	no	-1	2			
17.36	1024	1	W	100	no	-1	-1			
17.37	1024	1	W	100	no	-1	-1			
17.38	1024	1	W	100	no	-1	2			
17.39	1024	1	W	100	no	-1	2			
17.40	1024	1	W	100	no	-1	-1			
17.41	512	2	W	50	no	-1	-1			
17.42	512	2	P	50	no	-1	-1			
17.43	1024	1	W	100	no	-1	-1			
17.44	1024	1	W	100	no	-1	-1			
17.45	1024	2	P	50	no	-1	-1			
17.46	1024	1	W	100	no	-1	-1			
17.47	1024	1	W	100	no	-1	-1			
17.48	512	2	W	50	no	-1	-1			

17.49	1024	1	P	100	no	-1	-1			
17.50	1024	1	P	100	no	-1	-1			
17.51	256	1	P	100	no	-1	-1			
17.52	1024	1	W	100	no	-1	-1			
17.53	1024	1	W	100	no	-1	2			
17.54	1024	1	W	100	no	-1	-1			
17.55	1024	1	W	100	no	-1	-1			
17.56	512	2	W	50	no	-1	-1			
17.57	1024	1	W	100	no	-1	-1			
17.58	1024	1	W	100	no	-1	-1			
17.59	256	1	P	100	no	-1	-1			
17.60	1024	2	W	70	no	-1	-1			
17.61	512	2	W	50	no	-1	-1			
17.62	512	2	W	40	no	-1	-1			
17.63	256	1	P	100	no	-1	2			
17.64	256	1	P	100	no	-1	-1			
17.65	256	1	P	100	no	-1	-1			
17.66	256	1	P	100	no	-1	1			
17.67	256	1	P	100	no	-1	1			
17.68	256	1	P	100	no	8	1			
17.69	256	1	P	100	no	-1	1			
17.70	256	1	P	100	no	8	1			
17.71	256	1	P	100	no	8	1			
17.72	1024	1	W	100	no	-1	0			
17.73	256	1	P	100	no	-1	-1			
17.74	256	1	P	100	yes					
17.75	256	1	P	100	no	-1	-1			
17.76	256	1	P	100	yes					
17.77	2056	1	W	100	no	-1	-1			
17.78	256	1	P	100	no	-1	-1			
17.79	256	1	P	100	yes					
17.80	256	1	P	100	yes					
17.81	256	1	P	100	no	-1	-1			
17.82	1024	1	W	100	no	-1	-1			
17.83	256	1	P	100	yes					
17.84	256	1	P	100	yes					
17.85	256	1	P	100	yes					
17.86	256	1	P	100	no	-1	-1			
17.87	256	1	P	100	yes					
17.88	256	1	P	100	yes					
17.89	256	1	P	100	yes					
17.90	1024	1	W	100	no	-1	-1			
17.91	1024	1	W	100	no	0	0			
17.92	1024	1	W	100	no	-1	-1			
17.93	1024	1	W	100	no	-1	-1			
17.94	1024	1	W	100	no	0	0			
17.95	1024	1	W	100	no	-1	-1			
17.96	1024	1	W	100	no	-1	-1			
17.97	1024	1	W	100	no	-1	-1			
17.98	1024	1	W	100	no	-1	-1			
17.99	256	1	W	100	no	-1	-1			

17.100	1024	1	W	100	no	0	0			
17.101	256	1	P	100	yes					
17.102	256	1	P	100	yes					
17.103	256	1	P	100	yes					
17.104	256	1	P	100	yes					
17.105	256	1	P	100	yes					
17.106	256	1	P	100	no	-1	-1			
17.107	256	1	P	100	yes					
17.108	256	1	P	100	no	-1	-1			
17.109	1024	1	P	100	no	-1	-1			
17.110	256	1	P	100	no	-1	-1			
17.111	256	1	P	100	no	-1	-1			
17.112	256	1	P	100	no	-1	-1			
17.113	256	1	P	100	no	-1	-1			
17.114	256	1	P	100	yes					
17.115	256	1	P	100	yes					
17.116	256	1	P	100	yes					
17.117	256	1	P	100	no	-1	-1			
17.118	256	1	P	100	no	0	0			
17.119	1024	1	P	100	yes					
17.120	256	1	P	100	yes					
17.121	256	1	P	100	no	-1	-1			
17.122	256	1	P	100	yes					
17.123	256	1	P	100	yes					
17.124	256	1	P	100	yes					
17.125	256	1	P	100	yes					
17.126	256	1	P	100	yes					
17.127	256	1	P	100	yes					
17.128	256	1	P	100	yes					
17.129	256	1	P	100	yes					
17.130	256	1	P	100	no	-1	-1			
17.131	256	1	P	100	no	-1	-1			
17.132	256	1	P	100	yes					
17.133	256	1	P	100	no	-1	-1			
17.134	256	1	P	100	yes					
17.135	256	1	P	100	yes					
17.136	256	1	P	100	yes					
17.137	256	1	P	100	yes					
17.138	256	1	P	100	yes					
17.139	256	1	P	100	yes					
17.140	256	1	P	100	yes					
17.141	256	1	P	100	yes					
17.142	256	1	P	100	yes					
17.143	256	1	P	100	yes					
17.144	256	1	P	100	yes					
17.145	256	1	P	100	yes					
17.146	256	1	P	100	no	-1	-1			
17.147	256	1	P	100	yes					
17.148	256	1	P	100	yes					
17.149	256	1	P	100	yes					
17.150	256	1	P	100	yes					
17.151	256	1	P	100	yes					
17.152	256	1	P	100	yes					
17.153	1024	1	P	100	no	-1	-1			
17.154	256	1	W	100	no	-1	-1			

17.155	256	1	W	100	no	-1	0			
17.156	256	1	P	100	no	-1	-1			
17.157	256	1	P	100	yes					
17.158	256	1	P	100	yes					
17.159	256	1	P	100	yes					
17.160	256	1	P	100	yes					
17.161	256	1	P	100	yes					
17.162	1024	1	W	100	no	-1	-1			
17.163	256	1	P	100	yes					
17.164	256	1	W	100	no	-1	-1			
17.165	256	1	P	100	yes					
17.166	256	1	P	100	no	-1	-1			
17.167	256	1	P	100	yes					
17.168	256	1	P	100	yes					
17.169	256	1	P	100	no	-1	-1			
17.170	256	1	P	100	yes					
17.171	1024	1	W	100	no	-1	-1			
17.172	256	1	P	100	no	-1	-1			
17.173	256	1	W	100	no	-1	-1			
17.174	256	1	P	100	yes					
17.175	256	1	P	100	yes					
17.176	1024	2	W	60	no	-1	-1			
17.177	256	1	P	100	yes					
17.178	256	1	P	100	yes					
17.179	256	1	P	100	yes					
17.180	1024	1	P	100	no	-1	-1			
17.181	1024	1	P	100	no	-1	-1			
17.182	1024	1	P	100	no	-1	-1			
17.183	256	1	P	100	yes					
17.184	256	1	P	100	yes					
17.185	256	1	P	100	yes					
17.186	256	1	P	100	yes					
17.187	256	1	P	100	yes					
17.188	256	1	P	100	yes					
17.189	256	1	P	100	yes					
17.190	1024	1	P	100	no	0	0			
17.191	256	1	P	100	no	-1	-1			
17.192	256	1	P	100	yes					
17.193	256	1	P	100	yes					
17.194	256	1	P	100	yes					
17.195	256	1	P	100	yes					
17.196	256	1	P	100	yes					
17.197	256	1	P	100	yes					
17.198	256	1	P	100	yes					
17.199	1024	1	P	100	no	0	0			
17.200	256	1	P	100	yes					
17.201	256	1	P	100	yes					
17.202	256	1	P	100	yes					
17.203	256	1	P	100	yes					
17.204	256	1	P	100	yes					
17.205	256	1	P	100	yes					
17.206	256	1	P	100	yes					
17.207	1024	1	P	100	no	-1	-1			
17.208	1024	1	W	100	no	-1	-1			
17.209	256	1	P	100	yes					

17.210	256	1	W	100	no	-1	-1			
17.211	256	1	P	100	yes					
17.212	256	1	P	100	no	-1	-1			
17.213	256	1	P	100	yes					
17.214	256	1	P	100	no	-1	-1			
17.215	256	1	P	100	no	-1	-1			
17.216	256	1	P	100	no	-1	-1			
17.217	1024	1	W	100	no	-1	-1			
17.218	256	1	P	100	yes					
17.219	256	1	P	100	yes					
17.220	256	1	P	100	yes					
17.221	256	1	P	100	yes					
17.222	256	1	P	100	yes					
17.223	256	1	P	100	yes					
17.224	256	1	P	100	yes					
17.225	256	1	P	100	yes					
17.226	1024	1	W	100	no	0	0			
17.227	256	1	P	100	yes					
17.228	256	1	P	100	yes					
17.229	256	1	P	100	yes					
17.230	256	1	P	100	yes					
17.231	256	1	P	100	no	-1	-1			
17.232	256	1	P	100	yes					
17.233	256	1	P	100	yes					
17.234	1024	1	P	100	no	-1	-1			
17.235	1024	1	W	100	no	0	0			
17.236	256	1	P	100	yes					
17.237	256	1	P	100	no	-1	-1			
17.238	256	1	P	100	yes					
17.239	256	1	P	100	yes					
17.240	256	1	P	100	yes					
17.241	256	1	P	100	yes					
17.242	256	1	P	100	yes					
17.243	256	1	P	100	yes					
17.244	256	1	P	100	no	-1	-1			
17.245	256	1	P	100	yes					
17.246	256	1	P	100	yes					
17.247	256	1	P	100	yes					
17.248	256	1	P	100	yes					
17.249	256	1	P	100	yes					
17.250	256	1	P	100	no	-1	-1			
17.251	256	1	P	100	yes					
17.252	256	1	P	100	yes					
17.253	1024	1	W	100	no	0	0			
17.254	256	1	P	100	yes					
17.255	256	1	P	100	yes					
17.256	256	1	P	100	yes					
17.257	256	1	P	100	yes					
17.258	256	1	P	100	yes					
17.259	256	1	P	100	yes					
17.260	256	1	P	100	yes					
17.3 4A	256	1	P	100	no	4		3		
17.3 4C	256	1	P	100	no	4		3		
17.3 8C	256	1	P	100	no	3		2		
17.3 8T	256	1	P	100	no	3		2		

17.3 9C	256	1	P	100	no	4		3		
17.3 9T	256	1	P	100	no	4		4		
17.3 13A	256	1	P	100	no	4		3		
17.3 13C	256	1	P	100	no	4		3		
17.3s10	256	1	P	100	no	3		2		
17.3s11	256	1	P	100	no	3		2		
17.3s12	256	1	P	100	no	3		2		
17.3s13	256	1	P	100	no	3		2		
17.3s14	256	1	P	100	no	4		3		
17.3s15	256	1	P	100	no	4		3		
17.3s16	256	1	P	100	no	5		4		
17.3s17	256	1	P	100	no	-1		-1		
17.3s18	256	1	P	100	no	4		3		
17.3s19	256	1	P	100	no	5		4		
17.3s20	256	1	P	100	no	-1		-1		
17.3s21	256	1	P	100	no	4		3		
17.3s22	256	1	P	100	no	4		3		
17.3s23	256	1	P	100	no	5		4		
17.3s24	256	1	P	100	no	4		3		
17.3s25	256	1	P	100	no	4		3		
17.3s26	256	1	P	100	no	2		1		
17.3s27	256	1	P	100	no	1		0		
17.3s28	256	1	P	100	no	3		2		
17.3s29	256	1	P	100	no	3		2		
17.3s30	256	1	P	100	no	2		1		
17.3s31	256	1	P	100	no	1		0		
17.3s32	256	1	P	100	no	3		2		
17.3s33	256	1	P	100	no	3		2		
17.3s34	256	1	P	100	no	2		1		
17.3s35	256	1	P	100	no	1		0		
17.3s36	256	1	P	100	no	3		2		
17.3s37	256	1	P	100	no	3		2		
17.3s38	256	1	P	100	no	2		1		
17.3s39	256	1	P	100	no	1		0		
17.3s40	256	1	P	100	no	3		2		
17.3s41	256	1	P	100	no	3		2		
17.3s42	256	1	P	100	no	2		1		
17.3s43	256	1	P	100	no	1		0		
17.3s44	256	1	P	100	no	3		2		
17.3s45	256	1	P	100	no	3		2		
17.3s46	256	1	P	100	no	2		1		
17.3s47	256	1	P	100	no	1		0		
17.3s48	256	1	P	100	no	3		2		
17.3s49	256	1	P	100	no	3		2		
17.3s50	256	1	P	100	no	4		3		
17.3s51	256	1	P	100	no	4		3		
17.3s52	256	1	P	100	no	-1		-1		
17.3s53	256	1	P	100	no	5		4		
17.3s54	256	1	P	100	no	4		3		
17.3s55	256	1	P	100	no	4		3		

17.3s56	256	1	P	100	no	5		4		
17.3s57	256	1	P	100	no	5		4		
17.3s58	256	1	P	100	no	2		1		
17.3s59	256	1	P	100	no	2		1		
17.3s60	256	1	P	100	no	1		0		
17.3s61	256	1	P	100	no	1		0		
17.3s62	256	1	P	100	no	2		1		
17.3s63	256	1	P	100	no	2		1		
17.3s64	256	1	P	100	no	1		0		
17.3s65	256	1	P	100	no	1		0		
17.3s66	256	1	P	100	no	2		1		
17.3s67	256	1	P	100	no	2		1		
17.3s68	256	1	P	100	no	1		0		
17.3s69	256	1	P	100	no	1		0		
17.3s70	256	1	P	100	no	2		1		
17.3s71	256	1	P	100	no	2		1		
17.3s72	256	1	P	100	no	1		0		
17.3s73	256	1	P	100	no	1		0		
17.3s74	256	1	P	100	no	2		1		
17.3s75	256	1	P	100	no	2		1		
17.3s76	256	1	P	100	no	1		0		
17.3s77	256	1	P	100	no	1		0		
17.3s78	256	1	P	100	no	2		1		
17.3s79	256	1	P	100	no	2		1		
17.3s80	256	1	P	100	no	1		0		
17.3s81	256	1	P	100	no	1		0		
17.4 4A	256	1	P	100	no	7			1	
17.4 4C	256	1	P	100	no	7			1	
17.4 8C	256	1	P	100	no	-1			0	
17.4 8T	256	1	P	100	no	9			0	
17.4 9C	256	1	P	100	no	8			0	
17.4 9T	256	1	P	100	no	-1			0	
17.4 13A	256	1	P	100	no	7			1	
17.4 13C	256	1	P	100	no	7			1	
17.4s10	256	1	P	100	no	6			2	
17.4s11	256	1	P	100	no	6			2	
17.4s12	256	1	P	100	no	-1			-1	
17.4s13	256	1	P	100	no	-1			-1	
17.4s14	256	1	P	100	no	-1			0	
17.4s15	256	1	P	100	no	8			0	
17.4s16	256	1	P	100	no	9			0	
17.4s17	256	1	P	100	no	-1			-1	
17.4s18	256	1	P	100	no	6			2	
17.4s19	256	1	P	100	no	-1			-1	
17.4s20	256	1	P	100	no	7			1	
17.4s21	256	1	P	100	no	7			-1	
17.4s22	256	1	P	100	no	-1			-1	
17.4s23	256	1	P	100	no	6			2	
17.4s24	256	1	P	100	no	7			2	
17.4s25	256	1	P	100	no	7			-1	

17.4s26	256	1	P	100	no	8			0	
17.4s27	256	1	P	100	no	8			0	
17.4s28	256	1	P	100	no	9			0	
17.4s29	256	1	P	100	no	9			0	
17.4s30	256	1	P	100	no	8			0	
17.4s31	256	1	P	100	no	-1			-1	
17.4s32	256	1	P	100	no	9			0	
17.4s33	256	1	P	100	no	9			0	
17.4s34	256	1	P	100	no	-1			-1	
17.4s35	256	1	P	100	no	-1			-1	
17.4s36	256	1	P	100	no	6			2	
17.4s37	256	1	P	100	no	6			2	
17.4s38	256	1	P	100	no	-1			-1	
17.4s39	256	1	P	100	no	-1			-1	
17.4s40	256	1	P	100	no	-1			-1	
17.4s41	256	1	P	100	no	6			2	
17.4s42	256	1	P	100	no	-1			-1	
17.4s43	256	1	P	100	no	-1			-1	
17.4s44	256	1	P	100	no	9			0	
17.4s45	256	1	P	100	no	-1			-1	
17.4s46	256	1	P	100	no	-1			-1	
17.4s47	256	1	P	100	no	-1			-1	
17.4s48	256	1	P	100	no	-1			0	
17.4s49	256	1	P	100	no	-1			0	
17.4s50	256	1	P	100	no	6			2	
17.4s51	256	1	P	100	no	6			2	
17.4s52	256	1	P	100	no	-1			-1	
17.4s53	256	1	P	100	no	-1			-1	
17.4s54	256	1	P	100	no	-1			-1	
17.4s55	256	1	P	100	no	-1			-1	
17.4s56	256	1	P	100	no	6			2	
17.4s57	256	1	P	100	no	6			2	
17.4s58	256	1	P	100	no	8			0	
17.4s59	256	1	P	100	no	-1			0	
17.4s60	256	1	P	100	no	8			0	
17.4s61	256	1	P	100	no	8			0	
17.4s62	256	1	P	100	no	8			0	
17.4s63	256	1	P	100	no	8			0	
17.4s64	256	1	P	100	no	-1			-1	
17.4s65	256	1	P	100	no	-1			-1	
17.4s66	256	1	P	100	no	-1			-1	
17.4s67	256	1	P	100	no	-1			-1	
17.4s68	256	1	P	100	no	-1			-1	
17.4s69	256	1	P	100	no	-1			-1	
17.4s70	256	1	P	100	no	-1			-1	
17.4s71	256	1	P	100	no	-1			-1	
17.4s72	256	1	P	100	no	-1			-1	
17.4s73	256	1	P	100	no	-1			0	
17.4s74	256	1	P	100	no	-1			-1	
17.4s75	256	1	P	100	no	-1			-1	

17.4s76	256	1	P	100	no	-1			-1	
17.4s77	256	1	P	100	no	-1			-1	
17.4s78	256	1	P	100	no	-1			-1	
17.4s79	256	1	P	100	no	-1			-1	
17.4s80	256	1	P	100	no	-1			-1	
17.4s81	256	1	P	100	no	-1			-1	
17.10 4A	1024	1	P	100	no	8				1
17.10 4C	256	1	P	100	no	8				-1
17.10 8C	256	1	P	100	no	8				2
17.10 8T	256	1	P	100	no	8				2
17.10 9C	1024	1	P	100	no	8				0
17.10 9T	1024	1	P	100	no	8				-1
17.10 13A	1024	1	P	100	no	8				-1
17.10 13C	256	1	P	100	no	-1				-1
17.10s10	256	1	P	100	no	8				1
17.10s11	256	1	P	100	no	8				1
17.10s12	256	1	P	100	no	8				2
17.10s13	256	1	P	100	no	8				2
17.10s14	1024	1	P	100	no	8				0
17.10s15	256	1	P	100	no	8				-1
17.10s16	1024	1	P	100	no	8				0
17.10s17	256	1	P	100	no	8				-1
17.10s18	256	1	P	100	no	8				-1
17.10s19	256	1	P	100	no	8				1
17.10s20	256	1	P	100	no	8				-1
17.10s21	256	1	P	100	no	8				-1
17.10s22	1024	1	P	100	no	8				0
17.10s23	1024	1	P	100	no	8				-1
17.10s24	1024	1	P	100	no	8				-1
17.10s25	256	1	P	100	no	8				-1
17.10s26	1024	1	P	100	no	8				2
17.10s27	1024	1	P	100	no	8				2
17.10s28	1024	1	P	100	no	8				2
17.10s29	1024	1	P	100	no	8				2
17.10s30	1024	1	P	100	no	8				2
17.10s31	1024	1	P	100	no	8				2
17.10s32	1024	1	P	100	no	8				-1
17.10s33	256	1	P	100	no	8				2
17.10s34	256	1	P	100	no	8				1
17.10s35	256	1	P	100	no	8				1
17.10s36	256	1	P	100	no	8				2
17.10s37	256	1	P	100	no	8				-1
17.10s38	256	1	P	100	no	8				-1
17.10s39	256	1	P	100	no	8				-1
17.10s40	256	1	P	100	no	8				2
17.10s41	256	1	P	100	no	8				1
17.10s42	256	1	P	100	no	8				-1
17.10s43	256	1	P	100	no	8				2
17.10s44	256	1	P	100	no	8				2
17.10s45	256	1	P	100	no	8				2

17.10s46	256	1	P	100	no	8				2
17.10s47	256	1	P	100	no	8				2
17.10s48	256	1	P	100	no	8				2
17.10s49	256	1	P	100	no	8				2
17.10s50	1024	1	P	100	no	8				0
17.10s51	256	1	P	100	no	8				-1
17.10s52	256	1	P	100	no	8				-1
17.10s53	256	1	P	100	no	8				-1
17.10s54	1024	1	P	100	no	8				-1
17.10s55	256	1	P	100	no	8				-1
17.10s56	1024	1	P	100	no	8				-1
17.10s57	256	1	P	100	no	8				-1
17.10s58	256	1	P	100	no	8				2
17.10s59	256	1	P	100	no	8				2
17.10s60	256	1	P	100	no	8				2
17.10s61	256	1	P	100	no	8				2
17.10s62	256	1	P	100	no	8				2
17.10s63	256	1	P	100	no	8				2
17.10s64	256	1	P	100	no	8				2
17.10s65	256	1	P	100	no	8				-1
17.10s66	256	1	P	100	no	8				2
17.10s67	256	1	P	100	no	8				2
17.10s68	256	1	P	100	no	8				2
17.10s69	256	1	P	100	no	8				2
17.10s70	256	1	P	100	no	8				2
17.10s71	256	1	P	100	no	8				2
17.10s72	256	1	P	100	no	8				2
17.10s73	256	1	P	100	no	8				2
17.10s74	256	1	P	100	no	8				-1
17.10s75	256	1	P	100	no	8				-1
17.10s76	256	1	P	100	no	8				2
17.10s77	256	1	P	100	no	8				-1
17.10s78	256	1	P	100	no	8				1
17.10s79	256	1	P	100	no	8				-1
17.10s80	256	1	P	100	no	8				1
17.10s81	256	1	P	100	no	8				-1

Section SI_structure

Structure of sequence 17.3 9C

In a previous study, we determined the structure of a reference monomeric G-quadruplex called 17.3 [19]. In this study, we analyzed NMR spectra of sequence 17.3 9C, which differs from sequence 17.3 by a point A to C mutation at position 9. ^{1}H NMR spectra of 17.3 and 17.3 9C are almost identical (Figure S42), and it was therefore possible to deduce assignments of the imino protons for sequence 17.3 9C. This similarity was confirmed by comparison of the anomeric-aromatic region of their NOESY spectra, in which sequential connectivity can be traced (Figure 3). The only significantly shifted signals are G10H8 and A8H6, both of which are next to the mutated site. Further analysis of both NOESY and TOCSY spectra showed only small differences, with the only exception being regions that involve imino hydrogens. One example is the aromatic-imino region in Figure S43, in which signal intensities are significantly lower for 17.3 9C than for 17.3.

We calculated the structure of 17.3 9C using the same protocol as previously used to calculate that of 17.3. This showed that these two structures contain essentially the same topology of G tetrads. However, some differences in the loops were observed. Unlike in the case of 17.3, in which T4 and T13 signals overlapped, in 17.3 9C we observed cross-peaks corresponding to contact between T13 and G10 and G11, whereas T4 did not show any such contacts.

Taken together, our results show that, in the case of three different G-quadruplexes (17.3, 17.3 9C, and PEA1-20 in [36]) similar ^{1}H NMR spectra reflect similar three-dimensional structures.

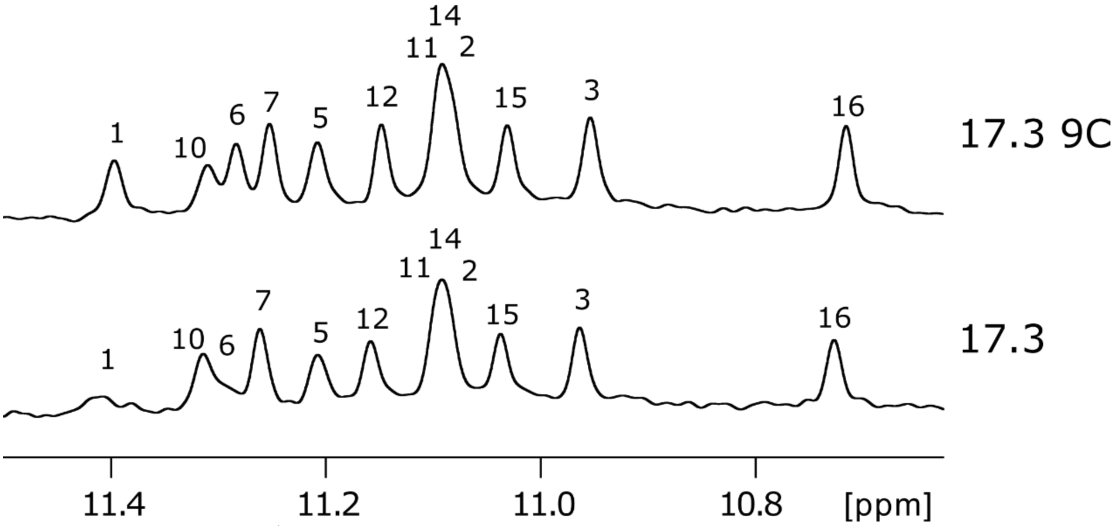

Figure S42: Comparison of ^{1}H NMR spectrum of sequences 17.3 and 17.3 9C with marked assignments.

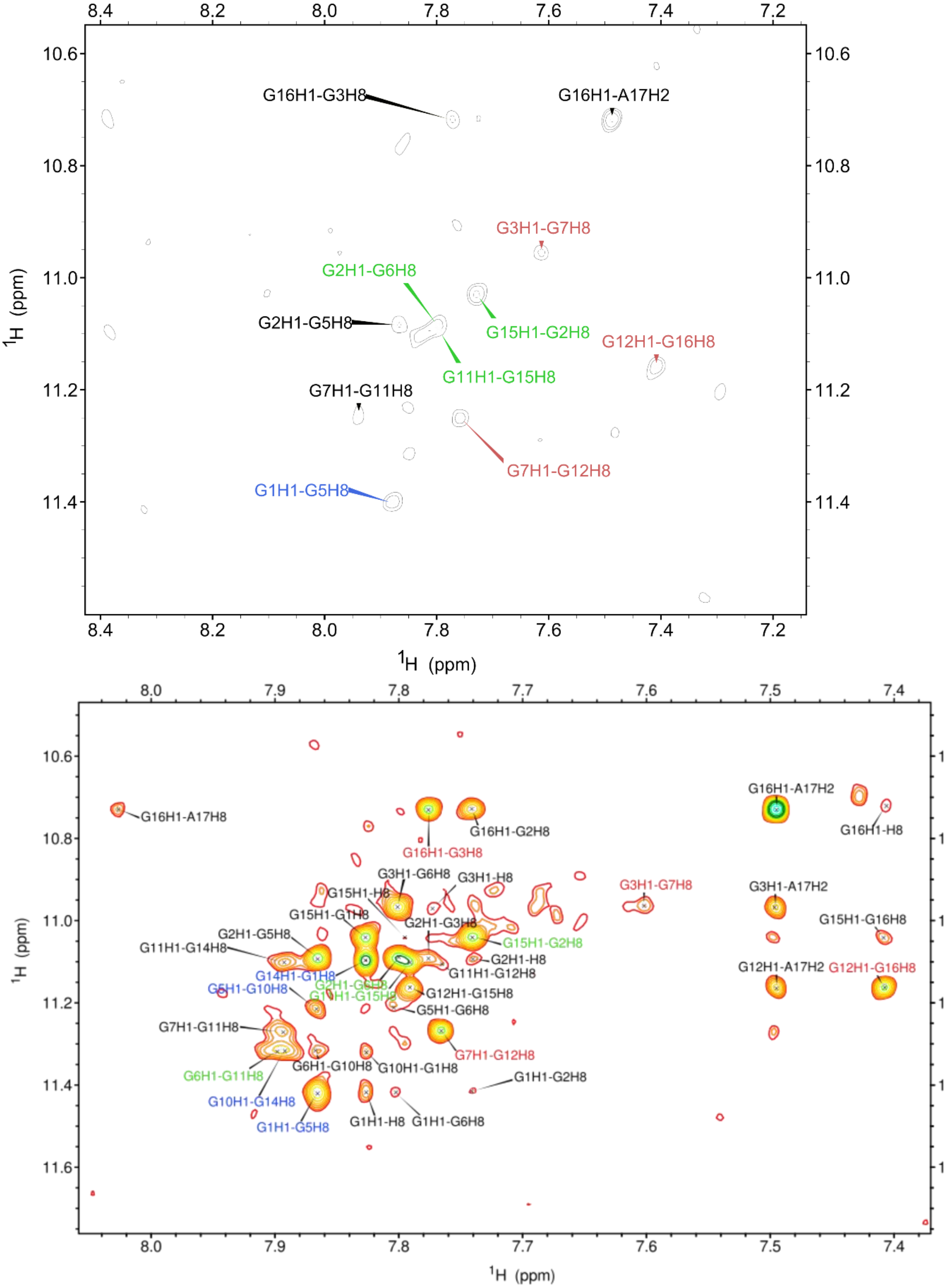

Figure S43: Comparison of the aromatic-imino region of the NOESY spectrum of sequence 17.3 9C (above) and 17.3 (bellow) with a mixing time of 400 ms at 298 K. The DNA was at a concentration of 1.7 mM in a buffer containing 20 mM Tris, pH 7.5, 200 mM KCl and 1 mM $MgCl_2$. H8–H1 cross-peaks within the G1-G5-G10-G14, G2-G6-G11-G15, and G3-G7-G12-G16 tetrads are labeled in blue, green and red, respectively. Cross-peaks corresponding to residues that are not part of the same tetrad are labeled in black.

Section SI_spectra

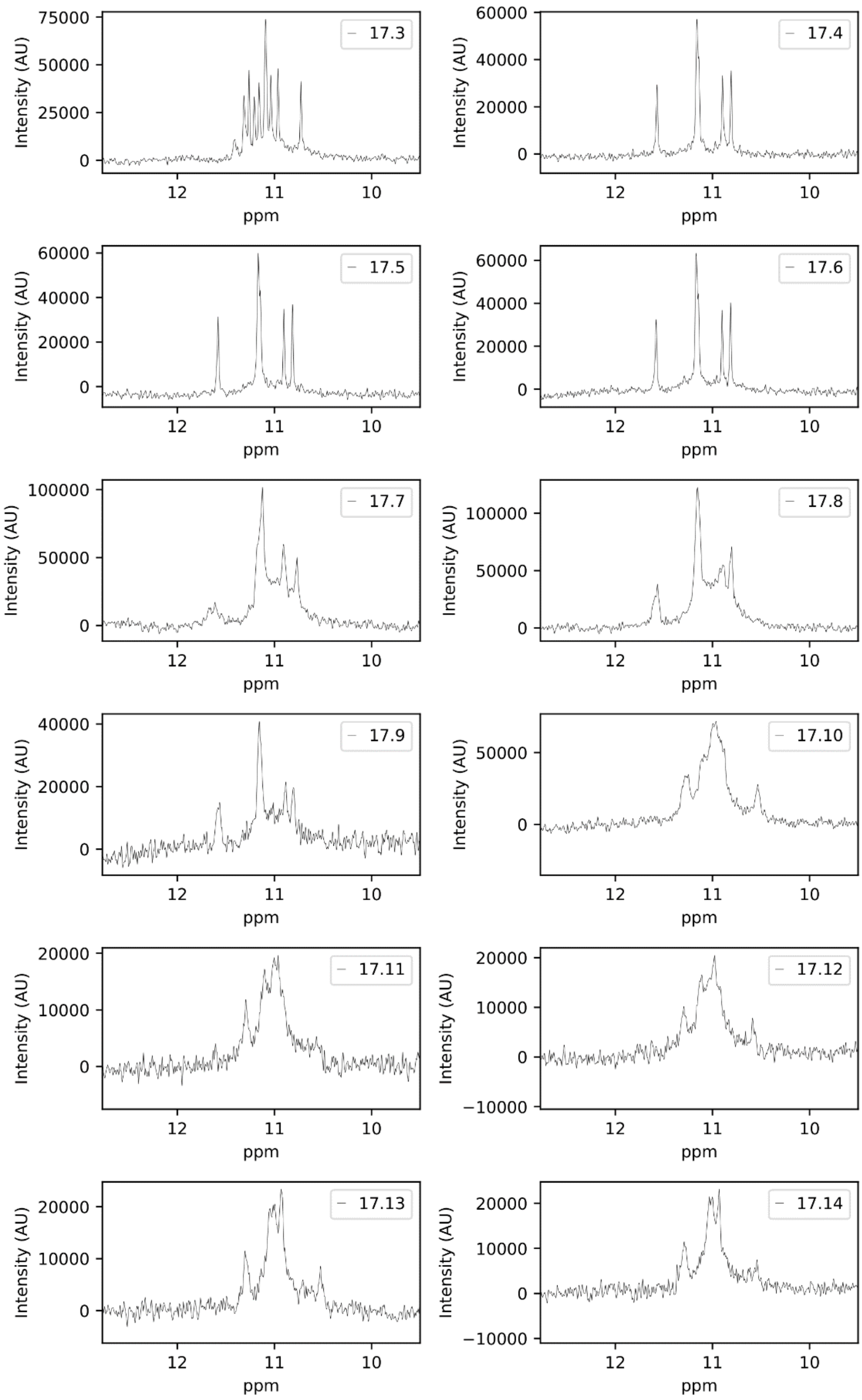
17.3
17.4
17.5
17.6
17.7
17.8
17.9
17.10
17.11
17.12
17.13
17.14
Intensity (AU)
ppm

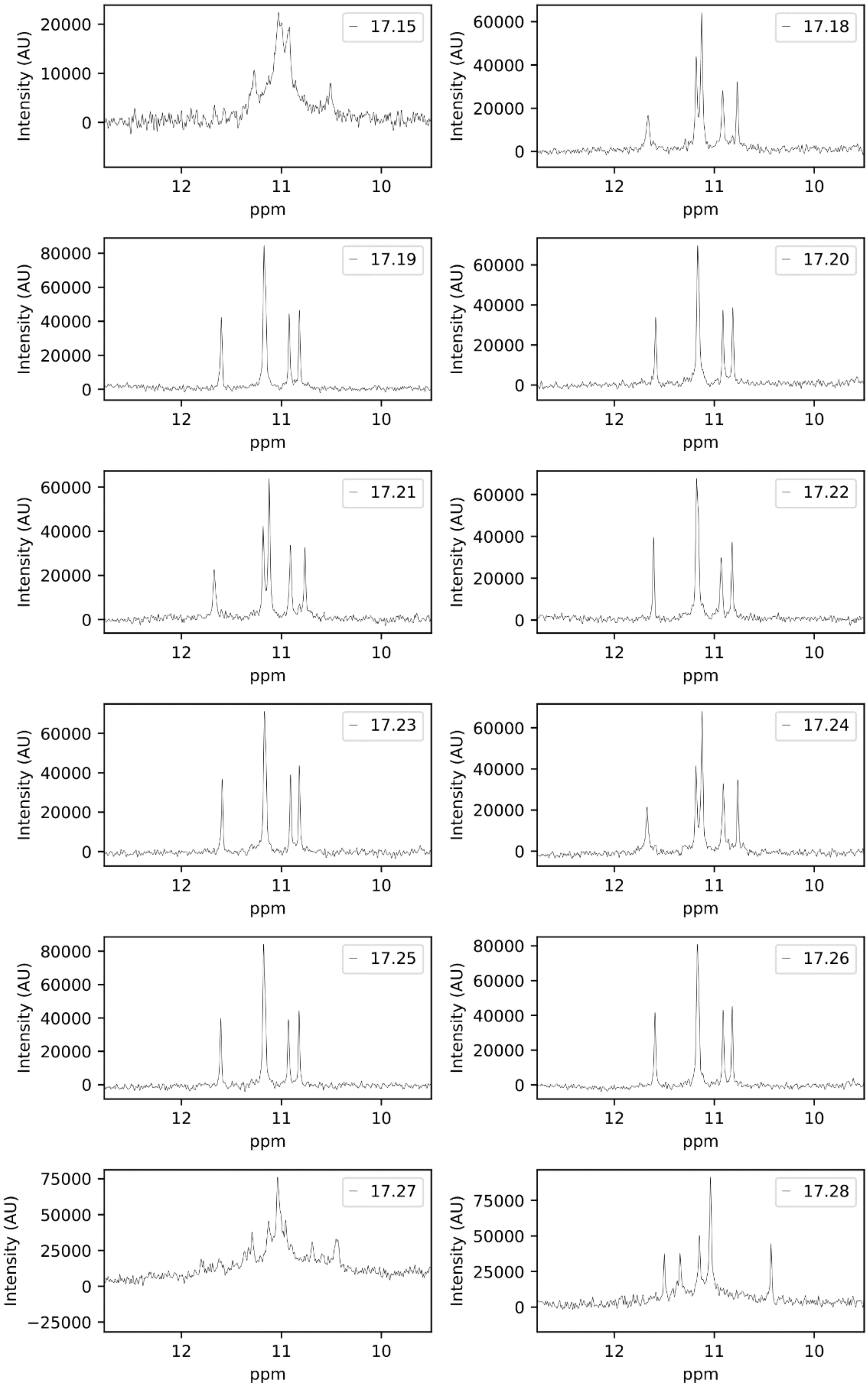
Intensity (AU)
ppm
17.15
17.18
17.19
17.20
17.21
17.22
17.23
17.24
17.25
17.26
17.27
17.28

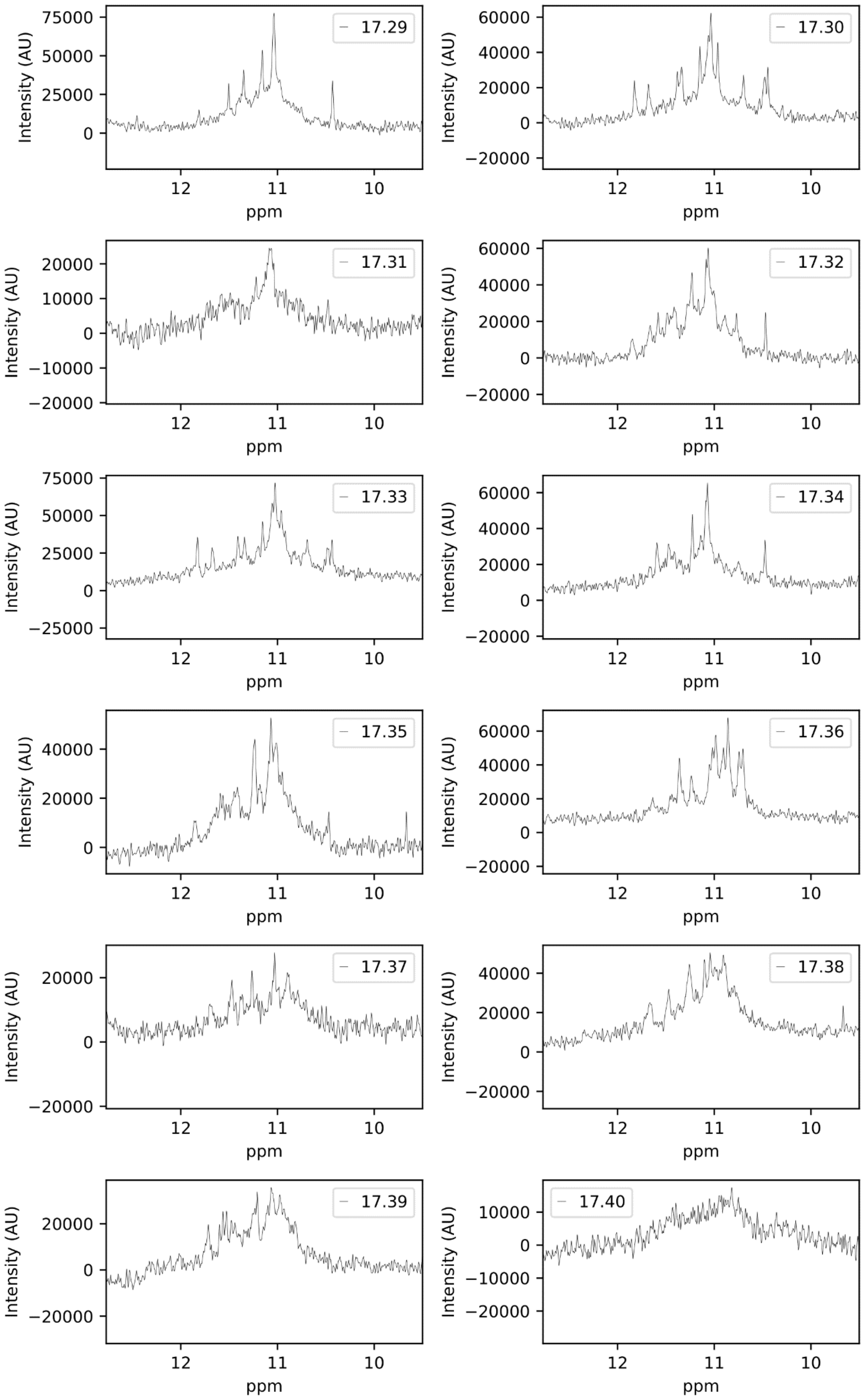
17.29
17.30
17.31
17.32
17.33
17.34
17.35
17.36
17.37
17.38
17.39
17.40
Intensity (AU)
ppm

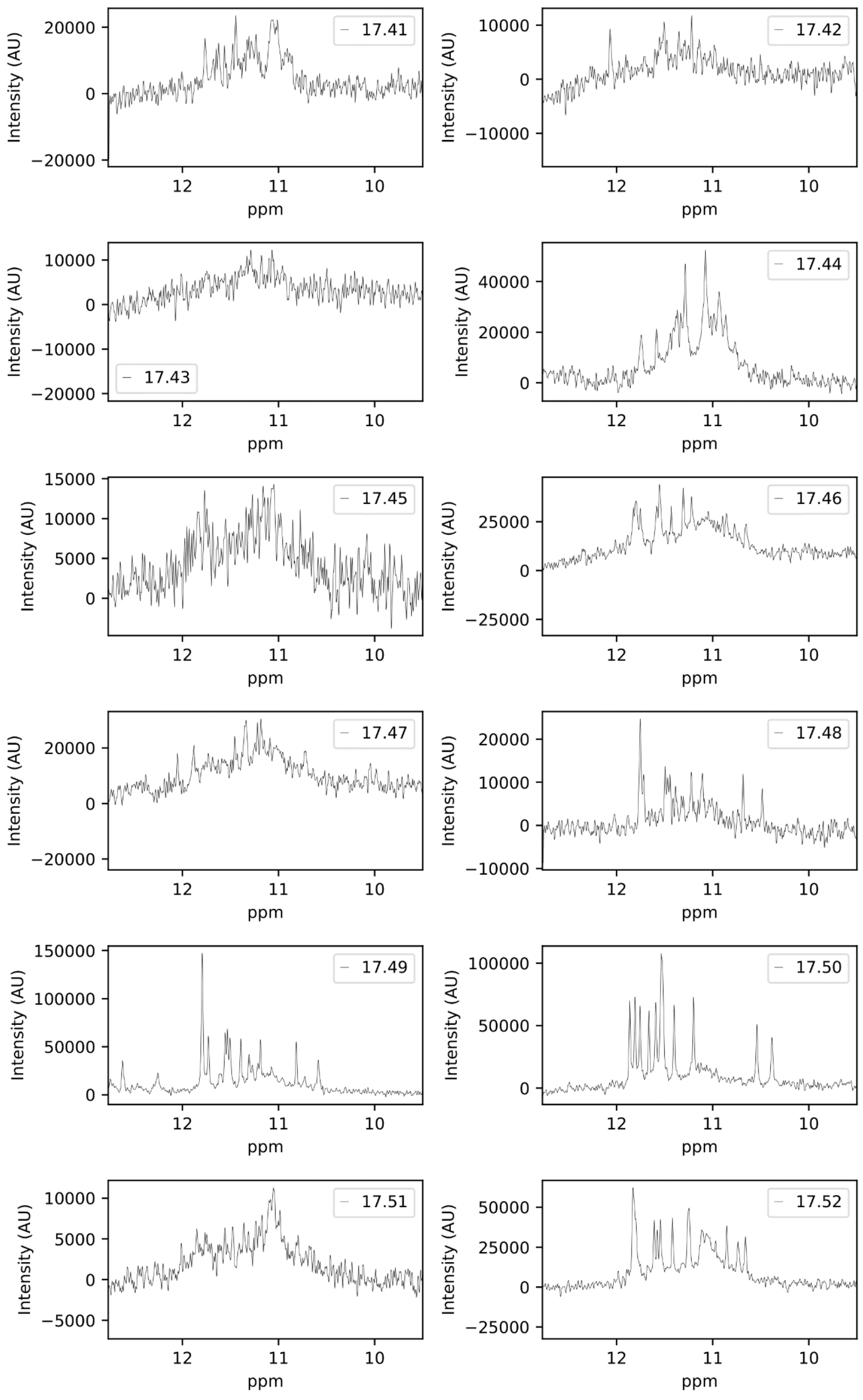
17.41
17.42
17.43
17.44
17.45
17.46
17.47
17.48
17.49
17.50
17.51
17.52
Intensity (AU)
ppm
12
11
10

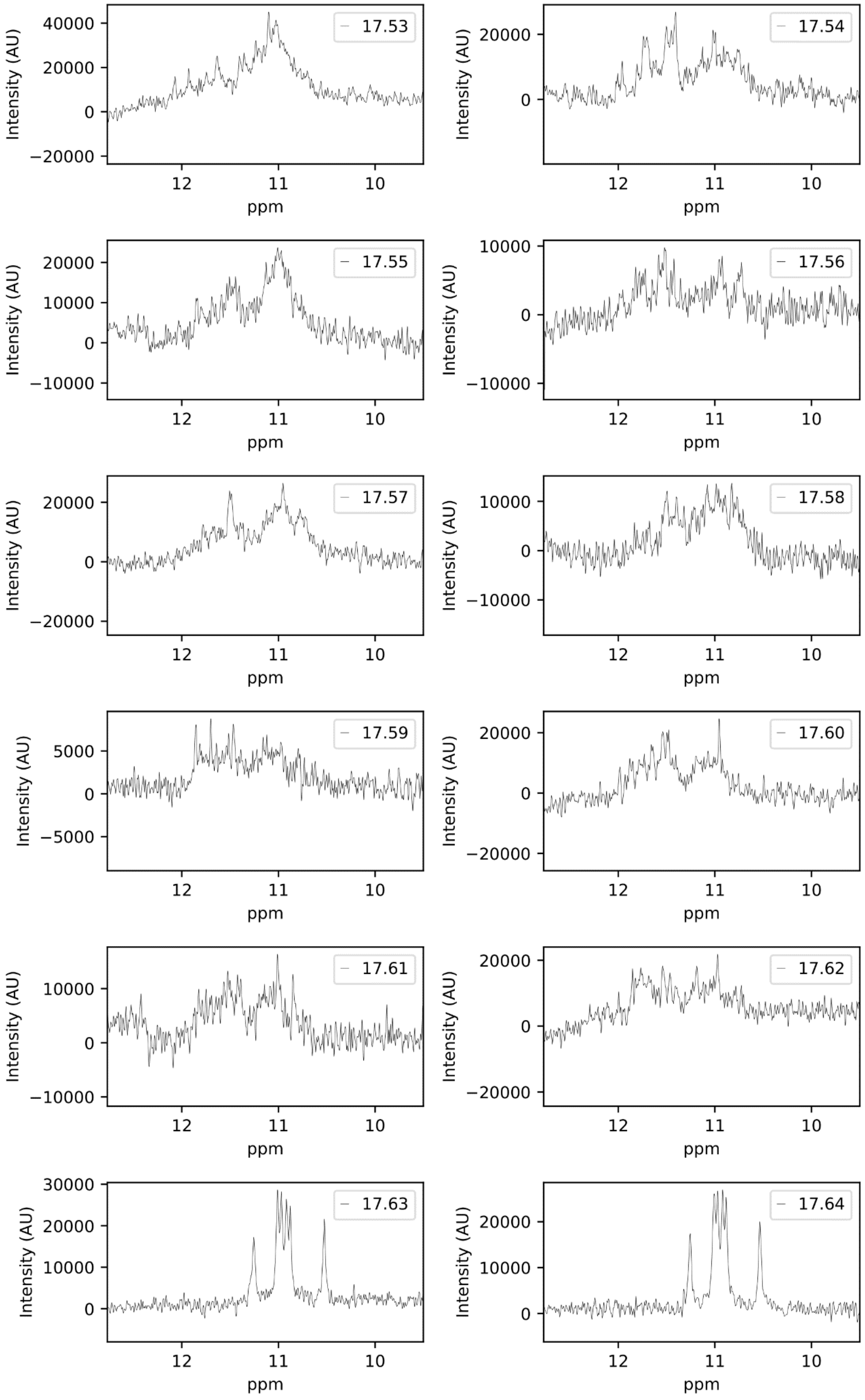
17.53
17.54
17.55
17.56
17.57
17.58
17.59
17.60
17.61
17.62
17.63
17.64
Intensity (AU)
ppm
12
11
10

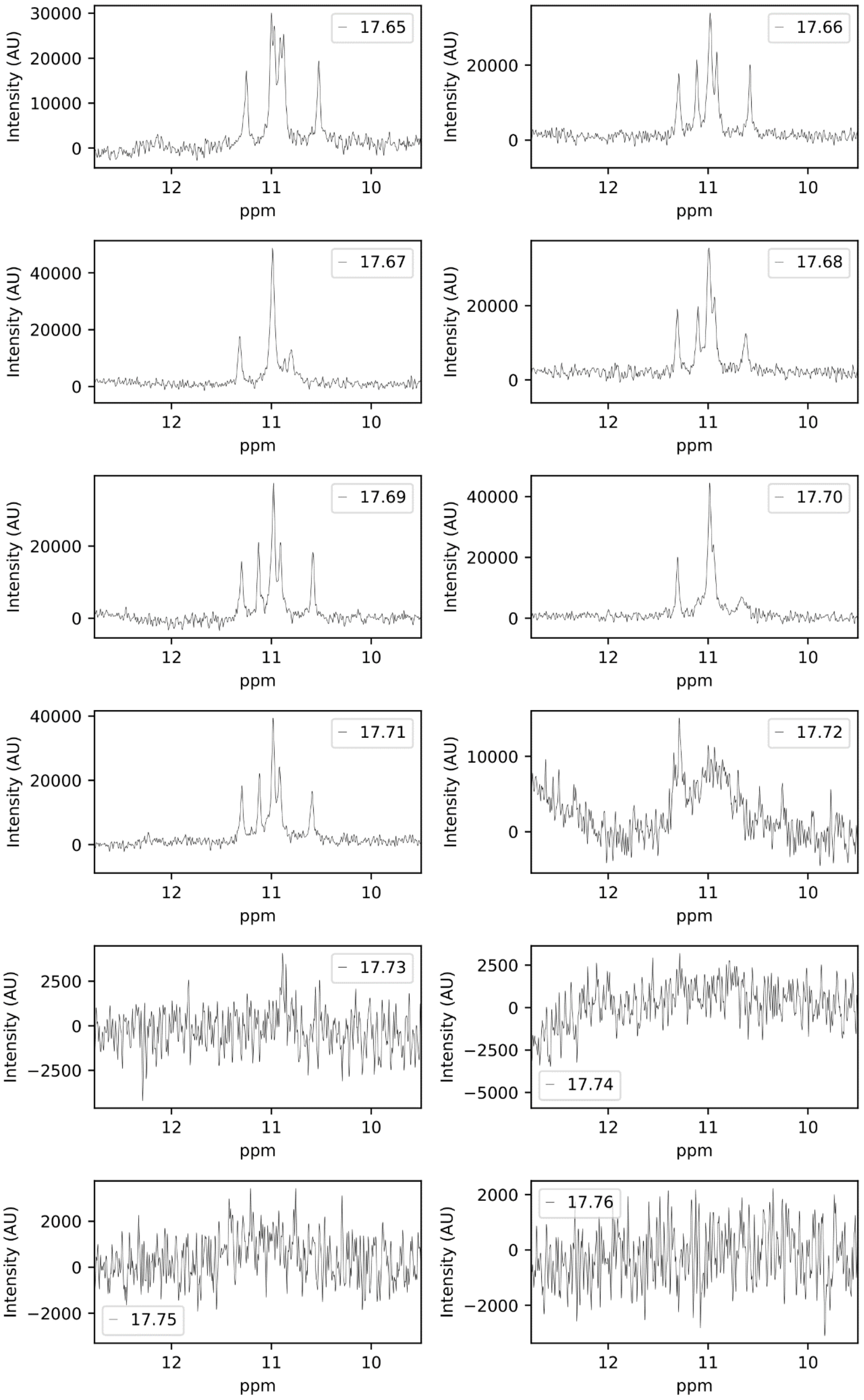
Intensity (AU)
ppm
17.65
17.66
17.67
17.68
17.69
17.70
17.71
17.72
17.73
17.74
17.75
17.76

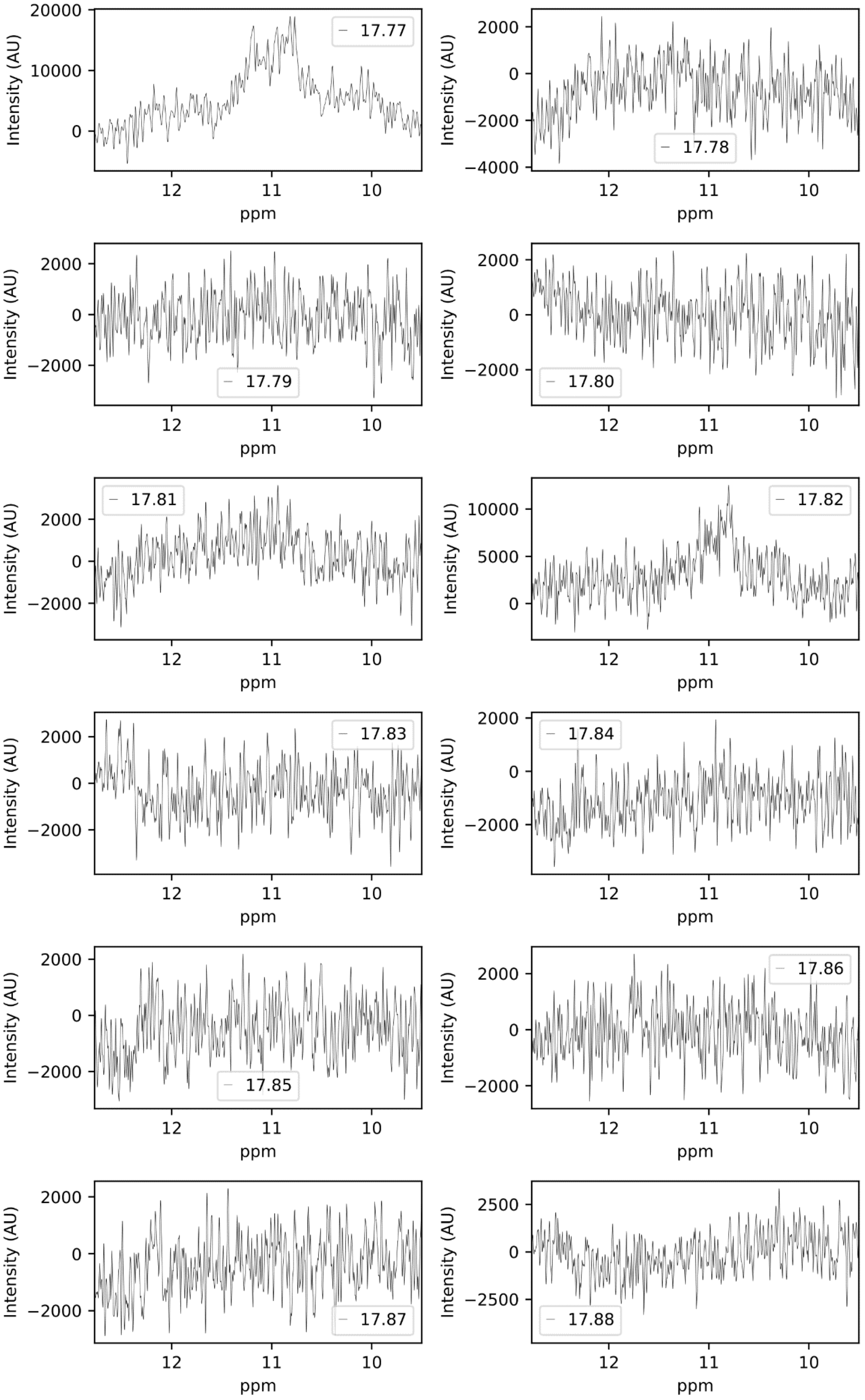
17.77
17.78
17.79
17.80
17.81
17.82
17.83
17.84
17.85
17.86
17.87
17.88
Intensity (AU)
ppm

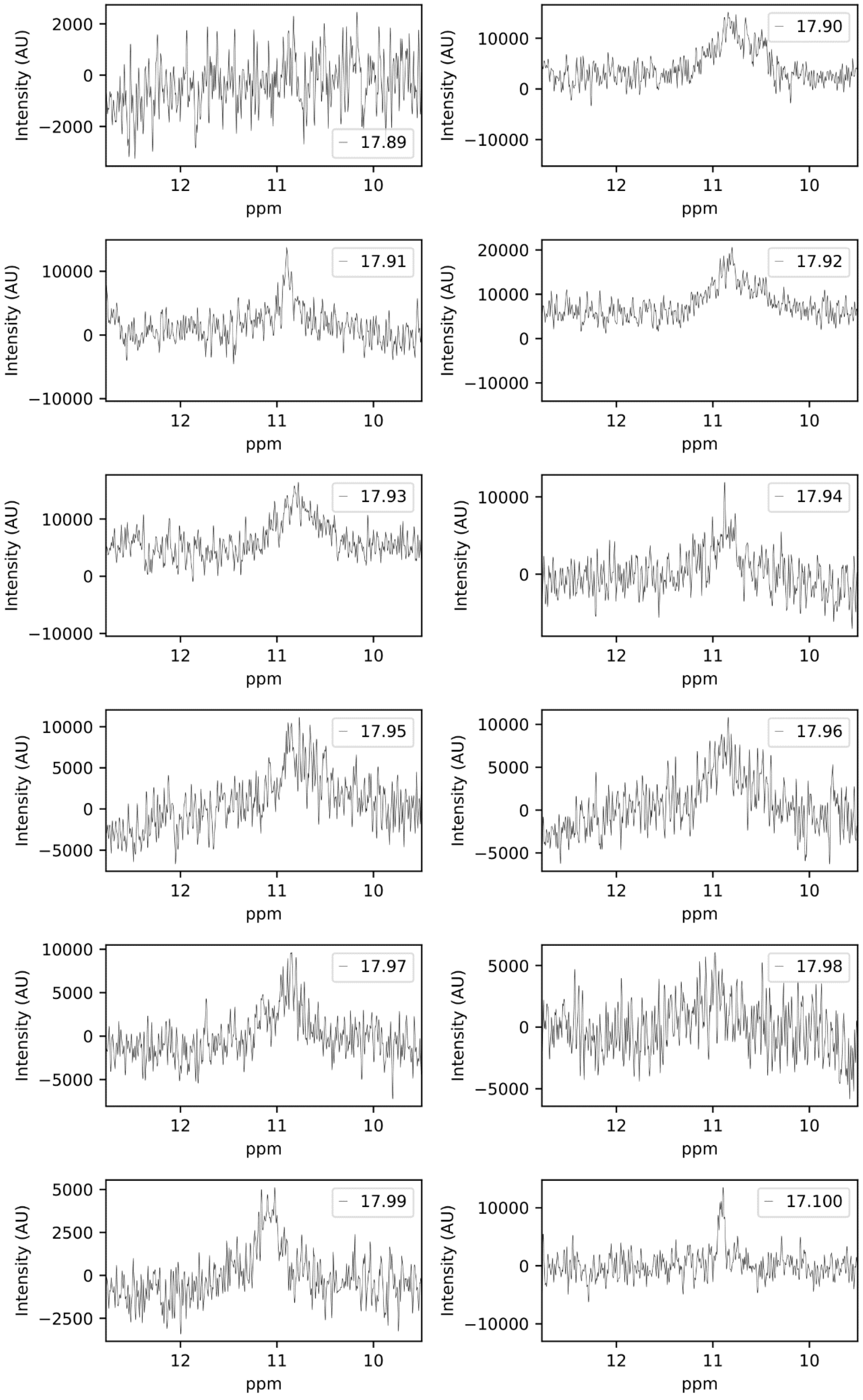
17.89
17.90
17.91
17.92
17.93
17.94
17.95
17.96
17.97
17.98
17.99
17.100
Intensity (AU)
ppm
12
11
10
−10000
−5000
−2500
−2000
0
2000
2500
5000
10000
20000

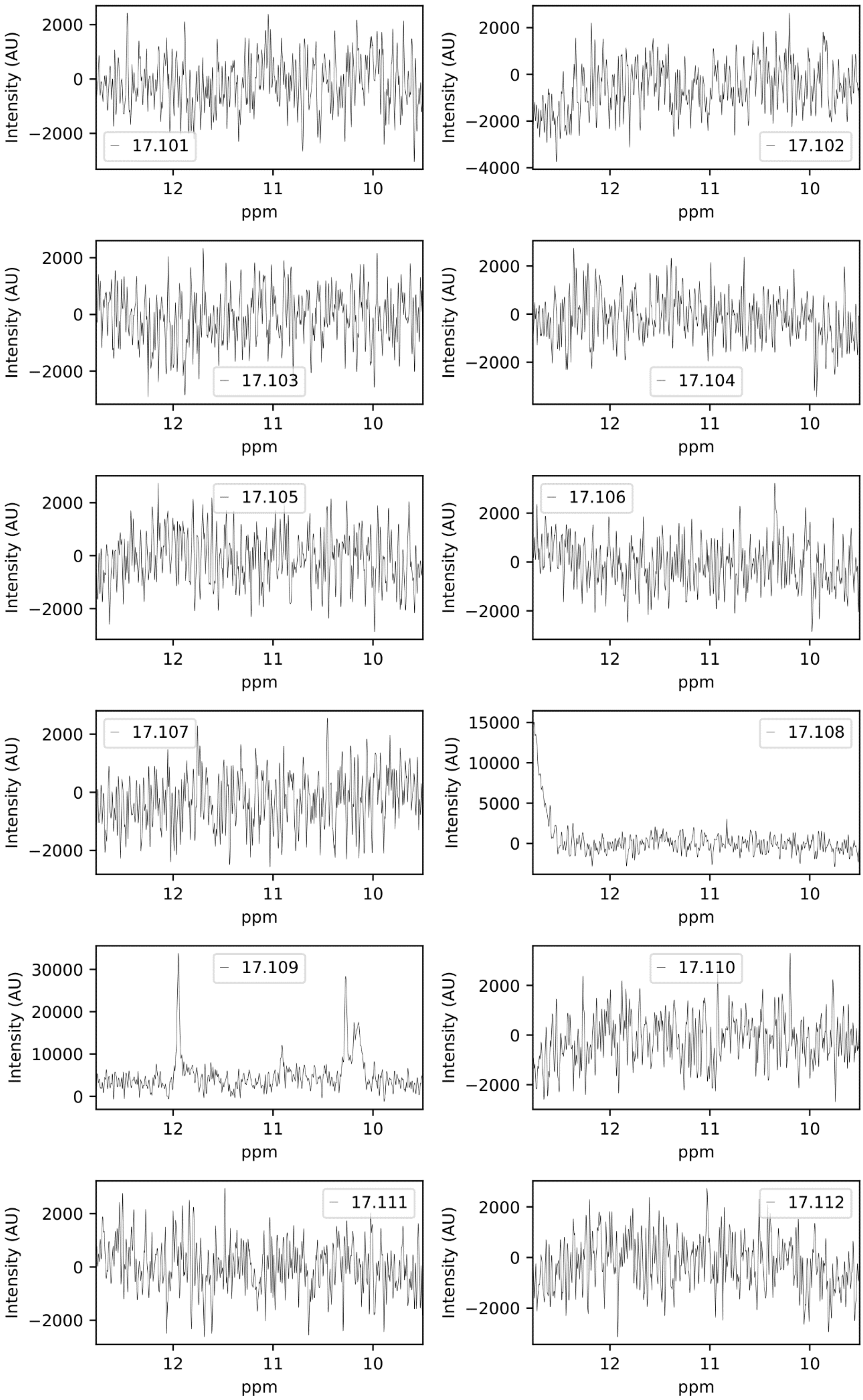
17.101
17.102
17.103
17.104
17.105
17.106
17.107
17.108
17.109
17.110
17.111
17.112
Intensity (AU)
ppm

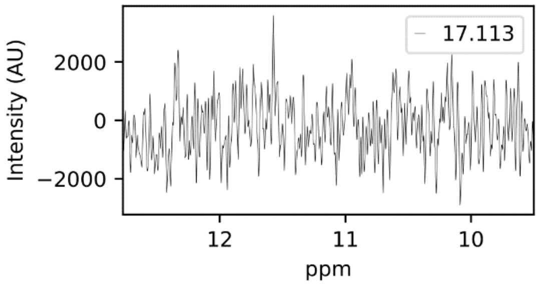
17.113
Intensity (AU)
2000
0
−2000
12
11
10
ppm

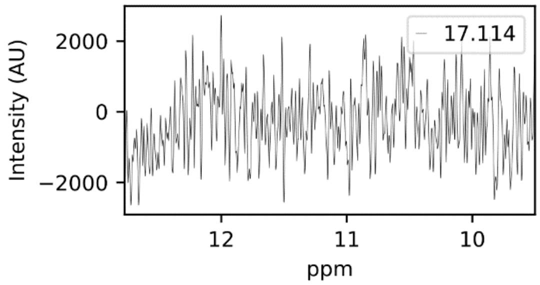
17.114
Intensity (AU)
2000
0
−2000
12
11
10
ppm

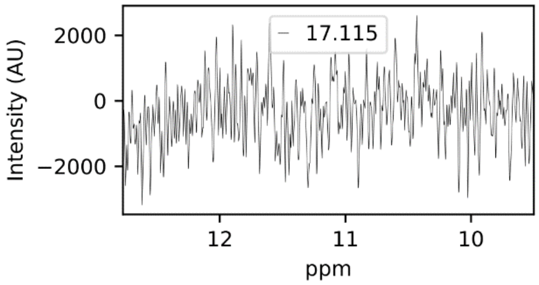
17.115
Intensity (AU)
2000
0
−2000
12
11
10
ppm

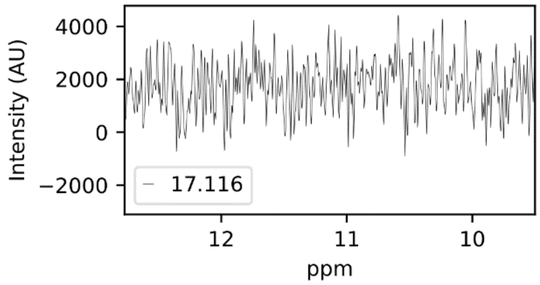
17.116
Intensity (AU)
4000
2000
0
−2000
12
11
10
ppm

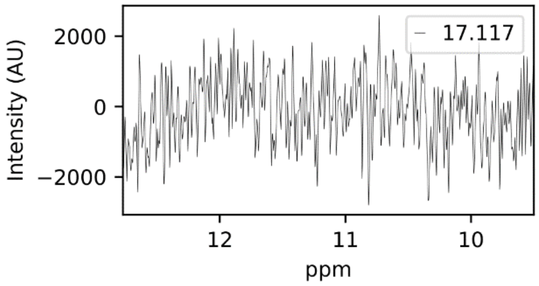
17.117
Intensity (AU)
2000
0
−2000
12
11
10
ppm

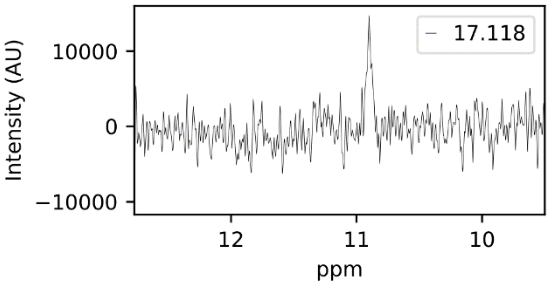
17.118
Intensity (AU)
10000
0
−10000
12
11
10
ppm

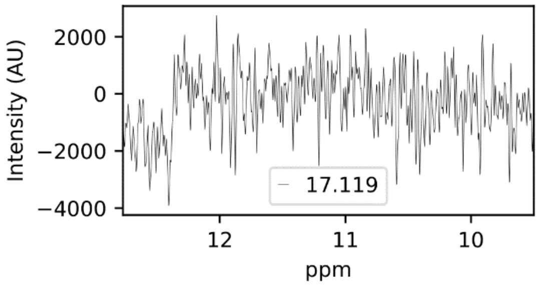
17.119
Intensity (AU)
2000
0
−2000
−4000
12
11
10
ppm

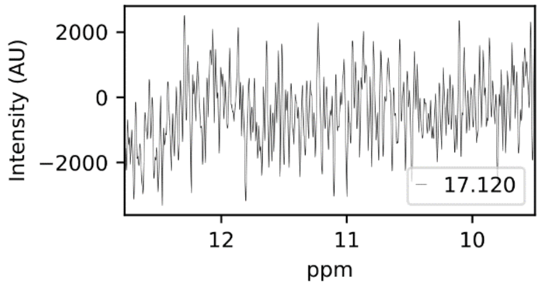
17.120
Intensity (AU)
2000
0
−2000
12
11
10
ppm

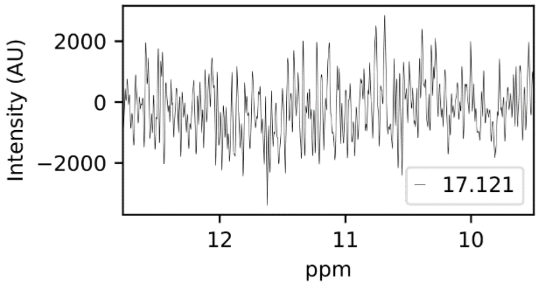
17.121
Intensity (AU)
2000
0
−2000
12
11
10
ppm

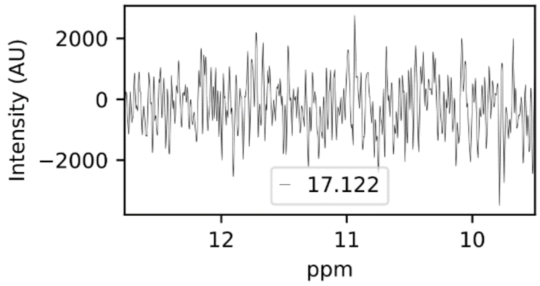
17.122
Intensity (AU)
2000
0
−2000
12
11
10
ppm

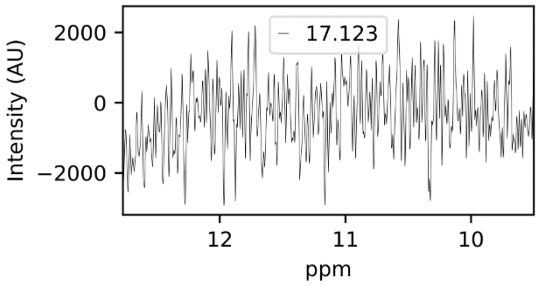
17.123
Intensity (AU)
2000
0
−2000
12
11
10
ppm

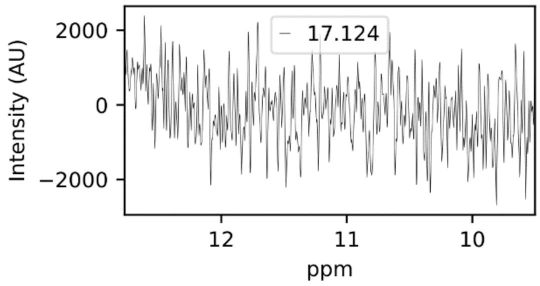
17.124
Intensity (AU)
2000
0
−2000
12
11
10
ppm

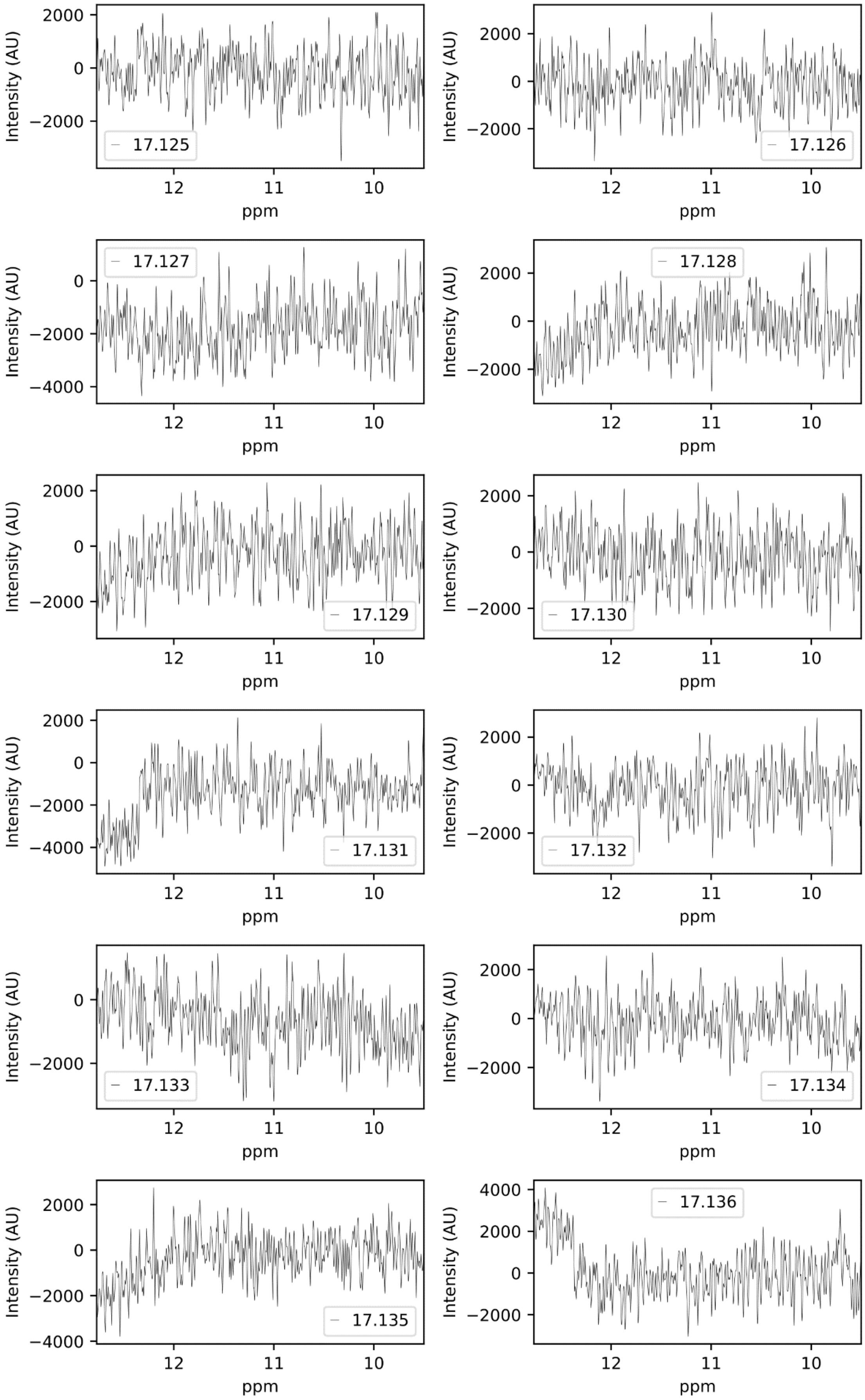
Intensity (AU)
ppm
17.125
17.126
17.127
17.128
17.129
17.130
17.131
17.132
17.133
17.134
17.135
17.136

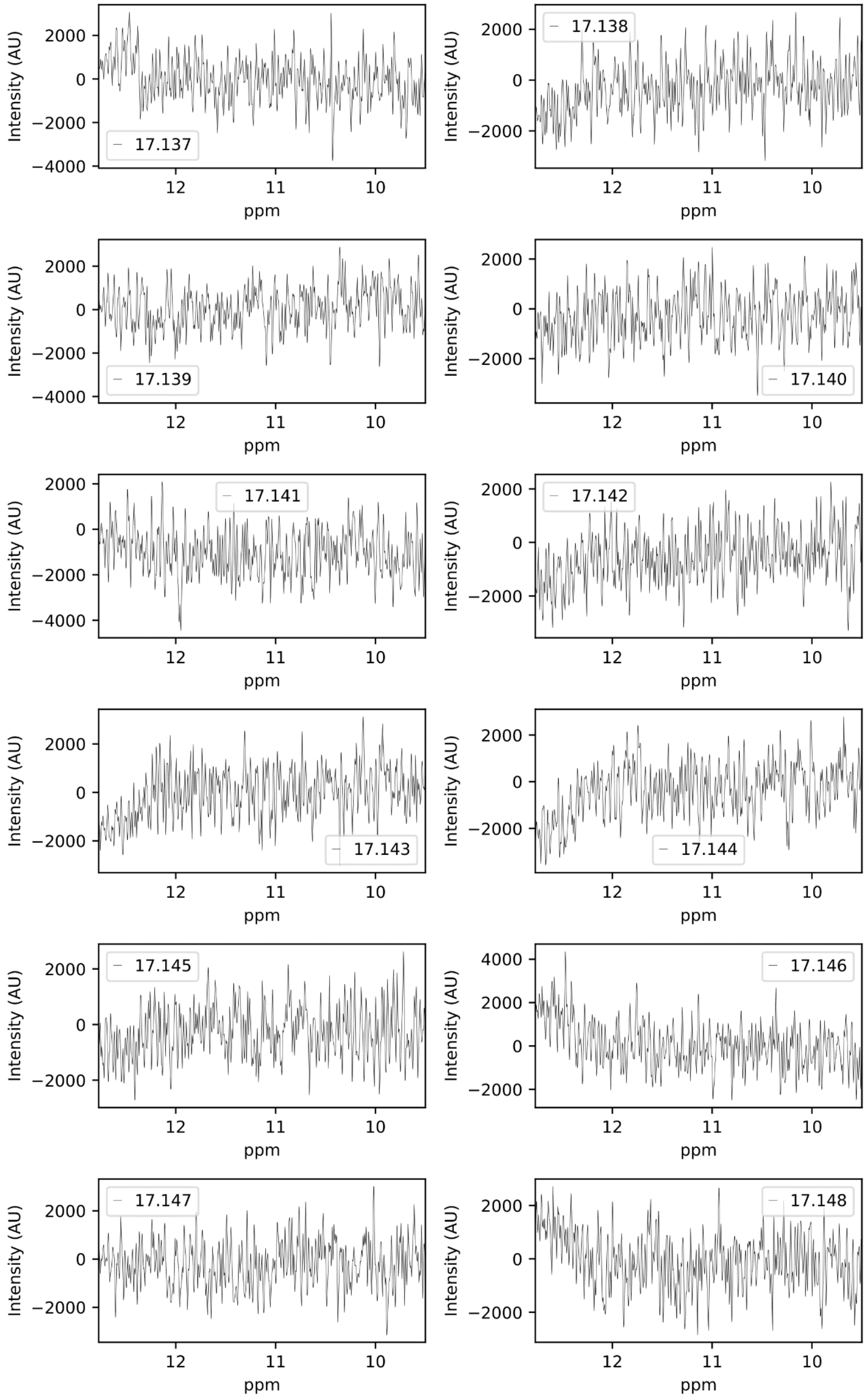
17.137
17.138
17.139
17.140
17.141
17.142
17.143
17.144
17.145
17.146
17.147
17.148
Intensity (AU)
ppm
12
11
10

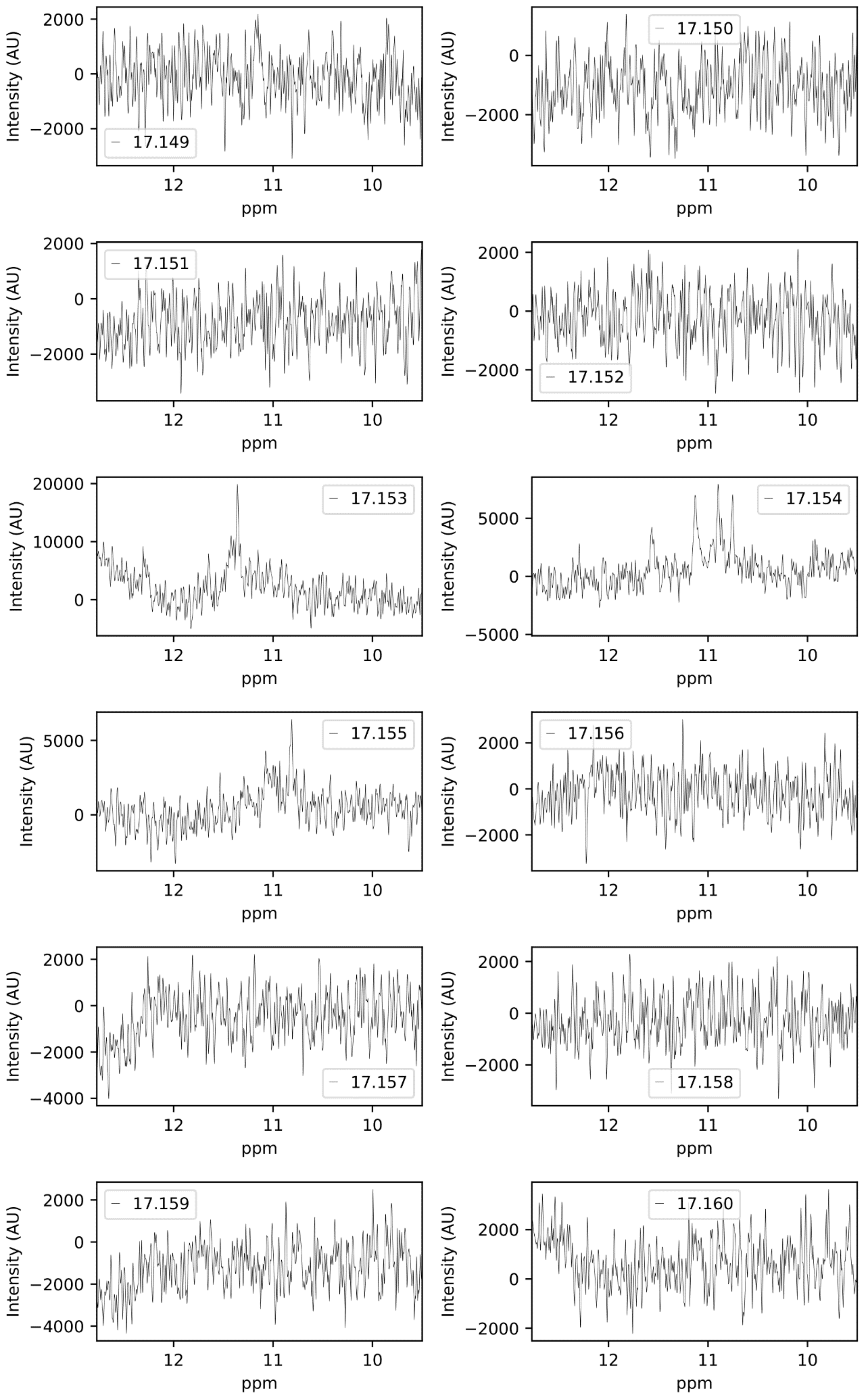

17.149
17.150
17.151
17.152
17.153
17.154
17.155
17.156
17.157
17.158
17.159
17.160
Intensity (AU)
ppm
12
11
10

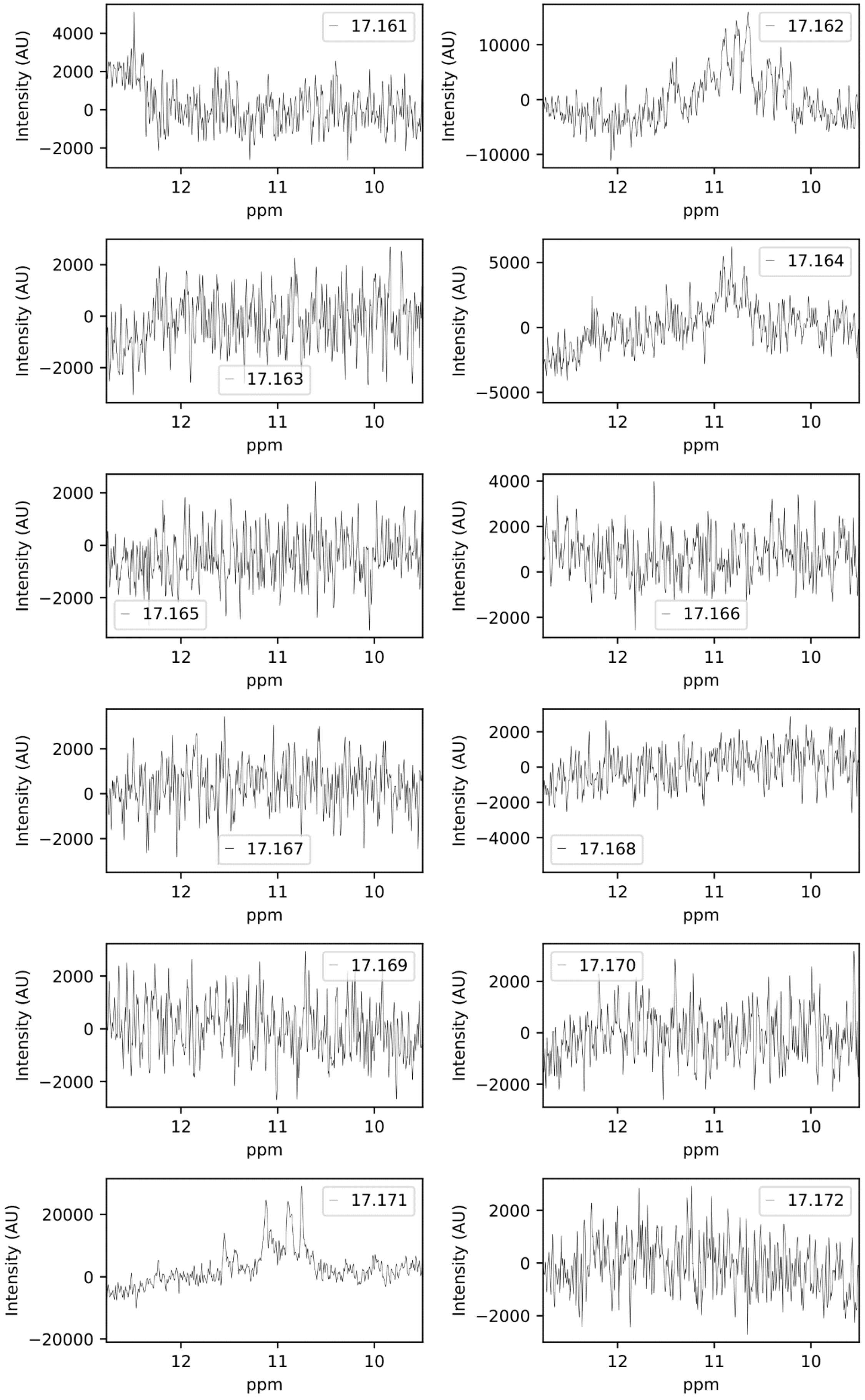

17.161
17.162
17.163
17.164
17.165
17.166
17.167
17.168
17.169
17.170
17.171
17.172
Intensity (AU)
ppm

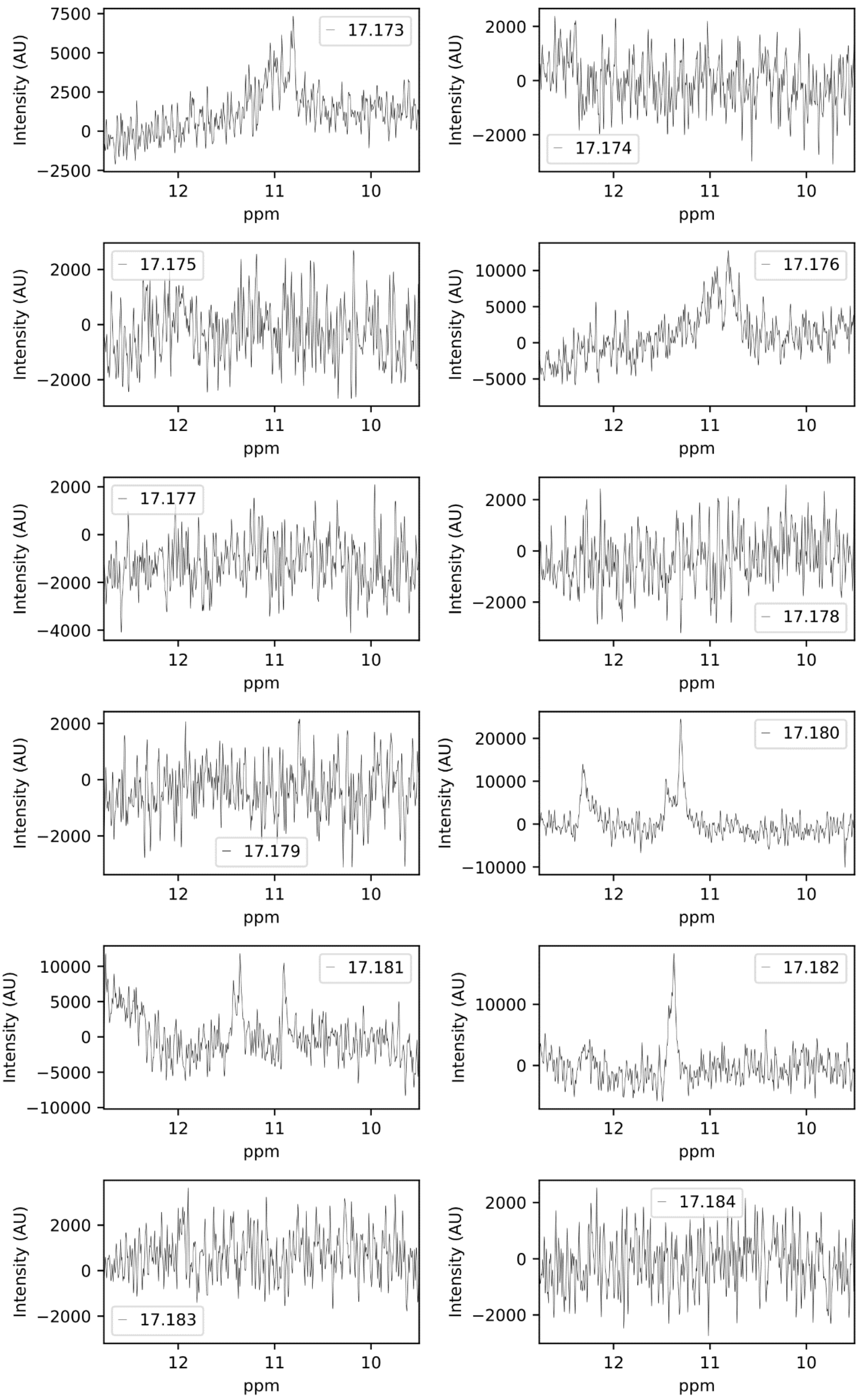
17.173
17.174
17.175
17.176
17.177
17.178
17.179
17.180
17.181
17.182
17.183
17.184
Intensity (AU)
ppm

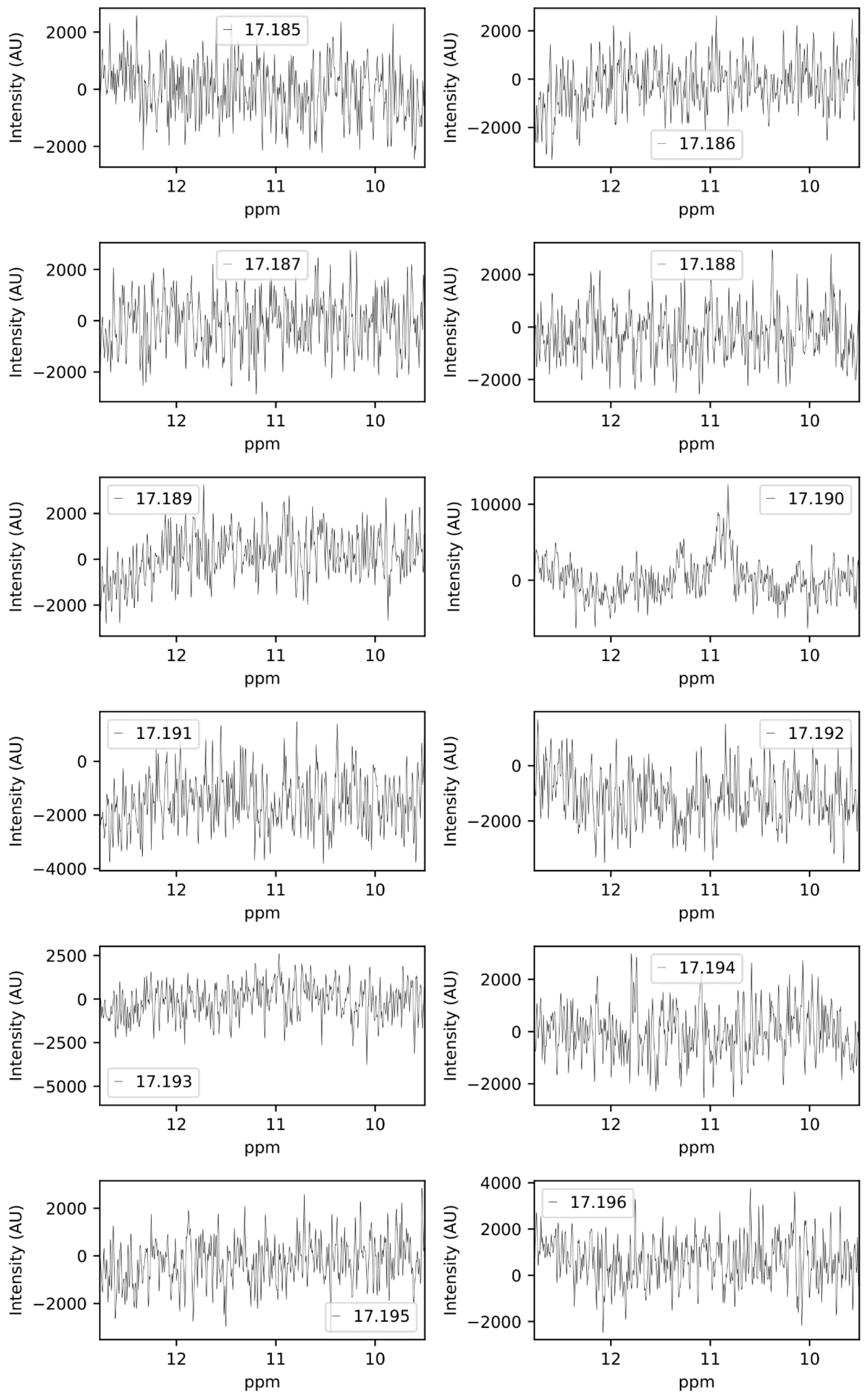
17.185
17.186
17.187
17.188
17.189
17.190
17.191
17.192
17.193
17.194
17.195
17.196
Intensity (AU)
ppm

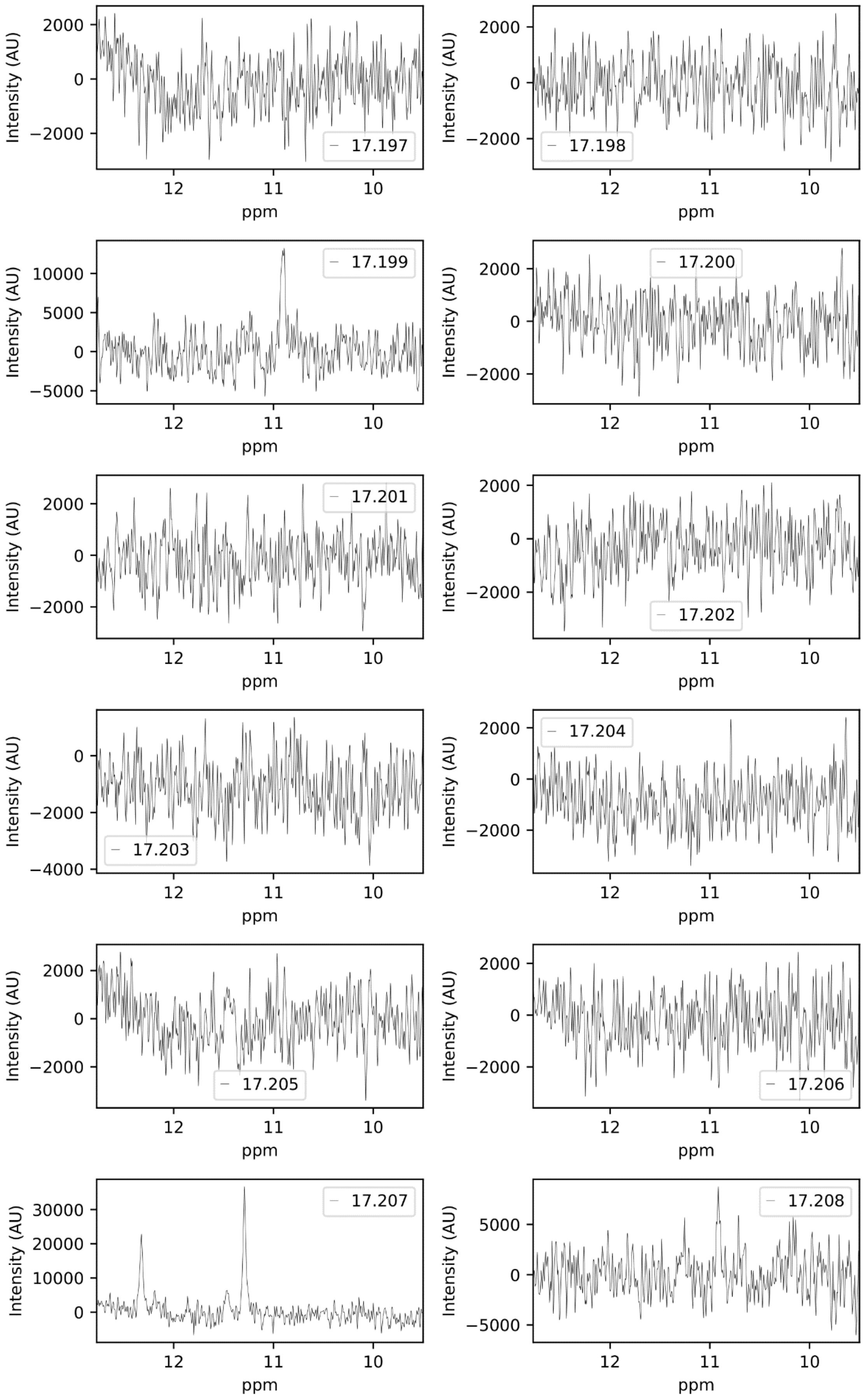

17.197
17.198
17.199
17.200
17.201
17.202
17.203
17.204
17.205
17.206
17.207
17.208
Intensity (AU)
ppm

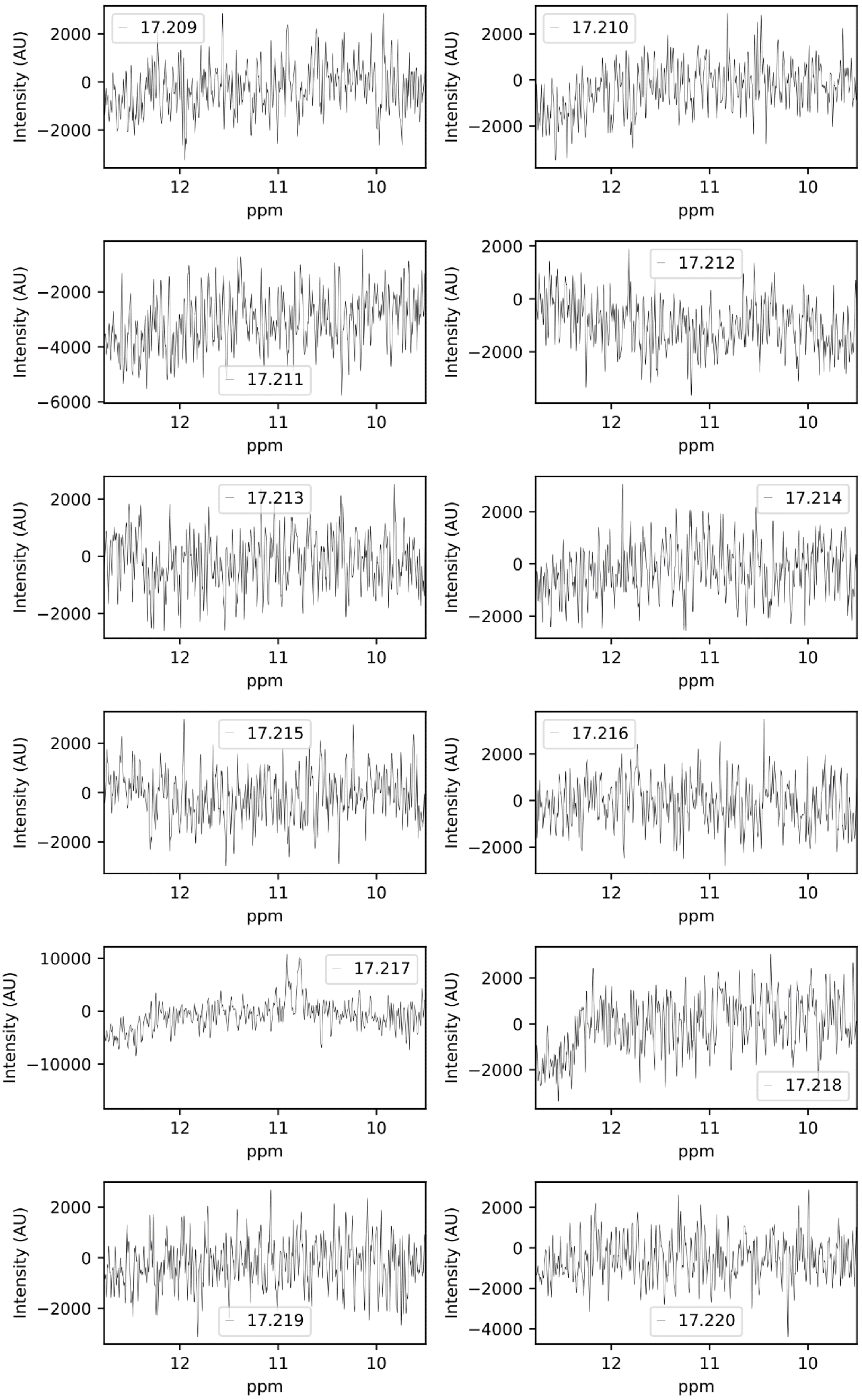
17.209
17.210
17.211
17.212
17.213
17.214
17.215
17.216
17.217
17.218
17.219
17.220
Intensity (AU)
ppm

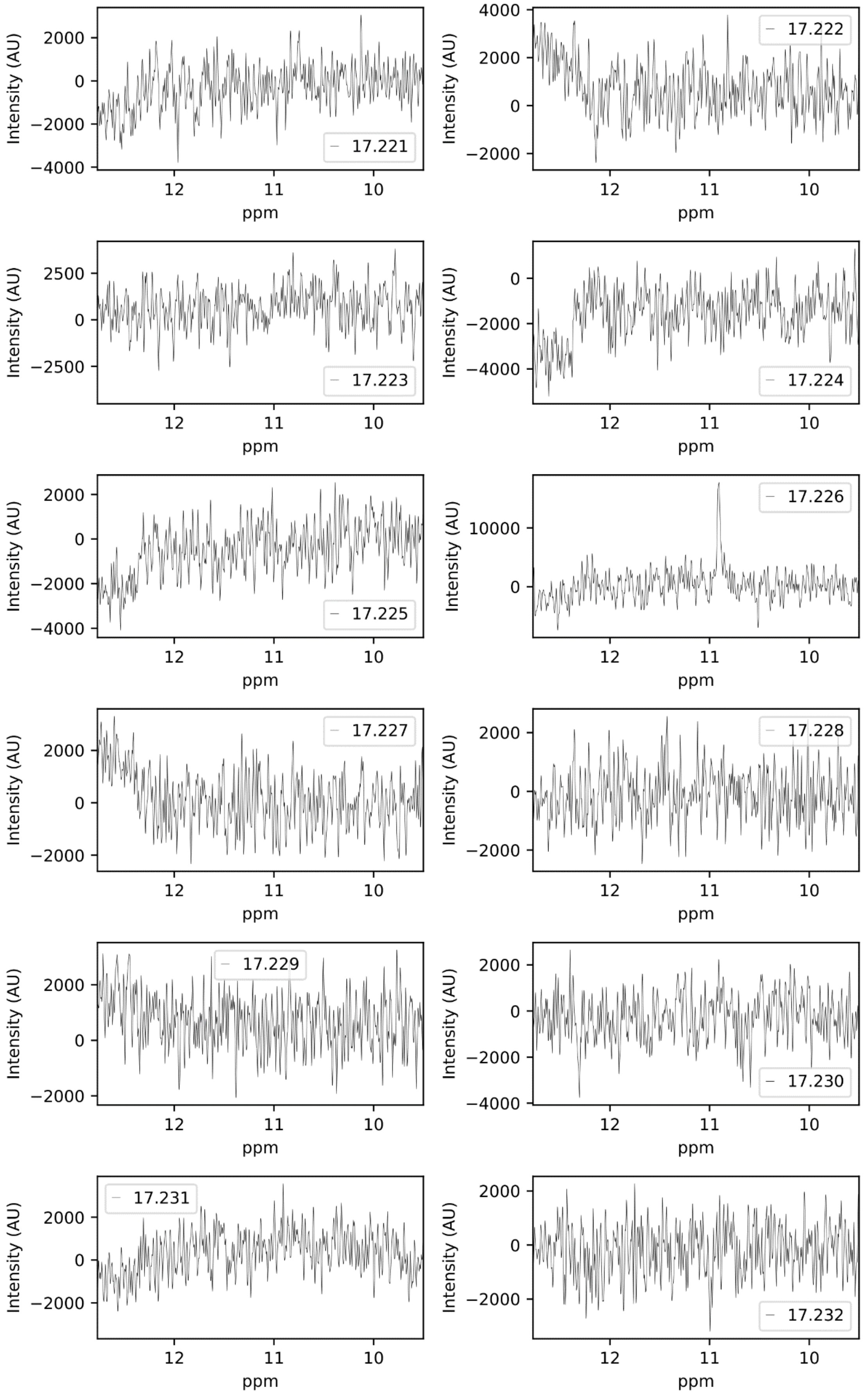
Intensity (AU)
ppm
17.221
17.222
17.223
17.224
17.225
17.226
17.227
17.228
17.229
17.230
17.231
17.232

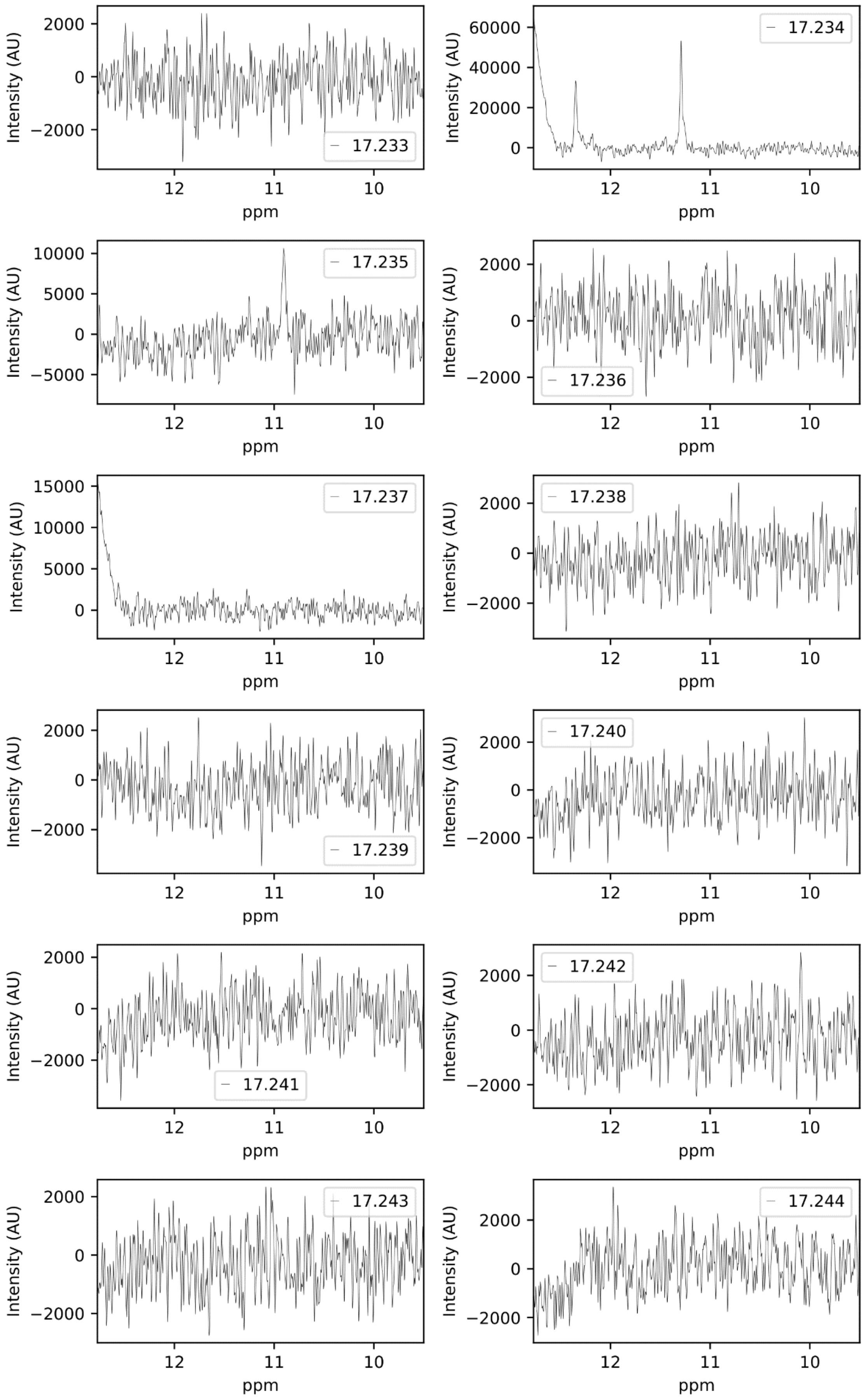
17.233
17.234
17.235
17.236
17.237
17.238
17.239
17.240
17.241
17.242
17.243
17.244
Intensity (AU)
ppm

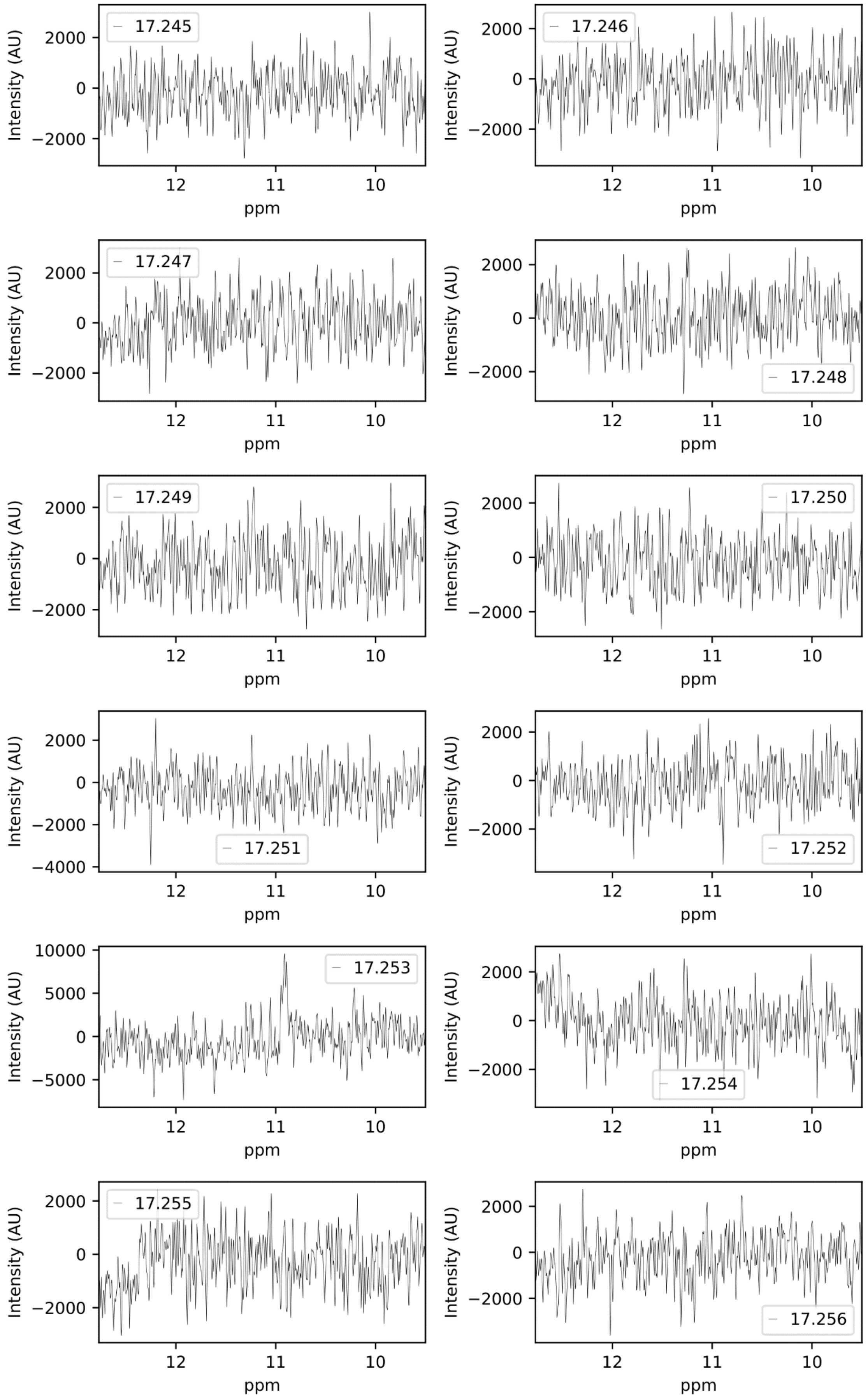
17.245
17.246
17.247
17.248
17.249
17.250
17.251
17.252
17.253
17.254
17.255
17.256
Intensity (AU)
ppm

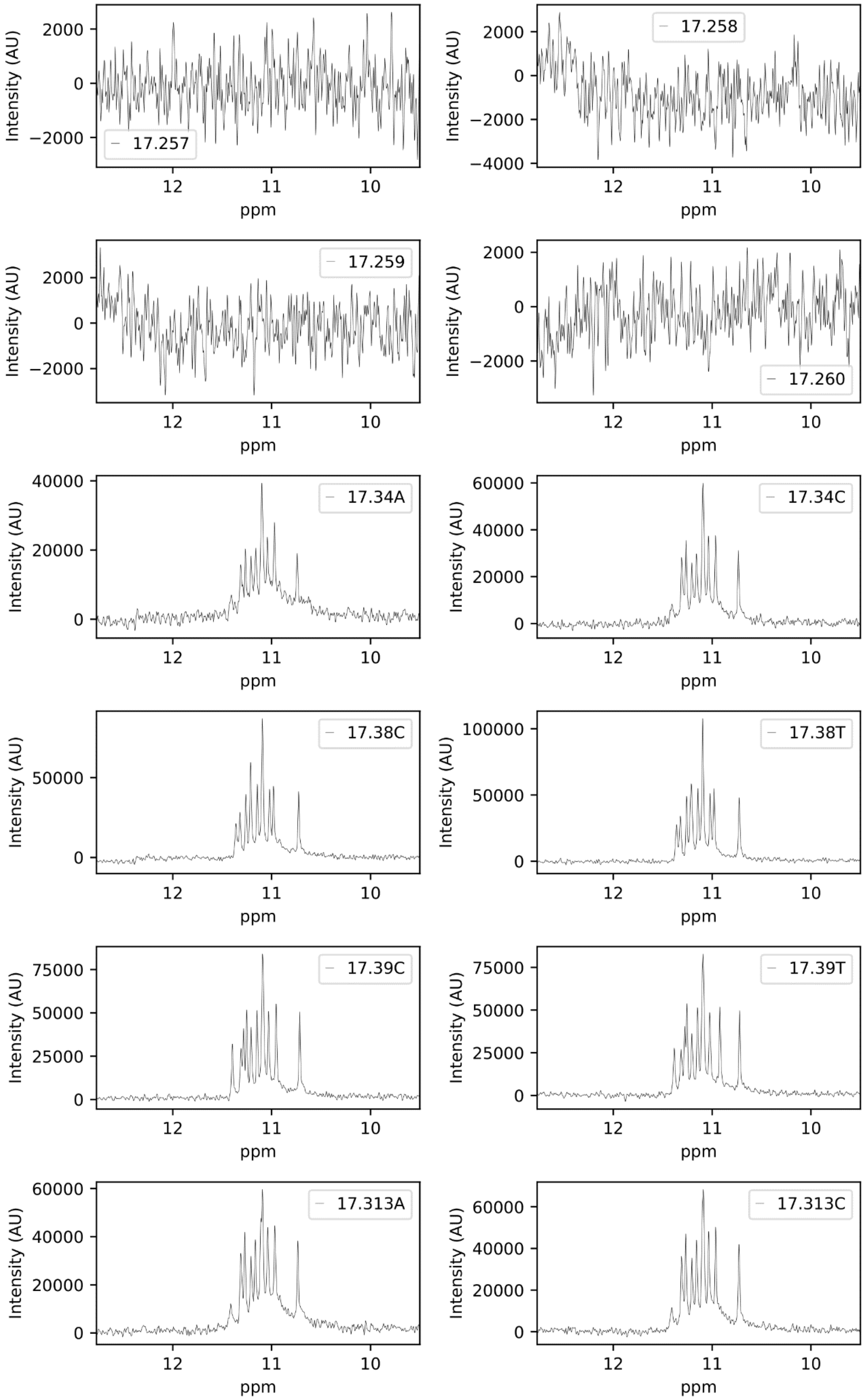
17.257
17.258
17.259
17.260
17.34A
17.34C
17.38C
17.38T
17.39C
17.39T
17.313A
17.313C
Intensity (AU)
ppm
12
11
10

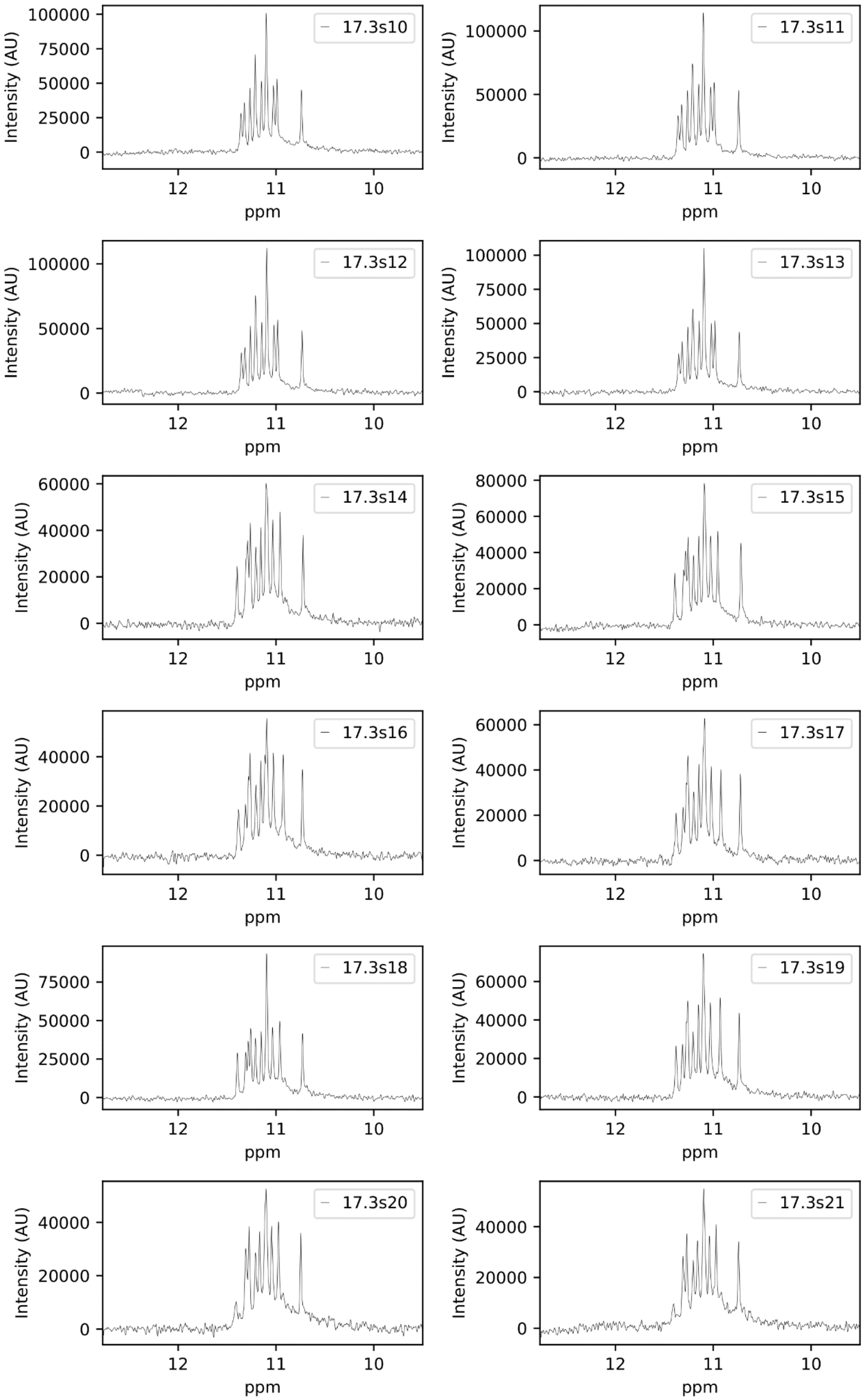
17.3s10
17.3s11
17.3s12
17.3s13
17.3s14
17.3s15
17.3s16
17.3s17
17.3s18
17.3s19
17.3s20
17.3s21
Intensity (AU)
ppm

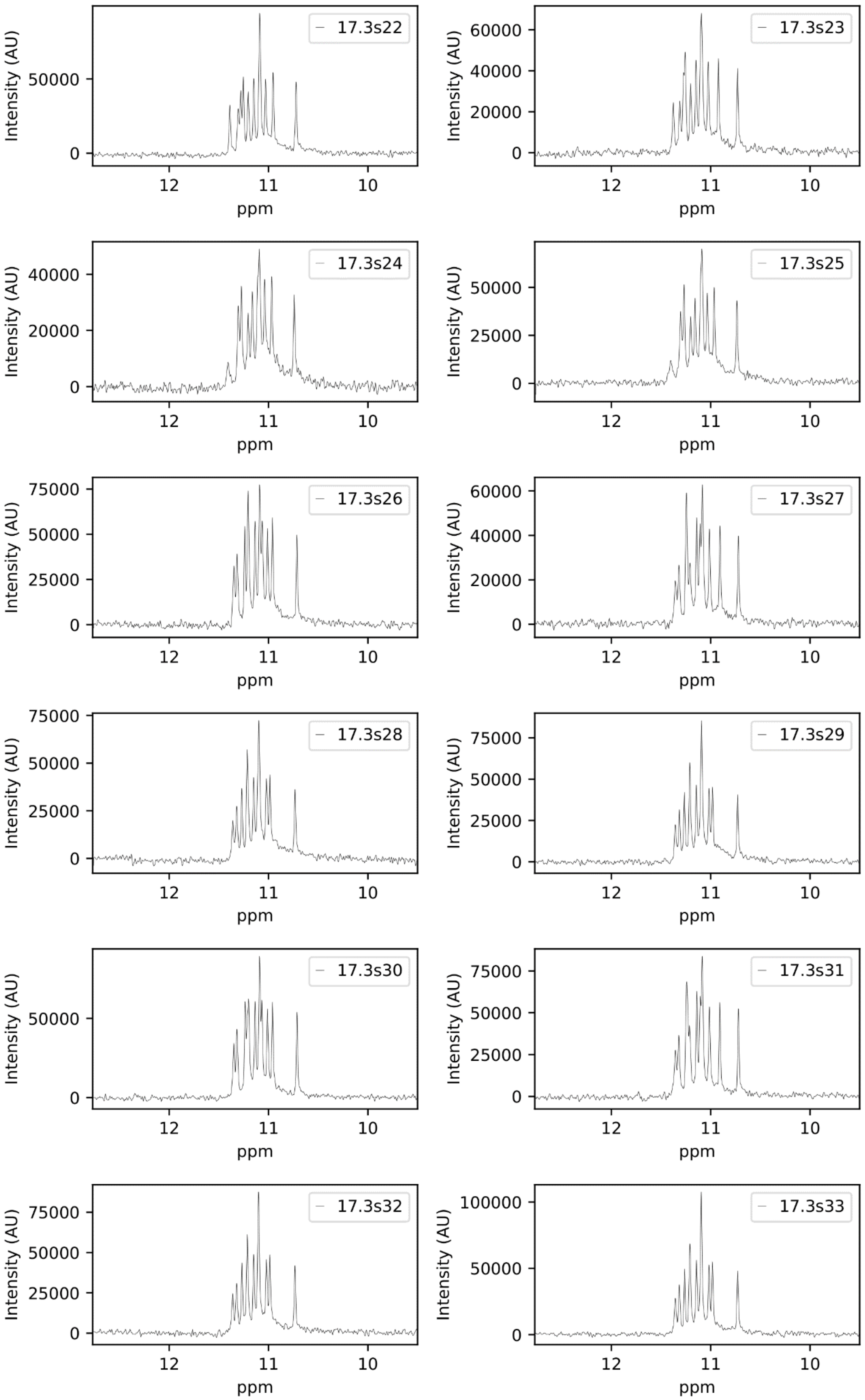
17.3s22
17.3s23
17.3s24
17.3s25
17.3s26
17.3s27
17.3s28
17.3s29
17.3s30
17.3s31
17.3s32
17.3s33
Intensity (AU)
ppm

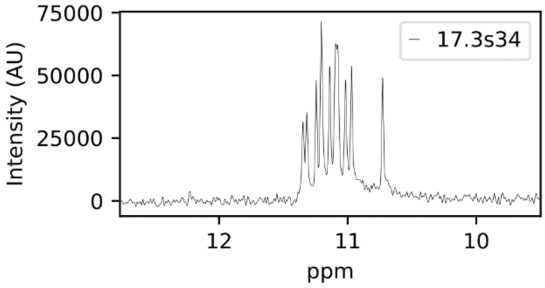
17.3s34
Intensity (AU)
75000
50000
25000
0
12
11
10
ppm

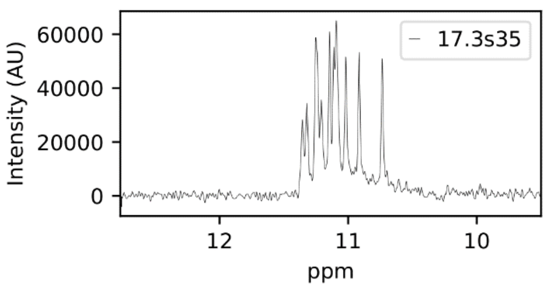
17.3s35
Intensity (AU)
60000
40000
20000
0
12
11
10
ppm

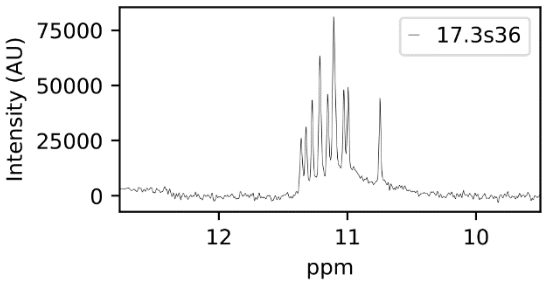
17.3s36
Intensity (AU)
75000
50000
25000
0
12
11
10
ppm

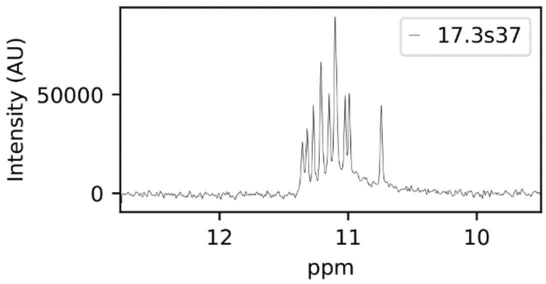
17.3s37
Intensity (AU)
50000
0
12
11
10
ppm

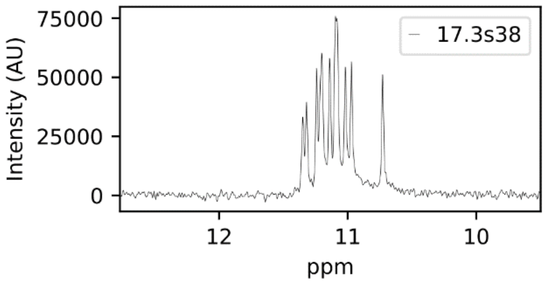
17.3s38
Intensity (AU)
75000
50000
25000
0
12
11
10
ppm

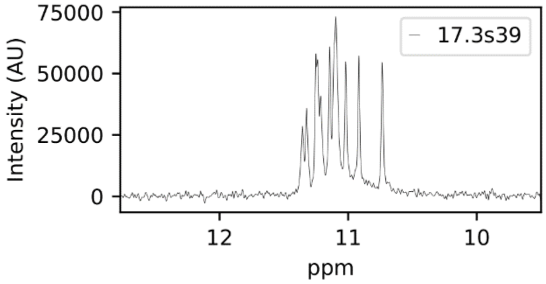
17.3s39
Intensity (AU)
75000
50000
25000
0
12
11
10
ppm

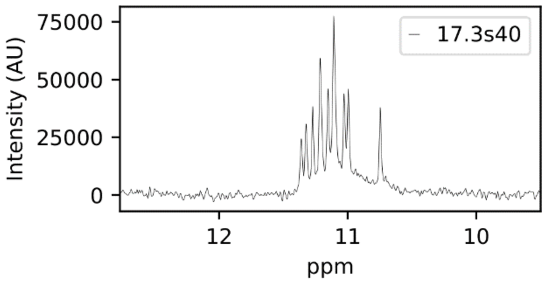
17.3s40
Intensity (AU)
75000
50000
25000
0
12
11
10
ppm

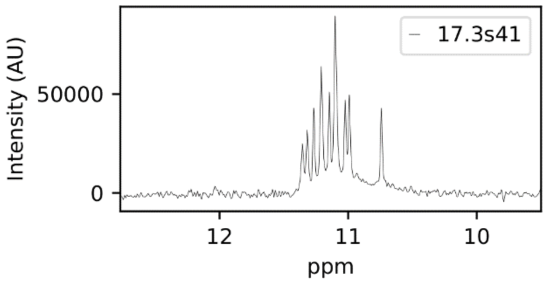
17.3s41
Intensity (AU)
50000
0
12
11
10
ppm

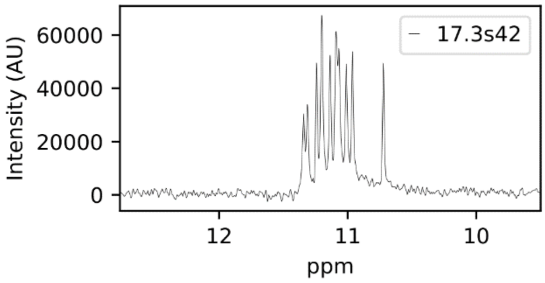
17.3s42
Intensity (AU)
60000
40000
20000
0
12
11
10
ppm

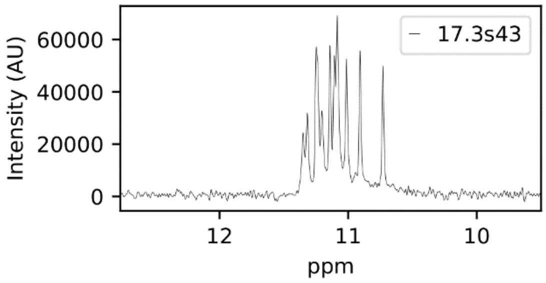
17.3s43
Intensity (AU)
60000
40000
20000
0
12
11
10
ppm

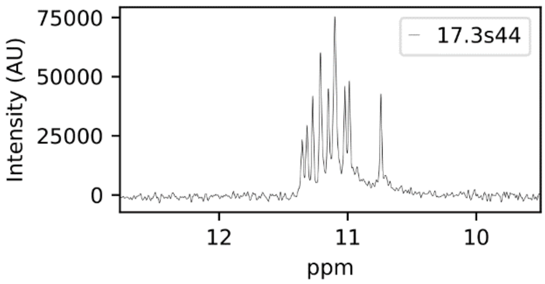
17.3s44
Intensity (AU)
75000
50000
25000
0
12
11
10
ppm

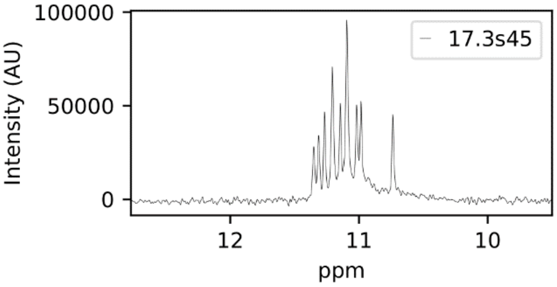
17.3s45
Intensity (AU)
100000
50000
0
12
11
10
ppm

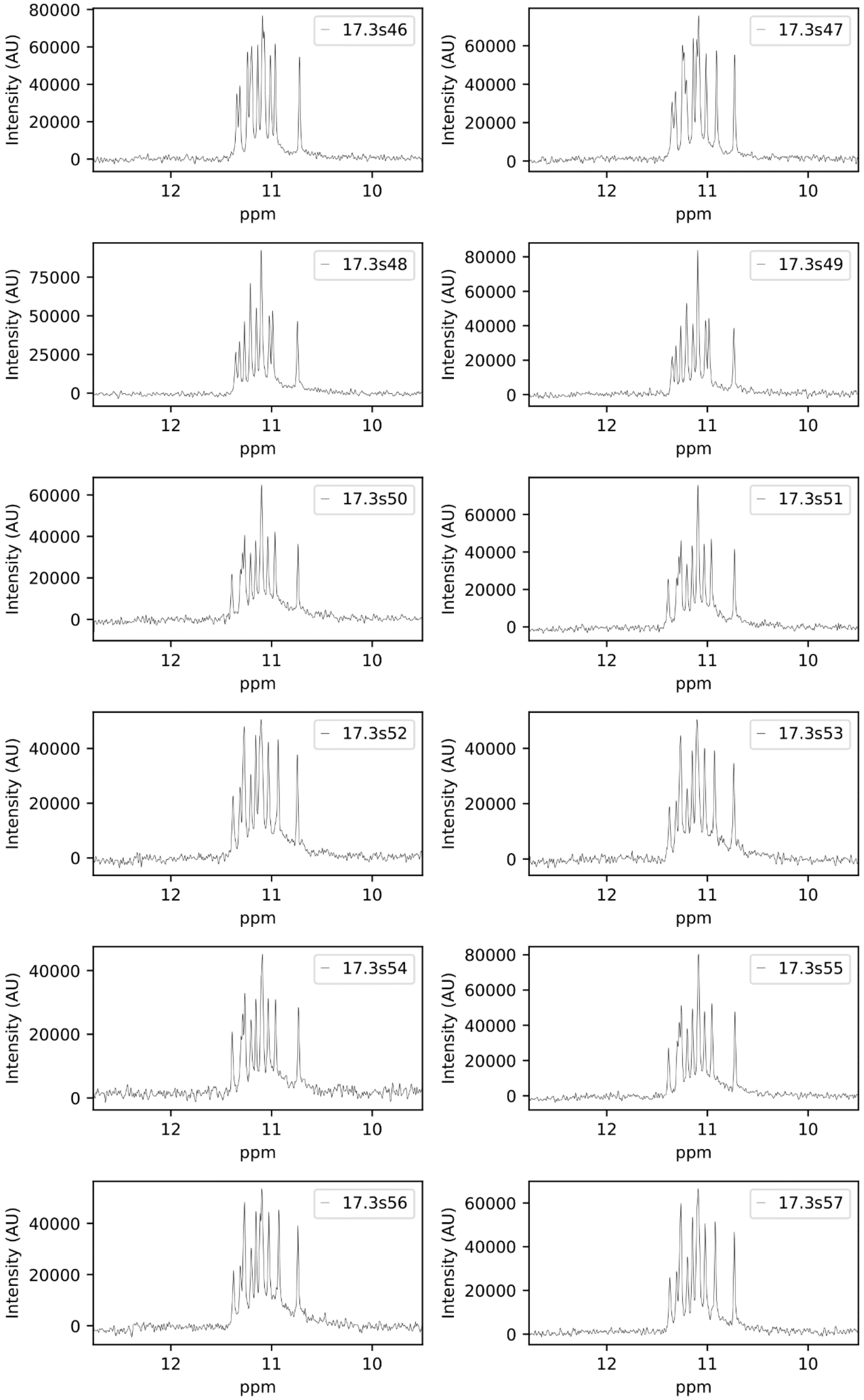
17.3s46
17.3s47
17.3s48
17.3s49
17.3s50
17.3s51
17.3s52
17.3s53
17.3s54
17.3s55
17.3s56
17.3s57
Intensity (AU)
ppm

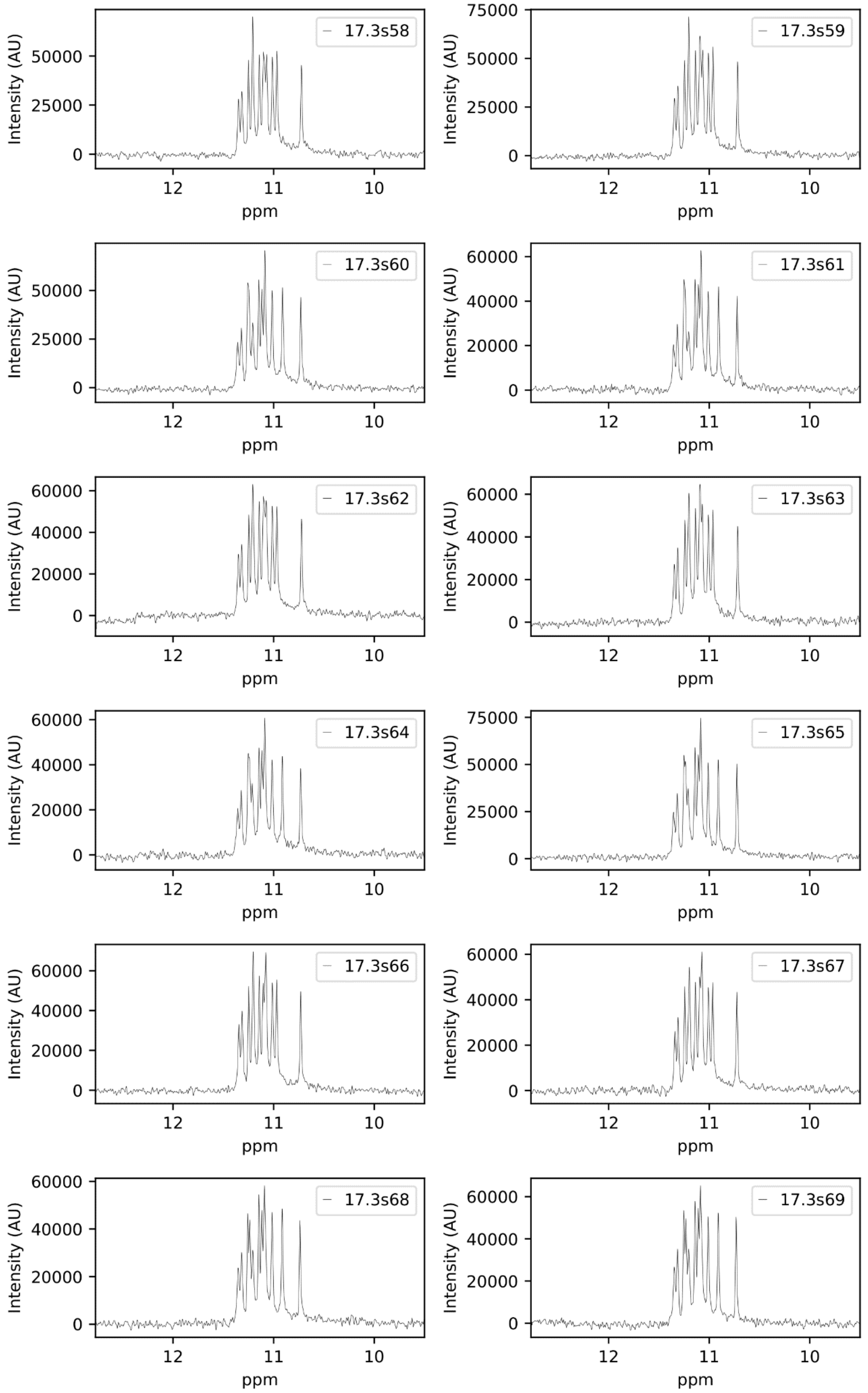
17.3s58
17.3s59
17.3s60
17.3s61
17.3s62
17.3s63
17.3s64
17.3s65
17.3s66
17.3s67
17.3s68
17.3s69
Intensity (AU)
ppm
12
11
10

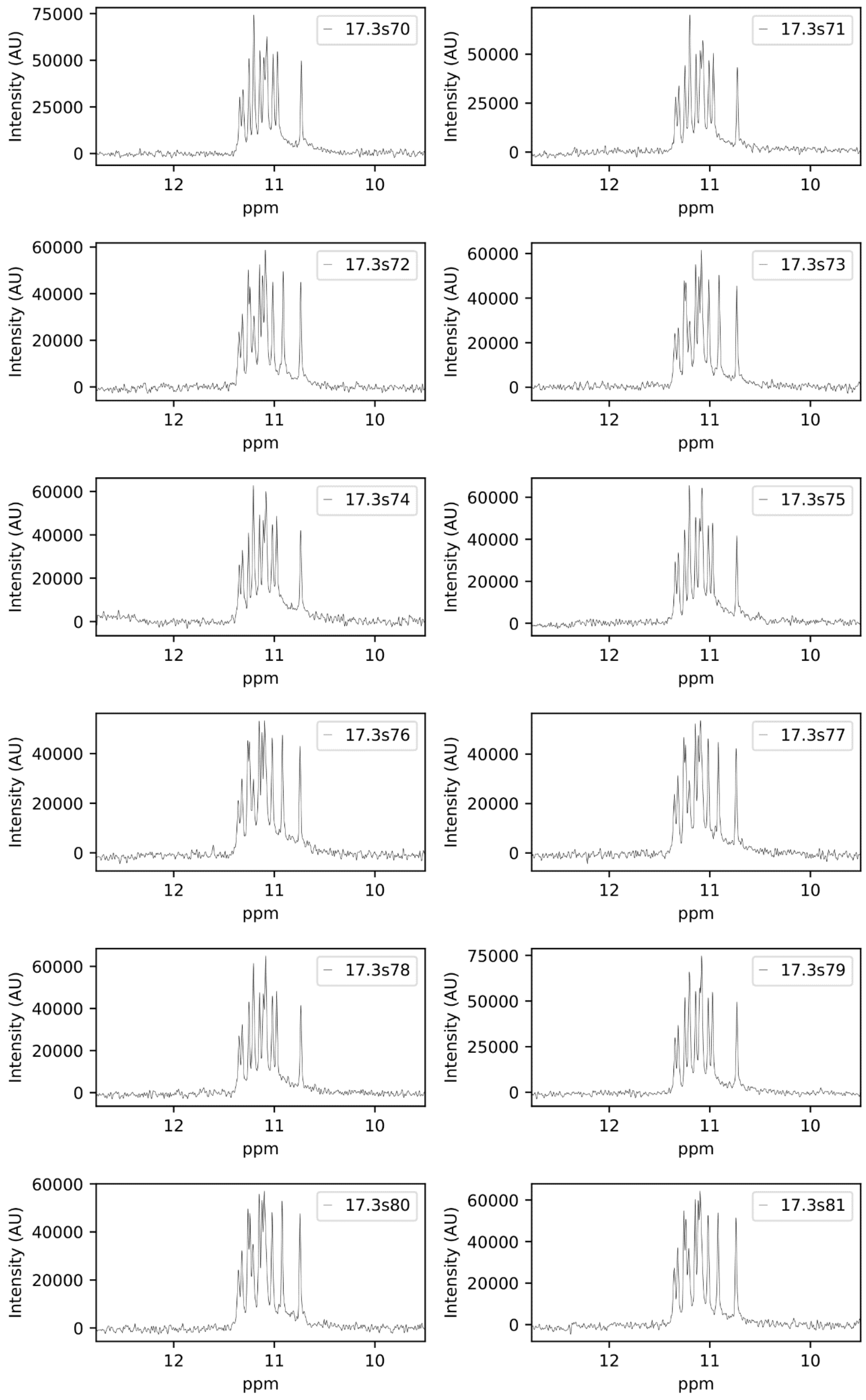
17.3s70
17.3s71
17.3s72
17.3s73
17.3s74
17.3s75
17.3s76
17.3s77
17.3s78
17.3s79
17.3s80
17.3s81
Intensity (AU)
ppm
12
11
10

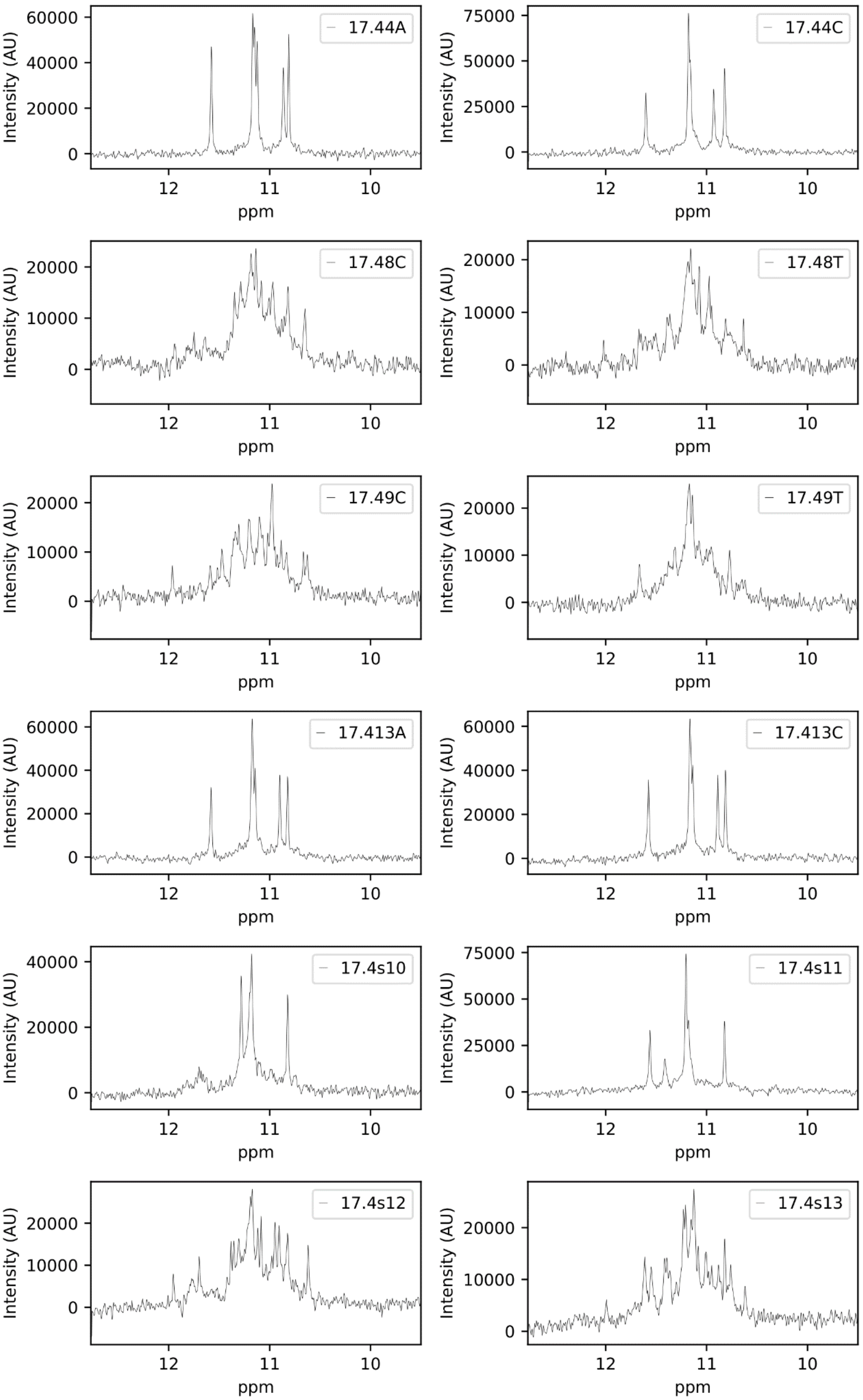
17.44A
17.44C
17.48C
17.48T
17.49C
17.49T
17.413A
17.413C
17.4s10
17.4s11
17.4s12
17.4s13
Intensity (AU)
ppm
12
11
10
0
10000
20000
25000
40000
50000
60000
75000

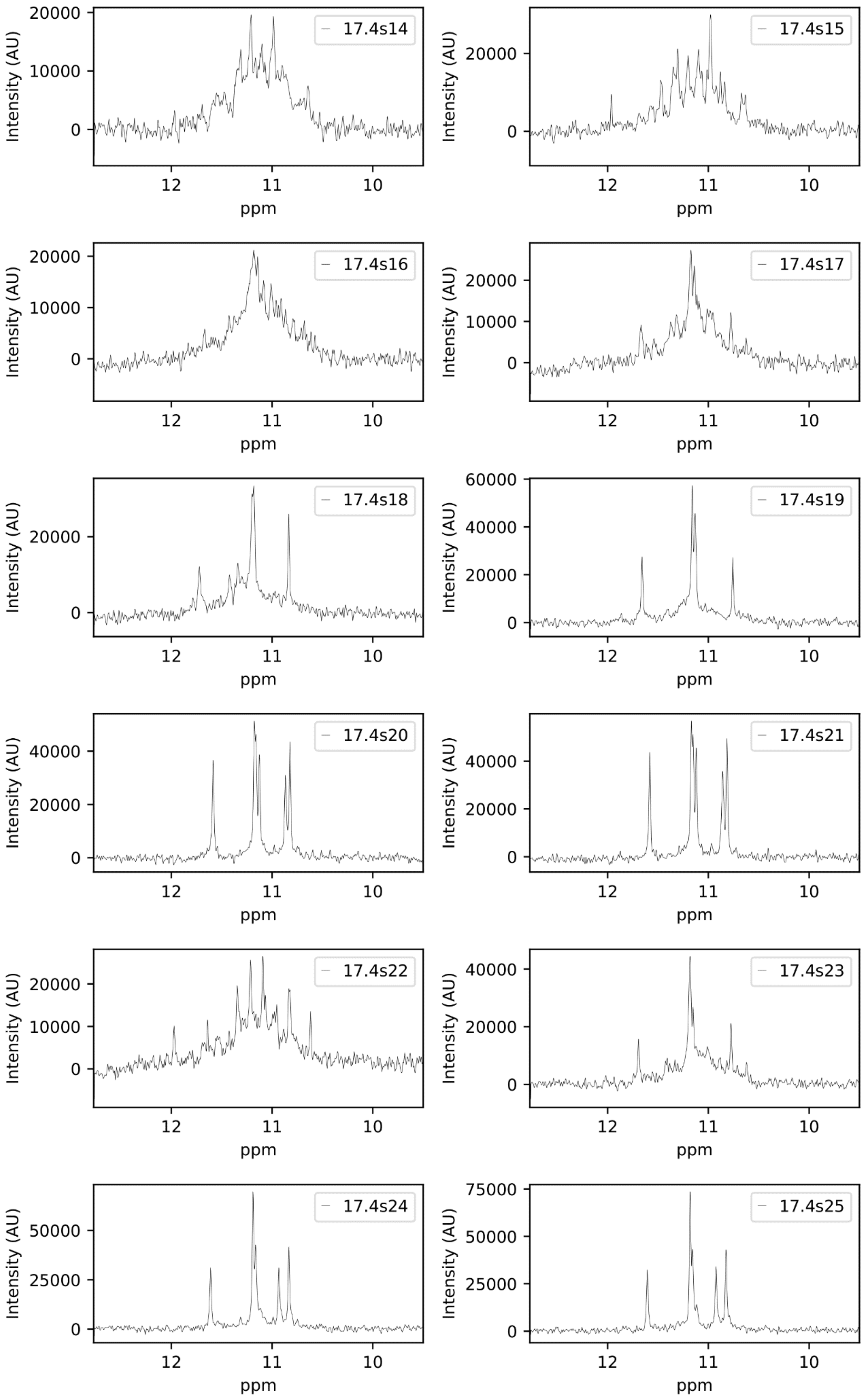
17.4s14
17.4s15
17.4s16
17.4s17
17.4s18
17.4s19
17.4s20
17.4s21
17.4s22
17.4s23
17.4s24
17.4s25
Intensity (AU)
ppm

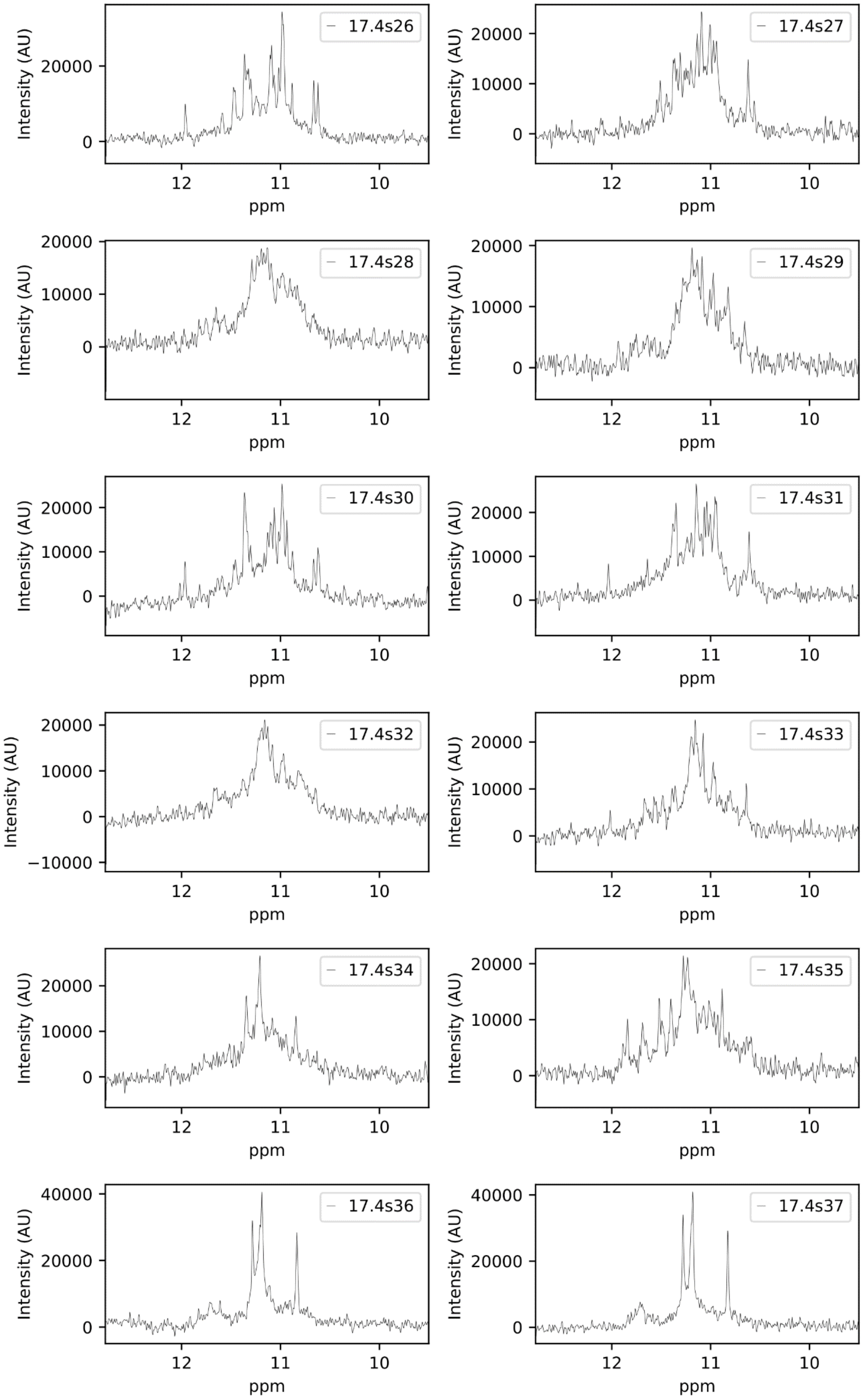
17.4s26
17.4s27
17.4s28
17.4s29
17.4s30
17.4s31
17.4s32
17.4s33
17.4s34
17.4s35
17.4s36
17.4s37
Intensity (AU)
ppm
12
11
10

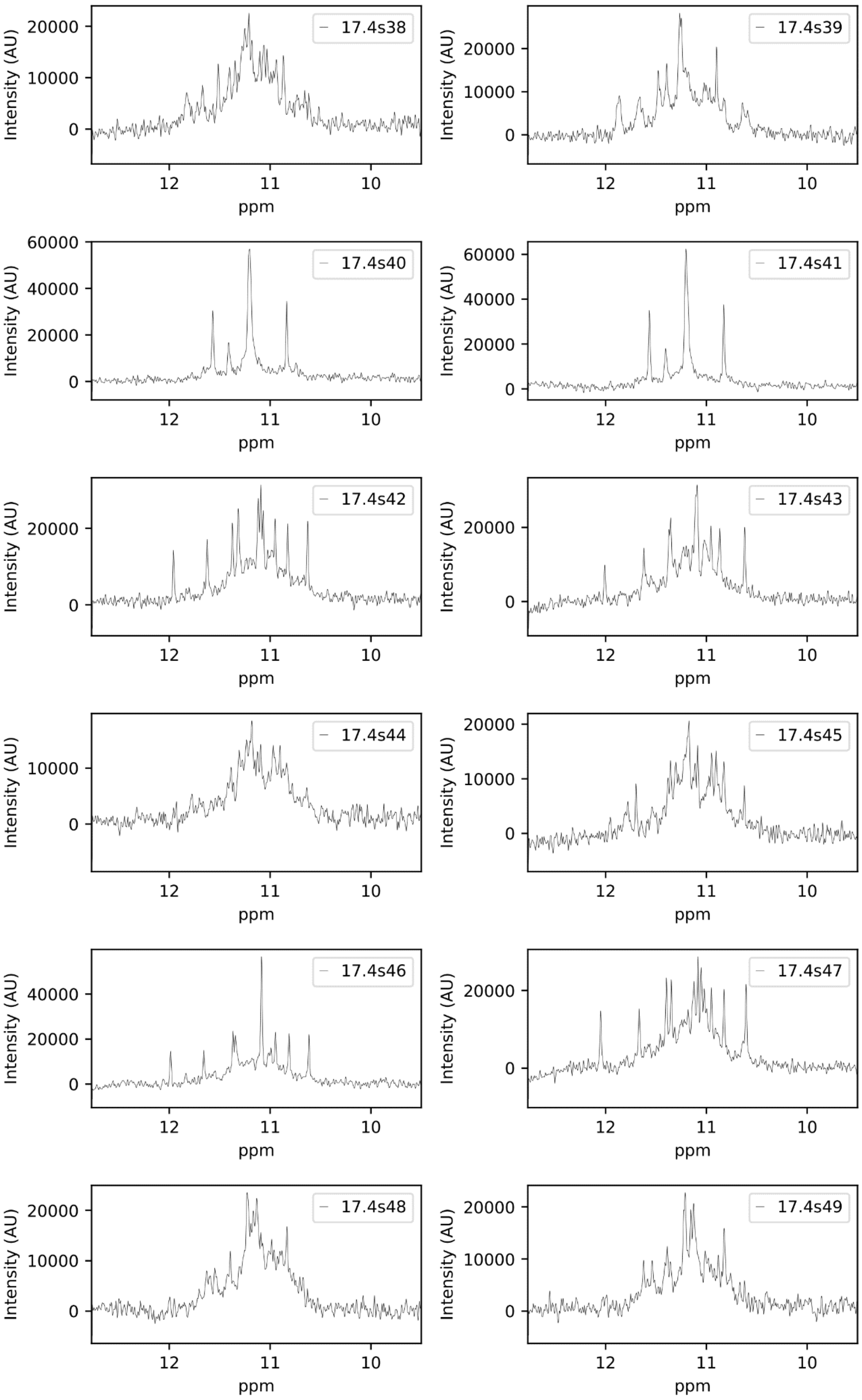
17.4s38
17.4s39
17.4s40
17.4s41
17.4s42
17.4s43
17.4s44
17.4s45
17.4s46
17.4s47
17.4s48
17.4s49
Intensity (AU)
ppm
12
11
10

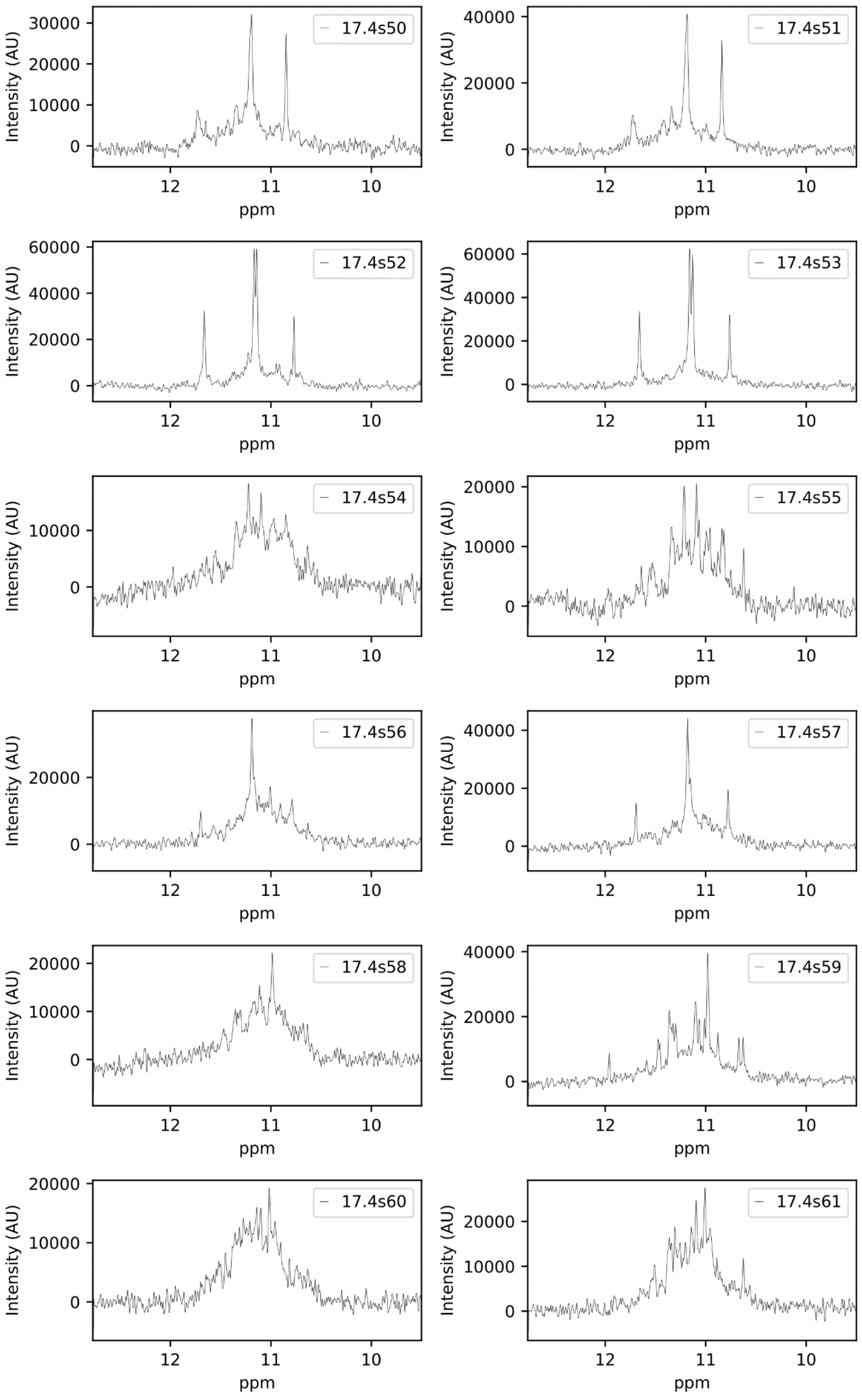
17.4s50
17.4s51
17.4s52
17.4s53
17.4s54
17.4s55
17.4s56
17.4s57
17.4s58
17.4s59
17.4s60
17.4s61
Intensity (AU)
ppm

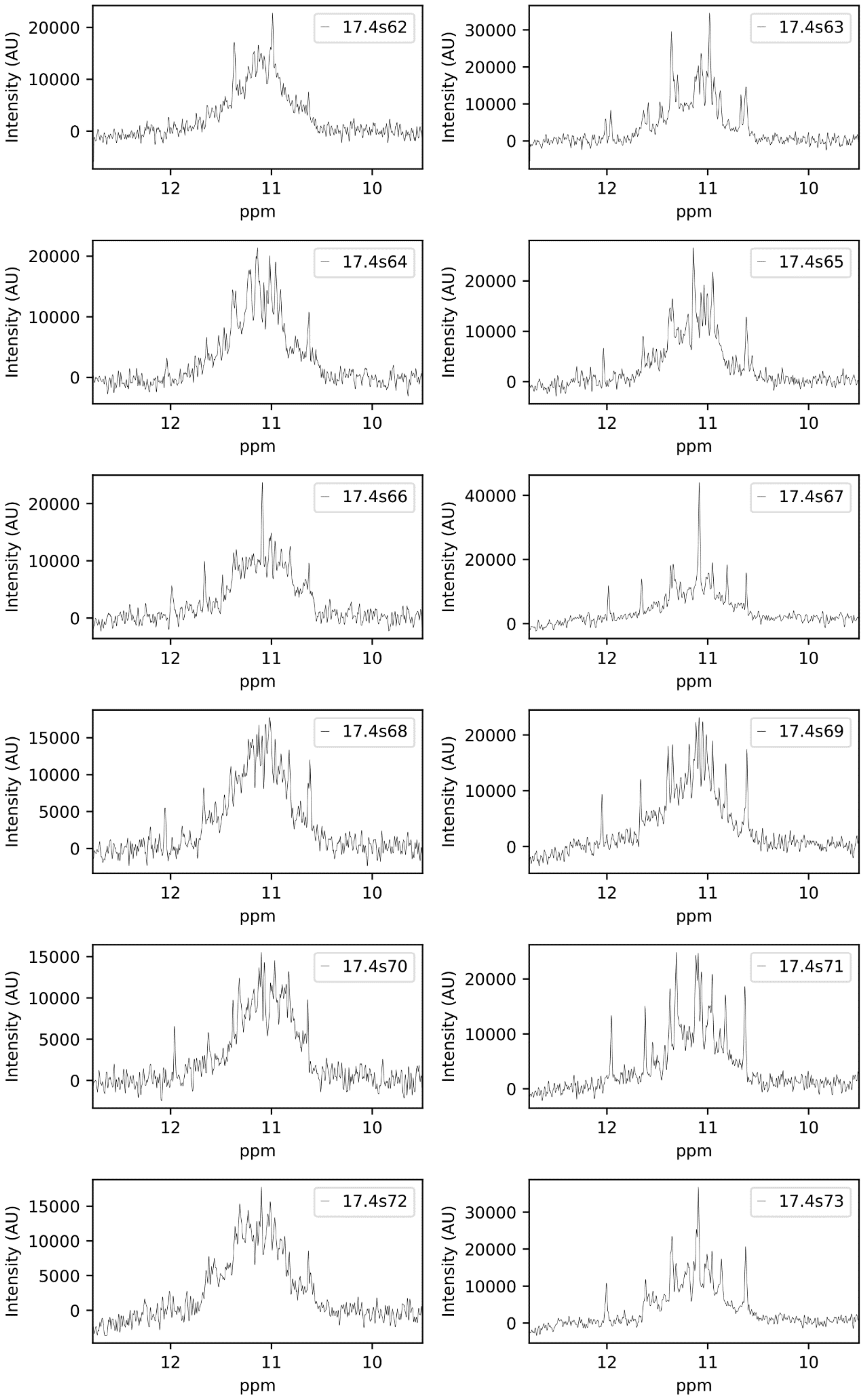
17.4s62
17.4s63
17.4s64
17.4s65
17.4s66
17.4s67
17.4s68
17.4s69
17.4s70
17.4s71
17.4s72
17.4s73
Intensity (AU)
ppm

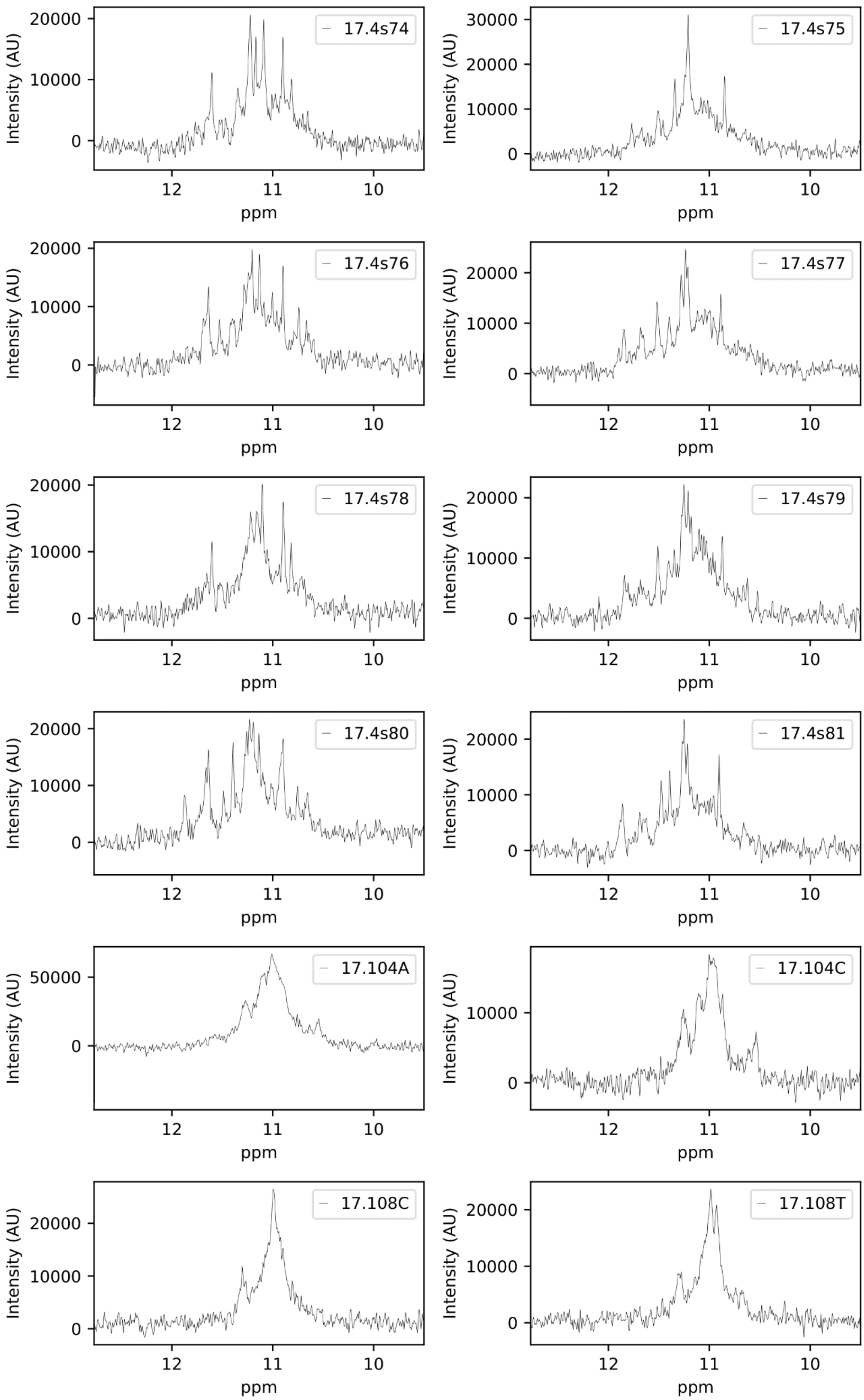
17.4s74
17.4s75
17.4s76
17.4s77
17.4s78
17.4s79
17.4s80
17.4s81
17.104A
17.104C
17.108C
17.108T
Intensity (AU)
ppm

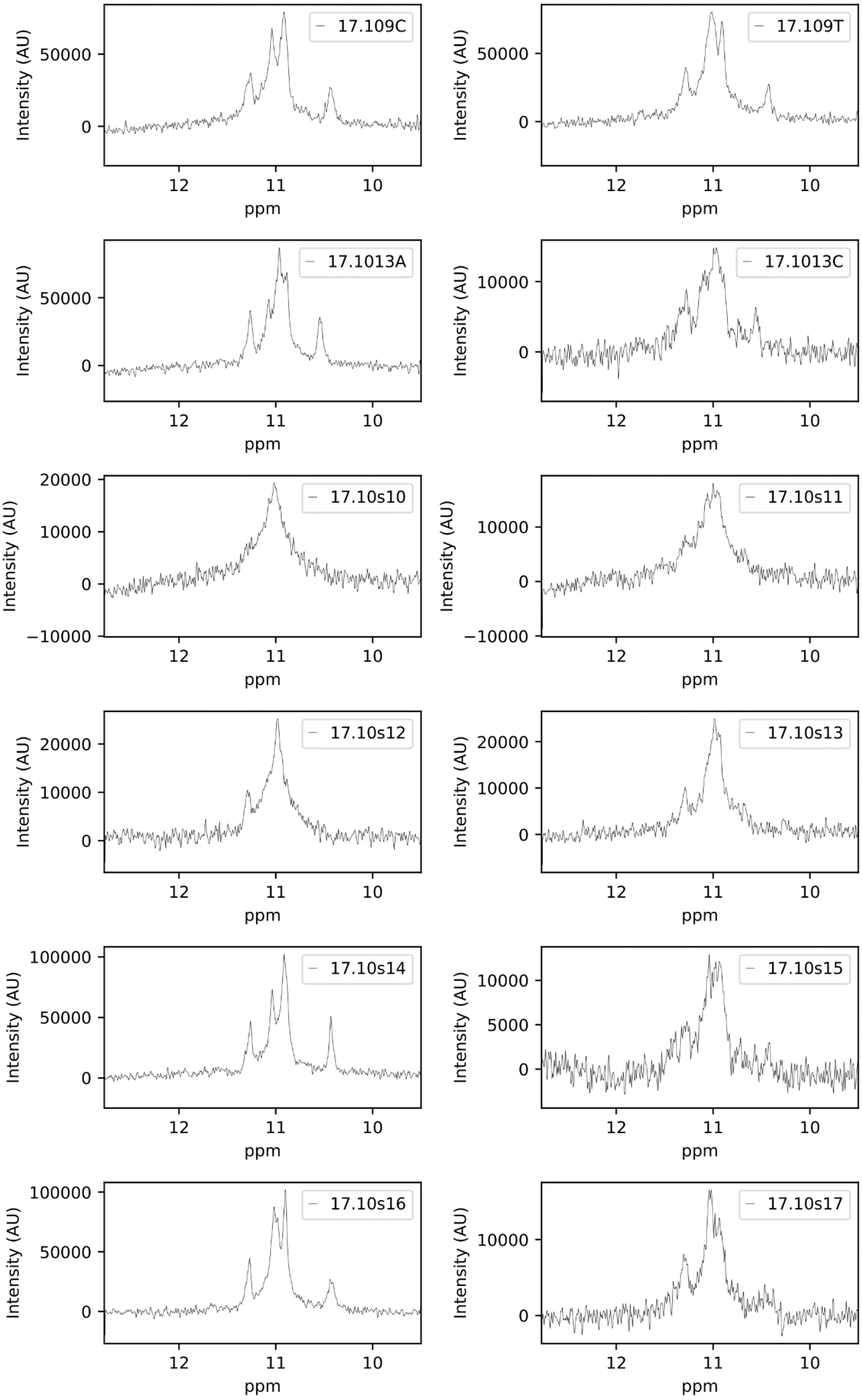
17.109C
17.109T
17.1013A
17.1013C
17.10s10
17.10s11
17.10s12
17.10s13
17.10s14
17.10s15
17.10s16
17.10s17
Intensity (AU)
ppm

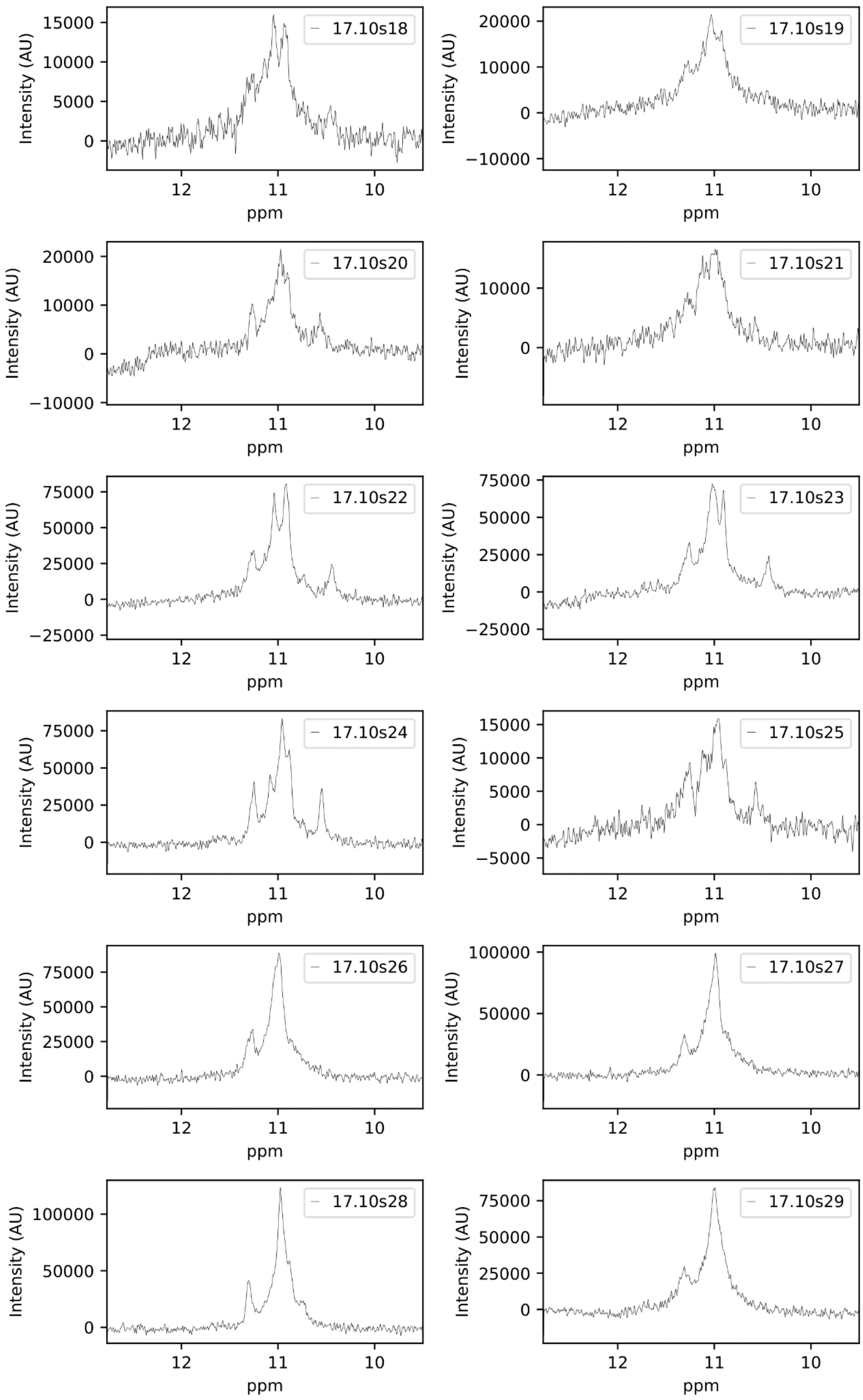
17.10s18
17.10s19
17.10s20
17.10s21
17.10s22
17.10s23
17.10s24
17.10s25
17.10s26
17.10s27
17.10s28
17.10s29
Intensity (AU)
ppm
12
11
10

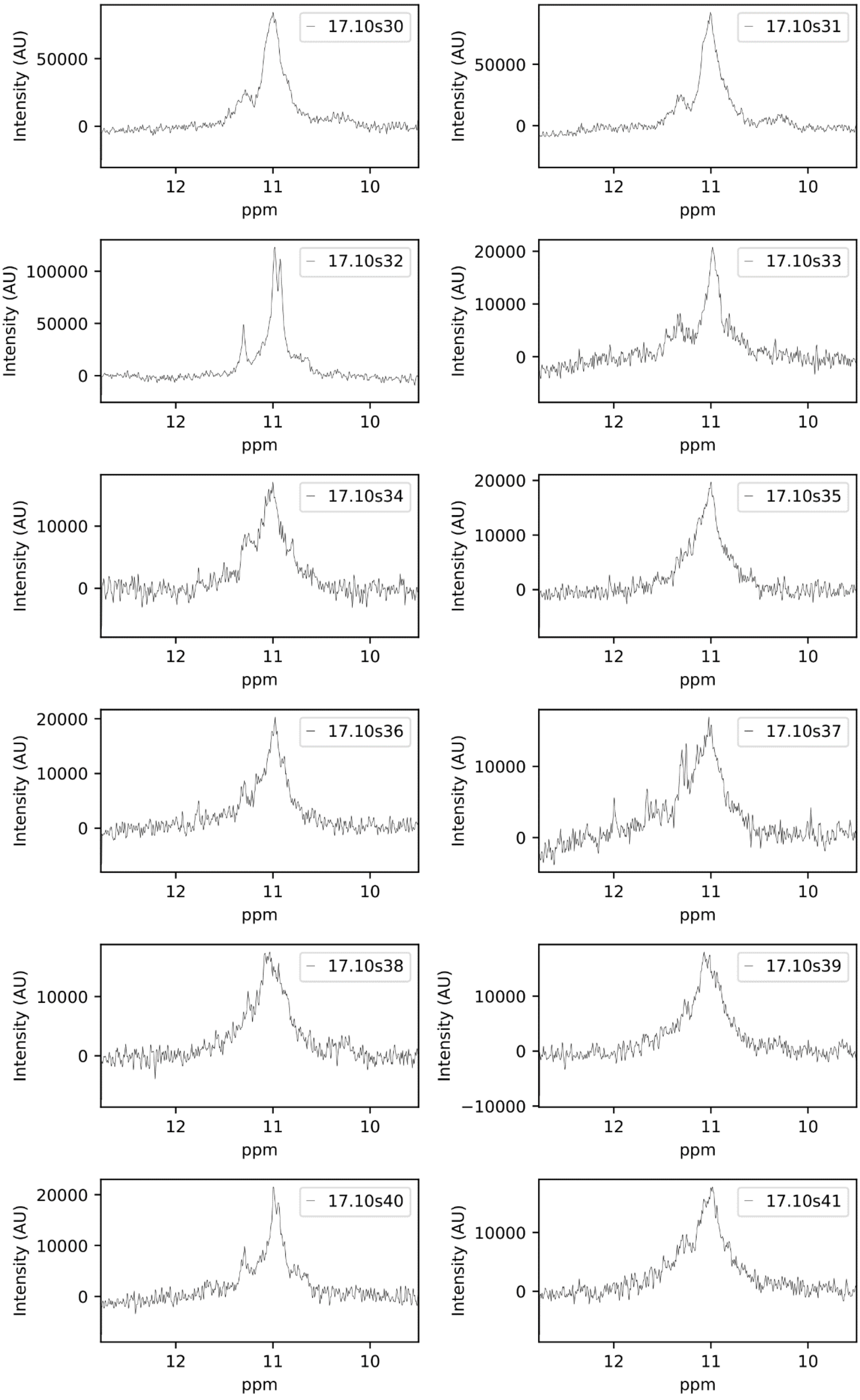
17.10s30
17.10s31
17.10s32
17.10s33
17.10s34
17.10s35
17.10s36
17.10s37
17.10s38
17.10s39
17.10s40
17.10s41
Intensity (AU)
ppm

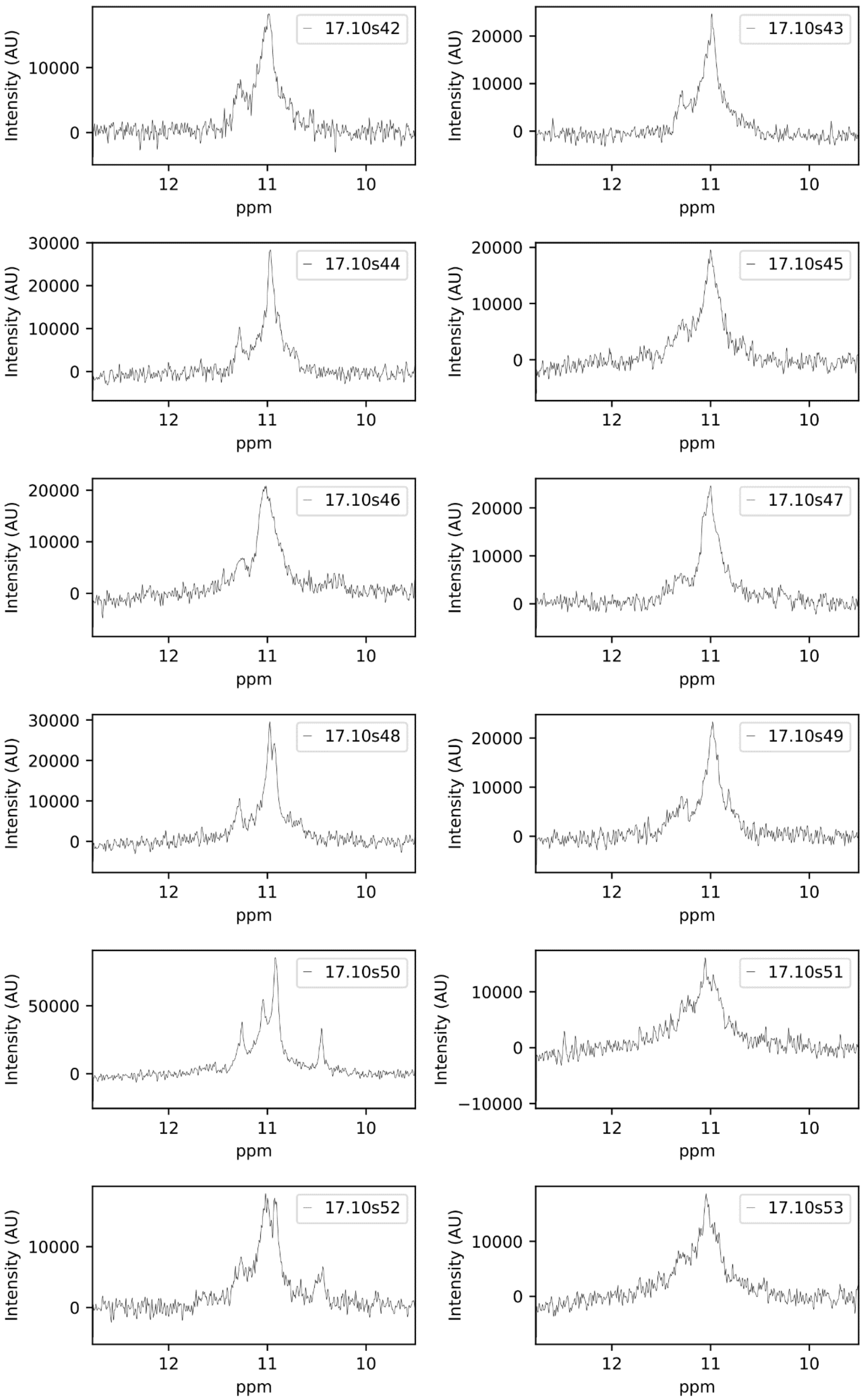
17.10s42
17.10s43
17.10s44
17.10s45
17.10s46
17.10s47
17.10s48
17.10s49
17.10s50
17.10s51
17.10s52
17.10s53
Intensity (AU)
ppm
12
11
10
0
10000
20000
30000
50000
−10000

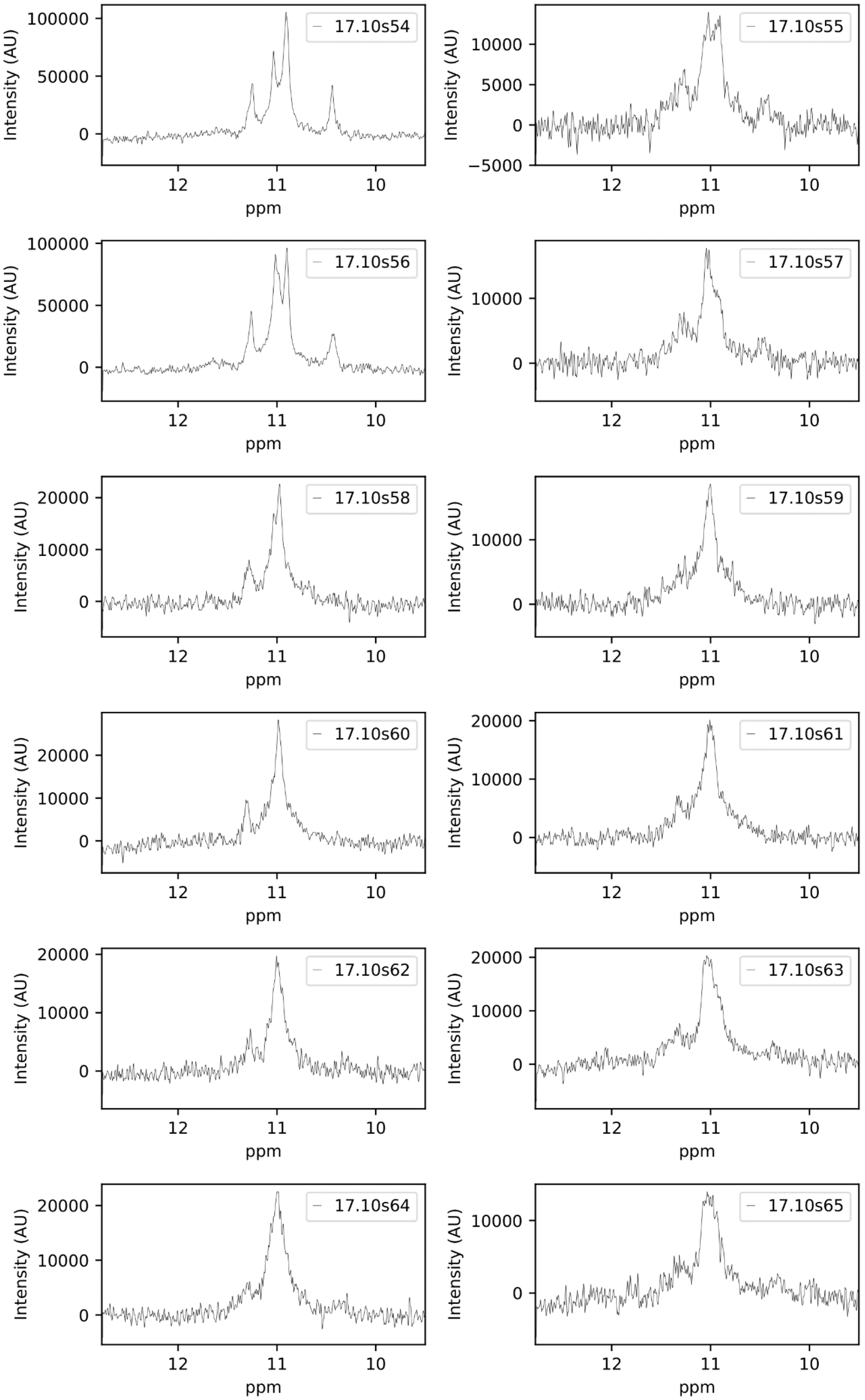
17.10s54
17.10s55
17.10s56
17.10s57
17.10s58
17.10s59
17.10s60
17.10s61
17.10s62
17.10s63
17.10s64
17.10s65
Intensity (AU)
ppm

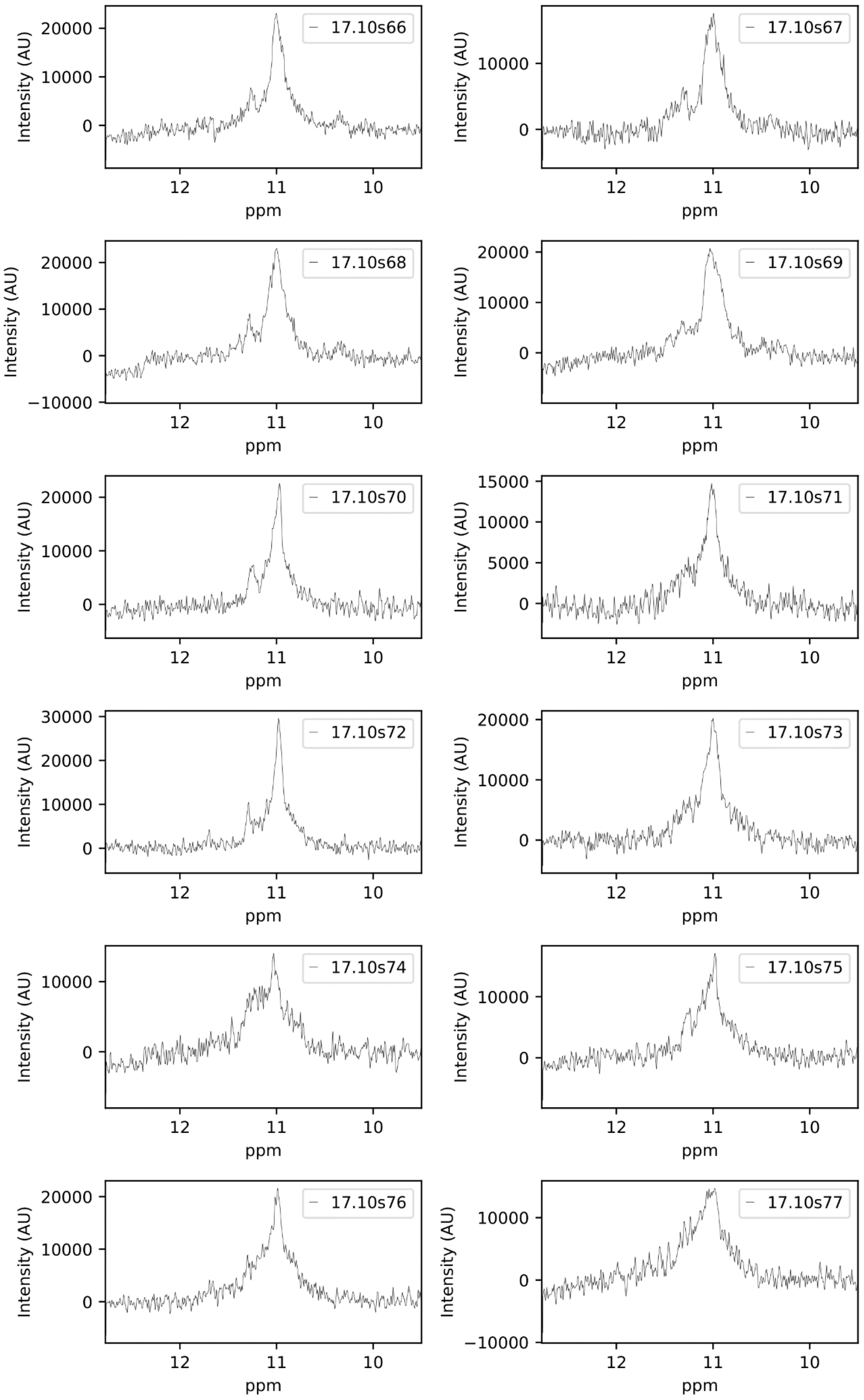
17.10s66
17.10s67
17.10s68
17.10s69
17.10s70
17.10s71
17.10s72
17.10s73
17.10s74
17.10s75
17.10s76
17.10s77
Intensity (AU)
ppm

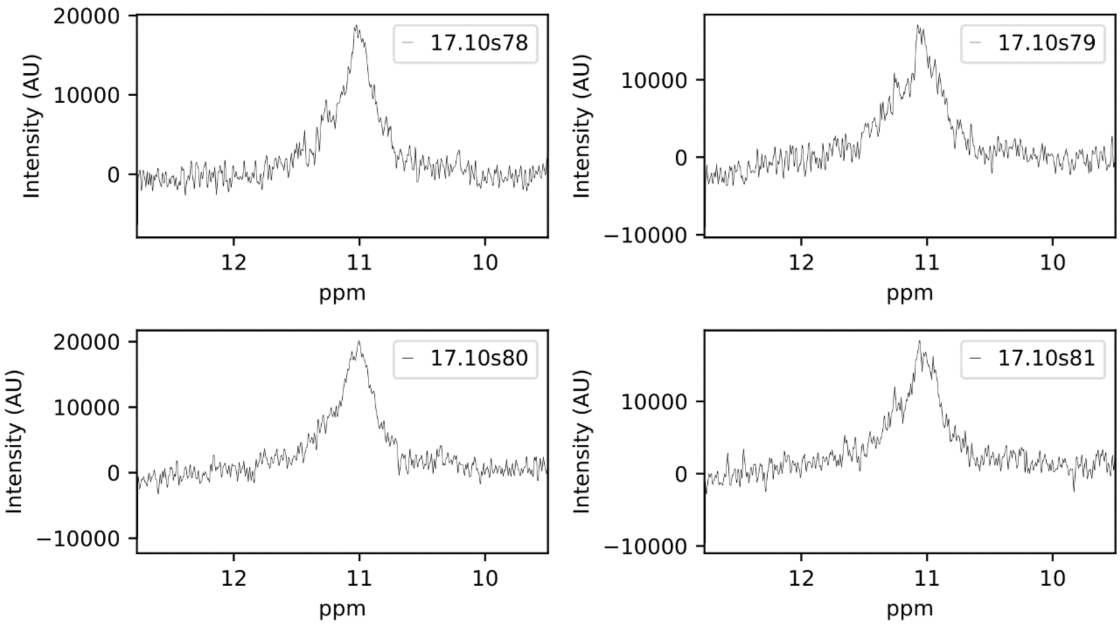
17.10s78
17.10s79
17.10s80
17.10s81
Intensity (AU)
ppm
20000
10000
0
−10000
12
11
10

References

[15] T. Majerová, T. Streckerová, L. Bednárová and E. A. Curtis, "Sequence requirements of intrinsically fluorescent G-quadruplexes," *Biochemistry,* vol. 57, p. 4052–4062, 2018.

[20] S. Kolesnikova, M. Hubálek, L. Bednárová, J. Cvačka and E. A. Curtis, "Multimerization rules for G-quadruplexes," *Nucleic acids research,* vol. 45, p. 8684–8696, 2017.

[21] S. Kolesnikova, P. Srb, L. Vrzal, M. S. Lawrence, V. Veverka and E. A. Curtis, "GTP-dependent formation of multimeric G-quadruplexes," *ACS Chemical Biology,* vol. 14, p. 1951–1963, 2019.

[22] K. Švehlová, M. S. Lawrence, L. Bednárová and E. A. Curtis, "Altered biochemical specificity of G-quadruplexes with mutated tetrads," *Nucleic acids research,* p. gkw987, 2016.

[19] M. Volek, S. Kolesnikova, K. Svehlova, P. Srb, R. Sgallová, T. Streckerová, J. A. Redondo, V. Veverka and E. A. Curtis, "Overlapping but distinct: a new model for G-quadruplex biochemical specificity," *Nucleic acids research,* vol. 49, p. 1816–1827, 2021.
